# Cheminformatics Models for Inhibitors of *Schistosoma mansoni* Thioredoxin Glutathione Reductase

**DOI:** 10.1155/2014/957107

**Published:** 2014-11-25

**Authors:** Sonam Gaba, Salma Jamal, Open Source Drug Discovery Consortium, Vinod Scaria

**Affiliations:** ^1^GN Ramachandran Knowledge Center for Genome Informatics, CSIR Institute of Genomics and Integrative Biology, Mall Road, Delhi 110007, India; ^2^CSIR Open Source Drug Discovery Unit, Anusandhan Bhawan, 2 Rafi Marg, Delhi 110001, India

## Abstract

Schistosomiasis is a neglected tropical disease caused by a parasite *Schistosoma mansoni* and affects over 200 million annually. There is an urgent need to discover novel therapeutic options to control the disease with the recent emergence of drug resistance. The multifunctional protein, thioredoxin glutathione reductase (TGR), an essential enzyme for the survival of the pathogen in the redox environment has been actively explored as a potential drug target. The recent availability of small-molecule screening datasets against this target provides a unique opportunity to learn molecular properties and apply computational models for discovery of activities in large molecular libraries. Such a prioritisation approach could have the potential to reduce the cost of failures in lead discovery. A supervised learning approach was employed to develop a cost sensitive classification model to evaluate the biological activity of the molecules. Random forest was identified to be the best classifier among all the classifiers with an accuracy of around 80 percent. Independent analysis using a maximally occurring substructure analysis revealed 10 highly enriched scaffolds in the actives dataset and their docking against was also performed. We show that a combined approach of machine learning and other cheminformatics approaches such as substructure comparison and molecular docking is efficient to prioritise molecules from large molecular datasets.

## 1. Introduction

Schistosomiasis is a disease caused by Platyhelminths parasite belonging to the species* Schistosoma* and genus trematodes. It is the most important water based disease [1] and affects the intestine and urinary tract. The disease has a major prevalence in the tropical and subtropical countries of the world and is considered as one of the neglected tropical diseases. Schistosomiasis affects over 200 million people annually with almost over 85% of the infections occurring in Africa alone [2]. The disease has a characteristically low mortality and high morbidity primarily due to the chronic nature of the infection and in many regions of the tropics; schistosomiasis is only next to malaria as a cause of morbidity [3]. The therapeutic repertoire of drugs available used to treat infections due to this pathogen is highly limited with praziquantel being the maximally used and first line of treatment [4]. A single oral dose of the drug is extremely effective against the pathogen and has also been recommended for use in areas of high incidence [5, 6]. The drug was originally developed in the 1970s and is relatively inexpensive and has been effectively used in the treatment of the disease. However novel drug-resistant strains have emerged [7]. In the light of the increasing incidences of drug resistant schistosomiasis, there is an urgent and unmet need to discover novel therapeutic agents against this pathogen. Several other drugs such as artemether (an antimalarial drug), oxamniquine, and metrifonate have been used but with limited success.

Recent studies have pointed towards thioredoxin glutathione reductase as one of the well-characterized alternate targets for drug development for schistosomiasis [8]. This selenium containing enzyme reduces the harmful oxygen radicals produced by human body and therefore the protein is essential for survival of the parasite. The protein is also involved in protein folding control, regulation of various enzymes and transcription factors, and provides electrons in deoxyribonucleotide synthesis. Contrary to the two sets of proteins which modulate thioredoxin and glutathione redox systems in other eukaryotes, schistosomes have the two functions incorporated into a single enzyme that protects the pathogen from the oxidative stress and damage induced by the host [1]. The active site of protein consists of three cysteine dimmers or thiol centers Cys 28 Cys 29, Cys 154 Cys 159, and Cys 596 Cys 597 wherein FAD binds near Cys 154 and Cys 159 moieties and transfers electrons from Cys 154 Cys 159 dimer to Cys 596 Sec 597 dimer upon NADPH binding [9]. Cysteine 596 and selenocysteine 597 are present on flexible C terminal arm and can transfer hydrogen to Cys 28 Cys 29 or to the oxidized substrate. Therefore selenocysteine plays an important role in redox mechanism of the enzyme. Additionally, a recent study has provided further evidence for the criticality of this system in the survival of the pathogen through antisense based knockdown systems [10]. Molecules including auranofin have been observed to show antihelminthic activity through the inhibition of the enzyme [11].

The availability of high-throughput screening methodologies and resources has provided a quantum difference from conventional methodologies of drug discovery [12]. The high-throughput assays have provided immense data for prioritizing molecules for in-depth study, especially in the case of infectious diseases [13] and specifically tropical diseases [14, 15]. Computational learning of molecular properties of molecules from such large datasets also provides us with an opportunity and means to build models for recognition of molecular features of molecules with a given biological activity. These models can be used to screen efficiently large molecular structure datasets using* in silico* approaches. Such methodologies have been reported previously, including tuberculosis [16, 17] and malaria [18] diseases and also for target-specific assays like RNA-binding [19]. Recent efforts have made available a large repertoire of molecular activities screened for inhibition of thioredoxin glutathione reductase of* Schistosoma mansoni* [20, 21]. The availability of such large molecular datasets provides us with a novel opportunity to investigate and understand the molecular properties of actives as well as learn and model the biological activities and use them for the virtual screening of large datasets and other new molecules.

In this study, we report the first comprehensive* in silico* analysis of the inhibitors developed for thioredoxin glutathione reductase. We use a host of approaches like molecular property based supervised learning and clustering based on maximum common substructure (MCS). We also further analyse a set of 10 enriched molecular substructures by using alternate approaches including docking analysis of the enriched molecules into TGR. We show that a combined approach could potentially be used to screen and prioritise molecules from large chemical libraries.

## 2. Material and Methods

### 2.1. Datasets

The dataset corresponding to the AID 485364 was downloaded from PubChem, the public repository of biochemical datasets [22]. The specific dataset includes a repository of inhibitors of the TGR enzyme that have been identified to be crucial for the survival of the flatworm* Schistosoma mansoni* in the human host by providing a unique escape system for the pathogen from the host immune system. These molecules have been shortlisted through a 1536 well-based kinetic high throughput screening assay against TGR and the dataset includes 10,735 actives, 3,31,528 inactives, and 14,558 inconclusive compounds. The three activity categories are based on the PubChem activity score, an evaluation parameter relying on the IC_50_ values, which marks compounds as having a score of 0 as being inactive, 1–39 as inconclusive, and 40–100 as active. The SDF (structure data format) files for the actives and inactives were downloaded separately from the database and the inconclusive compounds were not further considered in the analyses as they could potentially interfere with the predictive abilities of the computational models.

### 2.2. Chemical Descriptors

We used PowerMV, a popular and free software, to compute the chemical descriptors for the molecules which calculates these descriptors in 6 major classes, namely, atom pair, atom pair (Carhart), fragment pair, pharmacophore fingerprint, weighted burden number, and properties [23]. A total of 179 descriptors were generated for the dataset. Atom based descriptors were not used in the evaluation for they are commonly used to identify closely related analogues which was determined to be not suitable in the context of our study. Pharmacophore fingerprints, weighted burden, and properties were used as analysis parameters in our study. Pharmacophore fingerprint is a binary descriptor used to find diverse analogues and includes 147 descriptors while weighted burden and properties are continuous descriptors which contain 24 and 8 descriptors, respectively. The descriptors were generated by PowerMV [5] and the ones which had identical values for all the molecules (active and inactive) were removed from the set as these would not help in classifying the data. The descriptors were selected using RemoveUseless module implemented in Weka. Pharmacophore fingerprints are based on bioisosteric principles which means grouping of molecules which are expected to have the same biological effect. For example, the primary and secondary amines are expected to have the same biological effect as both are positively charged molecules. This includes 6 classes such as negatively charged groups, positively charged groups, hydrogen bond donors, hydrogen bond acceptors, aromatic center, and hydrophobic atoms. All these six classes are important for a biological activity of a molecule. The weighted burden descriptors uses burden connectivity matrix in which one of the properties is placed on diagonal of the matrix and Eigen values are calculated. This includes three important parameters electronegativity, Gasteiger partial charge, and XlogP which are important for intermolecular interactions. Lastly the properties descriptor used in the present study includes polar surface area, molecular weight, blood brain indicator, and bad group indicator which means toxic group. This descriptor gives information about the drug likeness of the molecule which is important aspect in our study. A summary of the descriptors used is mentioned in Supplementary Table 1 available online at http://dx.doi.org/10.1155/2014/957107.

SplitSDFiles, a Perl script based algorithm developed by MayaChemTools [24], was used to split the large SDF files into smaller files owing to the memory problems faced with PowerMV in handling large files. Subsequently, descriptors were generated for the smaller split files using PowerMV which were then concatenated and an additional column appended to the end of each row termed as the “outcome.” This column was either tagged “active” for the active files or “inactive” for the inactive files. The files containing the descriptors for active and inactive compounds were saved in the  .*csv* (comma delimited) file format.

### 2.3. Removal of Useless Attributes

Nondiscriminatory attributes having the same value for all the compounds of the dataset were removed because these attributes would not help in classifying compounds. This step reduced the dimensionality of the dataset by decreasing the number of descriptors from 179 to 154.

### 2.4. Creation of the Training and the Test Sets

We used a 5-fold cross-validation approach whereby the dataset comprising of both actives and inactives was divided into 80 percent training set and 20 percent test set.

### 2.5. Data Mining

We used Weka (Waikato Environment Knowledge Analysis) version 3.6.8 toolkit for our analysis which is a data mining tool that can be used for processing, classification, clustering, regression, association, and visualization [25]. In our study, Weka was used to generate the classifier models as well as the conversion of the test and training files from the* csv* format to the* arff* (attribute relation file format) format.

We used popular classifiers, naïve Bayes [26], random forest [27], and J48 [28], as these have been previously used extensively for the chemoinformatics data mining applications [16–19].

### 2.6. Cost Based Learning

The data we used in our study was highly imbalanced given the significantly low number of actives as compared to the inactives [29]. The standard naïve Bayes, random forest, and J48 classifiers do not correctly classify the imbalanced data since these give equal weightage to both the active and inactive classes. The misclassification errors cost equally for the standard classifiers. Therefore, the cost sensitive classification method was used which has two categories. The first category includes the direct method which makes use of the misclassification cost in the algorithm itself and the second is metalearning, which changes the base classifier into the cost sensitive classifier. There are two approaches for the latter: one is MetaCost and the other is the cost sensitive classifier (CSC) [30]. MetaCost algorithm includes the resampling of the training set and a classifier model is generated based on these resamples, resulting in the relabelling of the training instances based on the votes of ensemble. Then a new model is generated based on this relabelled training set whereas in cost sensitive classifier, a cost insensitive algorithm is used for estimating the probability of the test cases which then determines the class of a test instance. A misclassification cost was also applied to the false negatives in order to reduce them and there was no defined rule used for setting it, but rather dependant on the base classifier used. Continuous increment of the misclassification cost on the false negatives would increase both the true positives and false positives. Therefore, an upper limit of 20% of false positives was set to provide space for misclassification cost to increase until the false positives reach a value of 20%.

### 2.7. Evaluation of the Models

Various statistical parameters were used in the evaluation of the performance of the models generated. These included sensitivity, specificity, balanced classification rate (BCR), Matthews correlation coefficient (MCC), receiver operating characteristics (ROC), and area under the curve (AUC).* Sensitivity* may be defined as the ability of a classifier to correctly identify positives and can be put as the ratio of true positives to the sum of the true positives and false negatives TP/(TP + FN).* Specificity* is defined as the ability of a classifier to correctly identify the negatives. Mathematically, it is the ratio of the true negatives to the sum of the true negatives and false positives TN/(TN + FP). Practically, there is no classifier which gives 100 percent sensitivity or specificity because an increase in sensitivity results in a compromise on the specificity and vice versa.* Balanced classification rate (BCR)* represents the trade-off between sensitivity and specificity which gives the balanced accuracy and is calculated as 0.5x (specificity + sensitivity).* Matthews correlation coefficient (MCC)* is the mathematical measure of the correlation between the observed and predicted results or in our case classification. It is the best representative measure of a confusion matrix in one value with values lying between −1 (that is by random chance) and +1 (perfect agreement). A value of zero is indicative of no correlation at all. The* receiver operating characteristics (ROC)* are a plot of the sensitivity versus 1 − specificity. It is a well-established method to represent the trade-off between sensitivity and specificity.* AUC (area under the curve)* as the name suggests is the area under the ROC curve and has values between zero and 1. It defines the probability that a classifier will classify a test instance in the correct class.

### 2.8. Clustering Using Maximum Common Substructure (MCS) Approach

For clustering and substructure analysis, modules of JChem suite of ChemAxon were used. JChem is a chemoinformatics toolkit required for the conversion of structure files in different formats, converting structures from 2D to 3D, generating descriptors, clustering molecules, and the analysis of structures based on various similarity parameters. Prior to clustering, the active and inactive files were downloaded from PubChem and converted to 3D using the molconvert module of JChem.

### 2.9. Substructure Analysis and Enrichment Factor Calculation

LibraryMCS (LibMCS), available from ChemAxon, based on hierarchical clustering algorithm was used to cluster the molecules [31]. LibMCS initially places all the structures at the base level and then clusters in a hierarchical manner on the basis of maximum common substructure. The clusters formed are disjoint meaning that one molecule belongs to one cluster only. In the top level, singletons (molecules which did not belong to any cluster) were removed and all the remaining scaffolds were saved. We used* jcsearch* module available from JChem to find the frequency of occurrence of the scaffolds in actives and inactives datasets [32]. We further calculated the frequency of scaffolds in actives and inactives and the enrichment factor. We used Chi-square test for assessing the significance of enrichment.

### 2.10. Docking and Analysis

The crystal structure of* Schistosoma mansoni* thioredoxin glutathione reductase (PDB ID: 2V6O) was retrieved from Protein Data Bank (PDB) for docking studies [9]. We selected actives having any one of the enriched substructures out of the 10 enriched substructures giving us a subset of 184 active molecules. Docking analysis was performed for these molecules across the FAD active site of thioredoxin glutathione reductase (TGR) using DOCK 6. DOCK 6 is a suite of modules used for receptor preparation, ligand preparation, and grid generation and docking. Hydrogens were removed and the protein (PDB ID 2V6O) was prepared using UCSF Chimera [33]. Chimera is open source molecular visualization software that enables the visualization of 3D chemical structures including X-ray crystal structures of proteins as well as small molecule structures of drug leads. A molecular surface of the protein was then generated using Connolly surface by Chimera's write DMS tool and the resulting molecular surface file was used as input to create overlapping spheres (min radius = 1.4 Å and max radius = 5 Å) from the molecular surface of the protein using the Sphgen module of DOCK 6 [34]. Subsequently, an active site was defined using FAD as the desired location and taking the area of radius 15 Å around it using a program sphere selector. This radius was selected so as to include all the critical residues interacting with FAD in the grid. Spheres which did not fit the active site were removed manually by editing the sphere file resulting in the generation of an accurate grid covering only the active site of the TGR protein. A box was then generated around this specific region by the program Showbox which defines the location of the grid to be generated. The program grid enabled the calculation of the contact and electrostatic potentials of the grid and provided the outputs in grid.cnt and grid.eng files, respectively [35]. The final step in docking studies required that the ligands be available as mol2 files, which was carried out using obabel [36]. Eventually rigid docking was done using the DOCK 6 module with the output presented as grid scores.

### 2.11. Analysis of the Zinc Dataset

A subset termed as the “drug-like molecules” present within the zinc dataset was downloaded which contained a total of 15,798,630 molecules and after removing redundancy, we obtained a set of 11,721,018 molecules which were then tested using our best classifier—the random forest model. A cost of 860 was implemented using the same machine learning tool Weka 3.6.8 and only those molecules that were predicted to be active were selected and then converted into the 3D SDF format using the tool molconvert (JChem) and used further for the occurrence of the ten enriched scaffolds (Table 2) using jcsearch. Only those molecules which were found to have the enriched substructures were finally docked onto the active site of TGR. Further, 10,000 random molecules were taken from the set of molecules which were predicted as inactive by the model and were docked into the active site of TGR. This gave us two sets of scores, one set corresponding to the scores for the actives having substructures and the other set for the random 10,000 molecules predicted to be inactive by the model. Finally, a two-sample *Z*-test was performed as an evaluation parameter to determine the statistical significance of the difference between the two sets.

## 3. Results and Discussions

### 3.1. Comparison of Models

The results of training and testing of all the platforms used are displayed in Table 1. Because the dataset was highly imbalanced we applied misclassification cost on false negatives, but as this benefit systematically increases false positives also, an empirical limit of 20 percent false positive classification was applied.

**Figure 1:**
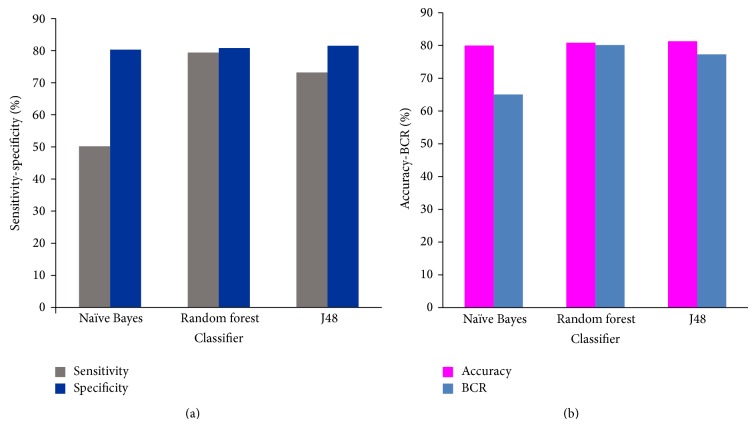
Comparison of the performance of the models of naïve Bayes, random forest, and J48 based on (a) sensitivity and specificity and (b) accuracy and BCR.

**Figure 2:**
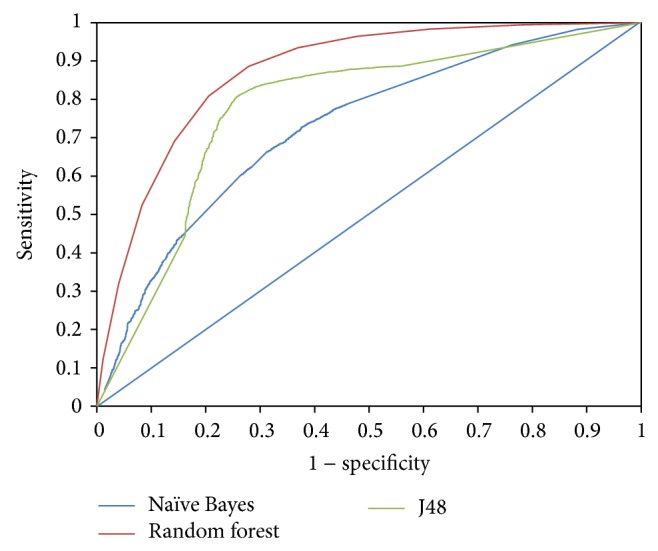
Comparison of the performance of three classifiers based on ROC (receiver operating characteristics) curve.

Random forest (RF) was determined to be the most sensitive classifier with a sensitivity attainment of 79.4% followed by J48 (73.2%) and lastly naïve Bayes (50.2%). The sensitivity versus specificity ratios computed for the different data mining algorithms are represented in Figure 1. The BCR was also computed as a measure of the performance of the models in imbalanced datasets. As evident from Table 1, RF had the best BCR. The accuracy versus BCR plot is also represented in Figure 1. ROCs for each of the models are displayed in Figure 2. Lastly, the AUC for each of the methods was also determined with the maximum AUC for RF (AUC = 0.87) and the lowest for naïve Bayes (AUC = 0.72).

### 3.2. Substructure Analysis

**Figure 3:**
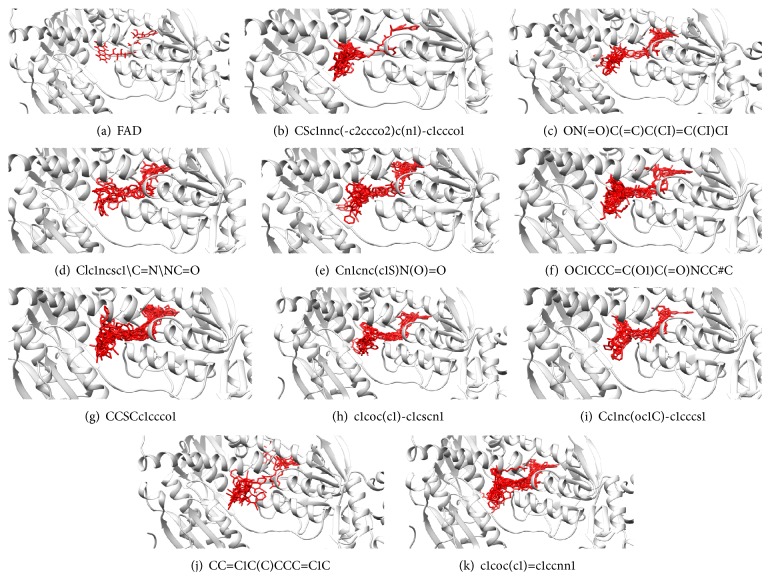
Docked molecules. (a) FAD and all the actives (b)–(k) corresponding to 10 enriched scaffolds in the enzyme TGR.

Substructure analysis of the actives was also done to prioritize the commonly occurring substructures that contributed the most to the activity of the molecules. Using LibMCS a total of 10,735 compounds belonging to the active set were clustered. A total of 2,622 clusters were generated up to 5 hierarchical levels. The 444 clusters that were at the top level 5 were used in further studies, of which 164 singletons were removed resulting in a total of 280 substructures. Further, the occurrence and frequency of occurrence of the substructures in both the actives and inactives were also computed and 177 substructures having a frequency greater than 0.1% in actives were shortlisted for further analysis. Such low frequency was set so as to not leave any enriched scaffolds. The enrichment factor and Chi-square statistics were also evaluated based on the frequencies of occurrence of the substructures in both the active and inactive datasets (Supplementary Table 2). We further selected the substructures having an empirical enrichment factor above 10 and a statistically significant Chi-square distribution (*P* value less than 0.01) to obtain a comprehensive list of 10 substructures or scaffolds (Table 2). The docked molecules corresponding to each of the substructures were carefully compiled and analyzed by visual inspection. The docked images of molecule FAD and the 184 actives corresponding to the 10 enriched scaffolds in the FAD active site of TGR are shown in Figure 3. An Additional File contains the dock scores of these 184 actives.

### 3.3. Independent Evaluation of the Models Using Docking Approaches

The paucity of independent high-throughput experimental datasets for inhibitors of* Schistosoma* TGR precluded our attempts to independently evaluate the general applicability of our models on large datasets. We have circumvented this deficiency by using an independent dataset of molecules collated within the ZINC database and marked as “drug-like.” This dataset contains 11,721,018 unique molecules. We used the RF prediction model on the ZINC dataset and identified 2,061,210 molecules as actives and further filtered this dataset using the enriched substructures approach which yielded 14,354 molecules. We used a docking-based approach employing the DOCK software and the target protein crystal structure PDB: 2V6O to evaluate our prediction methodology. Docking analysis was performed on the 14,354 molecules and an additional 10,000 random molecules predicted as inactive by the model with the docking scores obtained being compared and evaluated using *Z*-test. Our analysis showed that the active molecules predicted in our dataset had a significantly different docking score compared to the predicted inactives (*Z*-test *P* value < 0.0001), suggesting that the prediction model could be potentially used for effectively prioritizing potential actives from large datasets. We have also taken random 9999 molecules from ZINC dataset which were predicted as active by random forest model but which do not contain any of the enriched substructures and docking was done for these molecules using the same tool dock 6. These docking scores were compared with the 10,000 random molecules which were predicted as inactive by random forest using two-sample *Z*-test and it resulted in *P* value < 0.0001 suggesting that the two sets of predicted actives and inactives by random forest are significantly different and RF and docking scores are correlated. Moreover, the PubChem assay on which random forest was built is based on the activity of inhibitors against the TGR protein which also depends on the binding energy of inhibitor and TGR protein. Dock scores of 10,000 random inactives, 14,354 actives containing one of the substructure, and random 9999 molecules which are predicted as active by RF but do not contain any of the substructure are presented in Additional File.

## 4. Conclusions

Schistosomiasis is still one of the major causes of morbidity, currently affecting over 200 million people, majorly in the African continent [2]. The disease has been largely categorised as a neglected tropical disease [2], owing to poor research, funding, and initiatives for drug discovery. Accelerating the drug discovery process for neglected tropical disease would require the development of novel methodologies and tools for significantly cutting costs in prioritising molecules, which could be potentially taken up for further testing.* In silico* methodologies have been widely suggested for prioritising molecules for other diseases such as tuberculosis and malaria [16–18]. These methodologies rely largely upon the availability of well-characterized datasets for specific biological activities. The recent availability of datasets from high-throughput biological screens for specific biological activities offers a new opportunity to build computational models to screen for potential actives from libraries of compounds* in silico* [13]. Such* in silico* approaches, if highly accurate, could be scalable, fast, and cost-effective, with a potential to significantly reduce the cost and time for prioritising the actives.

In this paper, we describe a computational methodology and model for predicting the specific molecular activities of particular scaffolds against the TGR protein, a well-characterized alternative drug target in the therapy of infections caused by* Schistosoma mansoni*. We have used the machine learning approach based on the generation of molecular descriptors for a large set of molecules available in public domain to screen for this specific activity. We also used the substructure based approach to identify enriched substructures that contributed significantly to the activity of the molecules. We show that the methodology used is of acceptable accuracy, with accuracy estimates close to 80%. We also apply a proof of principle application for the approach and independent validation by prioritising a subset of molecules from the “drug-like molecules” library of the ZINC dataset [37]. Using an independent docking based approach, we also show that the molecules predicted through the pipeline have significantly better docking scores, suggesting that the methodology could be applied effectively to prioritise molecules from large chemical libraries* in silico*. However, the present study is not without caveats with the major caveat being the paucity of independently comparable large datasets of high-throughput screens that would allow us to evaluate the generalisability of our model and have an independent evaluation of the accuracy estimates. We have tried to overcome this challenge by using a docking based approach to verify the methodology and show that the predicted active molecules prioritised using our approach have a significantly better docking score (*Z*-test *P* value < 0.001) as compared to randomly picked predicted inactive molecules from the same dataset.

In summary, we provide one of the first computational predictive models for prioritising molecules with inhibitory activities against the* Schistosoma* TGR protein. We also make available the model with a detailed set of instructions on formatting and screening molecules using the computational model. We hope this would be widely used by researchers working in the area to prioritise molecules for screening as well as enable the accelerated drug development and discovery for neglected tropical diseases such as schistosomiasis.

## Supplementary Material

Supplementary Table 1 contains the detailed information of all descriptors included in the three major descriptors (Pharmacophore fingerprints, Weighted burden and Properties) used in our study. Supplementary Table 2 shows 177 scaffolds which have frequency greater than 0.1 percent in actives. These scaffolds were selected for further analysis. Additional File contains the docking scores of molecules from AID485634 and ZINC which were predicted active by Random Forest model and contains one of the enriched scaffolds. It also shows the docking scores of ZINC molecules predicted inactive by the model and the ones which were predicted active but do not contain any enriched substructure.

## Figures and Tables

**Table 1:** Comparison of sensitivity, specificity, accuracy, and balanced classification rates and Matthews correlation coefficient for each of the classifiers used in the present study.

Classifier	Cost	TP rate	FP rate	BCR	MCC
Naïve Bayes	10	50.3	19.1	65	0.13
Random forest	860	79.4	19.1	80.1	0.25
J48	150	73.2	18.5	77.3	0.23

**Table 2:** The Enriched scaffolds having *P* < 0.01 and enrichment factor >10 that are corresponding to 184 actives.

Scaffolds	Matches in Actives (10735)	Matches in Inactives (331528)	Actives (without motif)	Inactives (without motif)	Chi-square	*P*-value	Enrichment factor
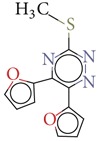	13	1	10722	331527	370.9611	1.16*E* − 82	401.48

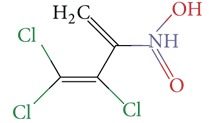	16	4	10719	331524	388.9498	1.40*E* − 86	123.53

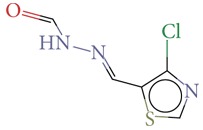	11	4	10724	331524	243.3006	7.50*E* − 55	84.93

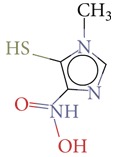	20	11	10715	331517	384.4568	1.33*E* − 85	56.15

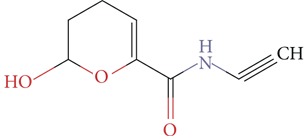	21	20	10714	331508	312.045	7.83*E* − 70	32.43

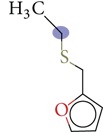	28	32	10707	331496	374.2882	2.18*E* − 83	27.02

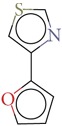	21	27	10714	331501	260.64	1.24*E* − 58	24.02

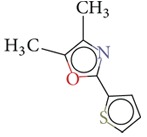	12	21	10723	331507	119.9333	6.54*E* − 28	17.65

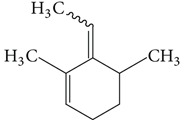	12	30	10723	331498	89.44623	3.15*E* − 21	12.35

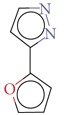	30	92	10705	331436	184.8912	4.15*E* − 42	10.0
