# Supplementary material for: Cheminformatics Models for Inhibitors of Schistosoma mansoni Thioredoxin Glutathione Reductase
Source: ScientificWorldJournal. 2014 Nov 25;2014:957107. doi: 10.1155/2014/957107 (PMC4275605; doi:10.1155/2014/957107)
Supplement: Supplementary file 1 — Supplementary Table 1 contains the detailed information of all descriptors included in the three major descriptors (Pharmacophore fingerprints, Weighted burden and Properties) used in our study. Supplementary Table 2 shows 177 scaffolds which have frequency greater than 0.1 percent in actives. These scaffolds were selected for further analysis. Additional File contains the docking scores of molecules from AID485634 and ZINC which were predicted active by Random Forest model and contains one of the enriched scaffolds. It also shows the docking scores of ZINC molecules predicted inactive by the model and the ones which were predicted active but do not contain any enriched substructure. [file 957107.f1.zip › Additional File.pdf]

ZINC ID	Score
ZINC00029419	-35.4065
ZINC00031384	-43.9474
ZINC00033431	-40.1489
ZINC00035479	-43.3154
ZINC00035767	-32.9451
ZINC00036001	-41.5307
ZINC00036591	-34.2997
ZINC00039770	-29.1791
ZINC00041425	-56.3369
ZINC00043595	-22.2464
ZINC00045275	-35.8514
ZINC00046095	-49.7614
ZINC00046317	-38.3916
ZINC00046974	-38.827
ZINC00047437	-39.2751
ZINC00050403	-35.9263
ZINC00050690	-33.2651
ZINC00052187	-23.4364
ZINC00053229	-34.5762
ZINC00053333	-39.4016
ZINC00055289	-33.2697
ZINC00056824	-31.7013
ZINC00058746	-42.3069
ZINC00060296	-33.0636
ZINC00060583	-39.0426
ZINC00061237	-45.0092
ZINC00062303	-42.8968
ZINC00064741	-29.6017
ZINC00069043	-34.8322
ZINC00071347	-40.5886
ZINC00074748	-24.9066
ZINC00077036	-42.5125
ZINC00079639	-39.1211
ZINC00079780	-36.8772
ZINC00080961	-41.6617
ZINC00081961	-32.0892
ZINC00082693	-36.6511
ZINC00082693	-36.7642
ZINC00084059	-41.1837
ZINC00088053	-27.9192
ZINC00088053	-29.564
ZINC00088053	-33.2721
ZINC00088377	-23.4829

ZINC00088377	-26.6893
ZINC00089932	-35.1137
ZINC00091744	-38.45
ZINC00094766	-30.4281
ZINC00097057	-35.8652
ZINC00099679	-32.9405
ZINC00100424	-41.0532
ZINC00101922	-39.2992
ZINC00106339	-34.992
ZINC00106339	-37.2116
ZINC00107648	-44.2946
ZINC00108853	-37.2552
ZINC00113377	-29.0993
ZINC00116637	-39.3323
ZINC00117177	-24.9898
ZINC00118143	-43.3649
ZINC00118217	-44.8451
ZINC00119806	-38.4778
ZINC00121572	-41.8709
ZINC00121609	-34.0103
ZINC00121719	-40.0467
ZINC00121932	-28.4458
ZINC00121932	-33.3728
ZINC00122457	-33.5152
ZINC00122457	-38.817
ZINC00123403	-26.6853
ZINC00125499	-23.9157
ZINC00126739	-46.3883
ZINC00129993	-39.6982
ZINC00130218	-41.3668
ZINC00131172	-37.4993
ZINC00133192	-31.9145
ZINC00136226	-33.8939
ZINC00136467	-39.9097
ZINC00137308	-40.8541
ZINC00138617	-33.5808
ZINC00138952	-42.1329
ZINC00140238	-30.9567
ZINC00142267	-29.17
ZINC00142897	-43.2738
ZINC00144912	-31.0657
ZINC00147978	-38.0907
ZINC00149389	-47.5128
ZINC00150280	-38.959

ZINC00152077	-44.6143
ZINC00152584	-31.7953
ZINC00152748	-33.6875
ZINC00157862	-26.182
ZINC00159222	-35.6348
ZINC00159296	-33.0968
ZINC00160771	-34.4616
ZINC00160907	-28.2789
ZINC00160956	-39.8948
ZINC00161461	-36.7126
ZINC00162517	-39.4127
ZINC00165143	-32.7829
ZINC00166492	-39.0763
ZINC00169269	-32.1259
ZINC00169407	-38.1702
ZINC00170315	-30.2935
ZINC00171717	-28.8171
ZINC00172191	-34.5529
ZINC00174403	-28.5743
ZINC00177204	-39.6403
ZINC00178637	-30.5259
ZINC00181988	-40.1091
ZINC00182406	-34.6364
ZINC00185245	-37.3492
ZINC00188097	-34.7771
ZINC00188443	-31.574
ZINC00190290	-37.3673
ZINC00201151	-35.5552
ZINC00201760	-33.9051
ZINC00204764	-36.5039
ZINC00207046	-35.1472
ZINC00216851	-36.6872
ZINC00226286	-40.021
ZINC00226395	-39.091
ZINC00233184	-41.9999
ZINC00234648	-33.4525
ZINC00235897	-26.2765
ZINC00238055	-26.2634
ZINC00239675	-34.2551
ZINC00240614	-36.9979
ZINC00241369	-32.9071
ZINC00241891	-26.3578
ZINC00245243	-32.326
ZINC00248726	-46.1775

ZINC00258082	-46.7783
ZINC00260955	-30.1667
ZINC00264796	-35.8008
ZINC00264796	-37.8357
ZINC00267172	-37.3859
ZINC00268925	-27.0258
ZINC00273637	-36.8872
ZINC00276501	-34.1573
ZINC00283212	-37.8165
ZINC00283212	-39.4228
ZINC00283212	-39.5727
ZINC00284400	-33.3228
ZINC00294969	-38.713
ZINC00298877	-44.5326
ZINC00302993	-32.7779
ZINC00303306	-41.5848
ZINC00304562	-33.2919
ZINC00307252	-34.1982
ZINC00308478	-40.5593
ZINC00308802	-39.5829
ZINC00309112	-39.3996
ZINC00313331	-39.0707
ZINC00314085	-34.8222
ZINC00315262	-46.8381
ZINC00319174	-27.8622
ZINC00334175	-30.7715
ZINC00334610	-33.367
ZINC00342018	-37.3899
ZINC00343409	-29.2677
ZINC00343409	-29.305
ZINC00345298	-32.1886
ZINC00346282	-30.7445
ZINC00351830	-43.1077
ZINC00358065	-41.6389
ZINC00358153	-42.2447
ZINC00360674	-34.6314
ZINC00363216	-33.8462
ZINC00366036	-32.0835
ZINC00369329	-37.5428
ZINC00375133	-35.8476
ZINC00377690	-27.7197
ZINC00377690	-27.8751
ZINC00384181	-38.0356
ZINC00385972	-45.4368

ZINC00393751	-36.7624
ZINC00396729	-31.4911
ZINC00398316	-29.5369
ZINC00399397	-35.7235
ZINC00399725	-37.1443
ZINC00406051	-32.7003
ZINC00407016	-32.2051
ZINC00411924	-23.0673
ZINC00418553	-38.9305
ZINC00424160	-44.7585
ZINC00428845	-39.8238
ZINC00428868	-36.6067
ZINC00431989	-40.7941
ZINC00432577	-22.315
ZINC00442081	-45.147
ZINC00442636	-36.5993
ZINC00442905	-33.7179
ZINC00444743	-34.7315
ZINC00446378	-45.5765
ZINC00450090	-39.5777
ZINC00450090	-45.0122
ZINC00451971	-43.9853
ZINC00452686	-37.3472
ZINC00454967	-31.8606
ZINC00455180	-43.2716
ZINC00458394	-33.086
ZINC00466477	-35.5261
ZINC00466589	-37.8381
ZINC00470992	-39.6429
ZINC00472790	-45.8054
ZINC00480505	-37.3972
ZINC00480898	-35.062
ZINC00482089	-24.6598
ZINC00489701	-31.1146
ZINC00496754	-45.5822
ZINC00502361	-33.7474
ZINC00504208	-44.5168
ZINC00511765	-36.5176
ZINC00512747	-38.8398
ZINC00513900	-39.1005
ZINC00519872	-35.9896
ZINC00525510	-33.2296
ZINC00529385	-33.9733
ZINC00529598	-34.6371

ZINC00535895	-32.9347
ZINC00536678	-40.5418
ZINC00540112	-31.8191
ZINC00540686	-33.0181
ZINC00544877	-41
ZINC00546282	-41.2319
ZINC00551104	-26.7502
ZINC00557400	-47.1351
ZINC00569097	-39.1169
ZINC00569287	-42.3846
ZINC00586924	-45.1106
ZINC00607738	-34.5614
ZINC00608460	-38.7209
ZINC00608539	-36.3714
ZINC00611221	-38.2032
ZINC00611492	-27.3121
ZINC00615708	-34.5229
ZINC00629159	-35.2339
ZINC00629159	-37.6913
ZINC00631983	-34.0995
ZINC00636874	-22.26
ZINC00638272	-41.1609
ZINC00647604	-36.2643
ZINC00649653	-20.3736
ZINC00658645	-44.7329
ZINC00658859	-23.6257
ZINC00661058	-28.8134
ZINC00672183	-43.1476
ZINC00672660	-22.0034
ZINC00679225	-35.0311
ZINC00679857	-37.2814
ZINC00679857	-41.3232
ZINC00685554	-45.9953
ZINC00702761	-35.0678
ZINC00702761	-37.158
ZINC00702985	-22.2509
ZINC00702985	-35.7341
ZINC00704786	-43.1977
ZINC00705601	-27.9828
ZINC00706420	-35.359
ZINC00706993	-37.7716
ZINC00707906	-32.36
ZINC00707998	-22.4167
ZINC00707998	-34.1437

ZINC00709616	-28.6625
ZINC00709760	-23.8978
ZINC00711123	-42.6037
ZINC00711510	-48.3684
ZINC00715098	-34.5998
ZINC00715098	-40.4499
ZINC00715191	-33.2358
ZINC00718787	-35.4191
ZINC00719751	-28.9011
ZINC00726049	-43.0663
ZINC00752396	-27.9707
ZINC00752673	-31.7585
ZINC00754071	-45.8701
ZINC00754499	-36.9304
ZINC00756387	-58.3891
ZINC00756481	-47.0712
ZINC00758785	-37.5238
ZINC00759378	-36.2584
ZINC00779527	-37.2026
ZINC00780017	-38.3826
ZINC00784737	-35.8334
ZINC00785610	-34.4867
ZINC00793636	-46.084
ZINC00793641	-44.1756
ZINC00794193	-38.5447
ZINC00800617	-42.897
ZINC00802087	-36.7144
ZINC00809035	-42.2378
ZINC00809713	-26.3752
ZINC00812893	-40.6281
ZINC00815409	-40.4755
ZINC00815942	-19.6974
ZINC00815942	-26.3772
ZINC00818052	-34.3609
ZINC00819837	-44.5194
ZINC00826077	-42.8138
ZINC00834057	-26.5417
ZINC00836693	-43.3815
ZINC00843126	-30.971
ZINC00846390	-40.0424
ZINC00852736	-41.3414
ZINC00858244	-23.2453
ZINC00858244	-30.9092
ZINC00859465	-27.7509

ZINC00860456	-39.6116
ZINC00865852	-37.0038
ZINC00866587	-34.0579
ZINC00871781	-37.2405
ZINC00874565	-34.2621
ZINC00875618	-20.5024
ZINC00885896	-38.2862
ZINC00889558	-31.766
ZINC00893020	-35.1261
ZINC00894522	-28.4706
ZINC00909935	-33.8135
ZINC00918515	-40.2404
ZINC00937251	-34.7757
ZINC00937251	-40.0123
ZINC00939449	-45.861
ZINC00940727	-36.256
ZINC00940727	-39.1134
ZINC00943956	-22.9496
ZINC00944363	-34.7556
ZINC00944363	-37.8014
ZINC00944607	-30.6203
ZINC00945436	-39.6245
ZINC00945516	-34.4212
ZINC00949672	-40.5148
ZINC00950491	-54.4086
ZINC00951503	-42.0278
ZINC00951867	-22.3493
ZINC00951867	-27.612
ZINC00959824	-38.8083
ZINC00966922	-48.7255
ZINC00967213	-33.6862
ZINC00967336	-25.8404
ZINC00968032	-29.1299
ZINC00972005	-43.1685
ZINC00972636	-46.9245
ZINC00973346	-37.4779
ZINC00981686	-31.7275
ZINC00987628	-39.131
ZINC00997049	-41.6353
ZINC00999306	-37.8102
ZINC01000817	-38.6373
ZINC01001991	-47.9622
ZINC01003509	-35.1391
ZINC01003709	-31.1202

ZINC01008543	-34.6515
ZINC01011102	-38.9275
ZINC01012108	-36.326
ZINC01012880	-44.5117
ZINC01022004	-44.671
ZINC01023577	-36.1013
ZINC01034980	-47.4871
ZINC01037256	-33.2341
ZINC01037256	-37.055
ZINC01037473	-34.5321
ZINC01039311	-33.731
ZINC01041514	-34.8571
ZINC01047716	-33.3086
ZINC01049464	-42.5345
ZINC01054598	-43.7234
ZINC01057347	-46.2236
ZINC01061696	-40.3592
ZINC01061696	-46.7612
ZINC01063124	-44.6247
ZINC01063358	-34.7368
ZINC01065159	-53.4003
ZINC01067092	-37.2411
ZINC01069376	-39.9867
ZINC01073872	-26.1511
ZINC01080325	-30.8919
ZINC01083216	-40.5406
ZINC01085047	-36.1079
ZINC01095042	-32.6765
ZINC01095485	-31.9207
ZINC01104661	-43.3038
ZINC01117624	-41.7342
ZINC01122386	-18.7157
ZINC01124475	-44.5208
ZINC01124682	-33.9521
ZINC01126956	-40.9767
ZINC01129399	-37.3401
ZINC01134862	-33.5705
ZINC01137253	-42.903
ZINC01137718	-42.7152
ZINC01148456	-42.7465
ZINC01155134	-45.9095
ZINC01155392	-36.4604
ZINC01163088	-37.6136
ZINC01167736	-50.5347

ZINC01172015	-41.5414
ZINC01175114	-43.3705
ZINC01175371	-32.7292
ZINC01176688	-24.1899
ZINC01177717	-24.1546
ZINC01181327	-40.6923
ZINC01184022	-41.5364
ZINC01185862	-27.5892
ZINC01186193	-28.658
ZINC01186817	-48.5056
ZINC01186979	-28.027
ZINC01191000	-39.2731
ZINC01198661	-37.533
ZINC01207046	-35.3307
ZINC01209276	-40.0051
ZINC01227048	-37.8013
ZINC01227048	-40.7475
ZINC01230112	-45.9302
ZINC01232441	-47.7543
ZINC01234699	-40.0356
ZINC01237813	-39.1805
ZINC01241575	-34.3555
ZINC01249916	-32.9129
ZINC01253459	-41.9648
ZINC01256160	-47.2588
ZINC01279942	-50.9094
ZINC01280966	-28.0916
ZINC01284498	-46.144
ZINC01297973	-43.2618
ZINC01298567	-37.0216
ZINC01302115	-44.8031
ZINC01309920	-33.9967
ZINC01318023	-38.2248
ZINC01322383	-48.2695
ZINC01337802	-41.0686
ZINC01339631	-41.7925
ZINC01339743	-22.0428
ZINC01371650	-35.5547
ZINC01372276	-46.6009
ZINC01378861	-39.7971
ZINC01382031	-31.5214
ZINC01383389	-32.3598
ZINC01383895	-37.5357
ZINC01384492	-34.6987

ZINC01386238	-33.2019
ZINC01387077	-38.1395
ZINC01387672	-29.9796
ZINC01387672	-29.9796
ZINC01390217	-46.9206
ZINC01393335	-31.4379
ZINC01393442	-42.6459
ZINC01397081	-36.2565
ZINC01400755	-40.733
ZINC01403813	-45.8505
ZINC01404298	-36.9761
ZINC01411060	-37.6309
ZINC01411712	-37.986
ZINC01413461	-34.3669
ZINC01414699	-30.2484
ZINC01414699	-36.174
ZINC01416041	-39.3443
ZINC01421129	-23.438
ZINC01422749	-26.5181
ZINC01423146	-44.3861
ZINC01423401	-37.8361
ZINC01424215	-44.5629
ZINC01424907	-47.197
ZINC01426207	-35.3521
ZINC01429624	-28.3425
ZINC01438470	-27.6922
ZINC01441224	-30.8225
ZINC01442729	-31.675
ZINC01450928	-42.2051
ZINC01453682	-34.1147
ZINC01453793	-26.7344
ZINC01461539	-21.9713
ZINC01466177	-42.9333
ZINC01466744	-41.7366
ZINC01498997	-38.5582
ZINC01503141	-35.5157
ZINC01504547	-35.2876
ZINC01504762	-34.486
ZINC01504994	-29.1293
ZINC01506896	-36.3709
ZINC01507107	-33.9876
ZINC01510436	-35.5631
ZINC01510536	-49.3463
ZINC01510814	-34.1936

ZINC01516227	-44.232
ZINC01527062	-29.5815
ZINC01542933	-47.8788
ZINC01542933	-51.7998
ZINC01559986	-34.2842
ZINC01560719	-36.2892
ZINC01580846	-26.8675
ZINC01584523	-25.6088
ZINC01590756	-31.1888
ZINC01591387	-32.9481
ZINC01592092	-35.4367
ZINC01593073	-37.1324
ZINC01598685	-34.2623
ZINC01600792	-29.0771
ZINC01608502	-38.1747
ZINC01612182	-34.2659
ZINC01616537	-37.0604
ZINC01619270	-43.3503
ZINC01625355	-39.5814
ZINC01628899	-39.4972
ZINC01629402	-30.6353
ZINC01665249	-32.185
ZINC01666940	-29.9675
ZINC01671447	-37.0825
ZINC01676371	-30.0826
ZINC01676678	-41.1539
ZINC01677185	-25.2799
ZINC01681286	-29.7412
ZINC01686454	-37.0386
ZINC01689598	-23.0562
ZINC01691068	-28.4941
ZINC01691268	-23.6017
ZINC01695178	-25.4102
ZINC01698058	-42.1737
ZINC01698412	-27.9286
ZINC01702395	-36.1354
ZINC01709355	-39.0487
ZINC01713662	-35.7001
ZINC01716666	-26.8715
ZINC01724835	-26.8774
ZINC01729149	-34.8923
ZINC01733994	-37.9496
ZINC01752741	-38.4464
ZINC01754675	-34.535

ZINC01757791	-52.618
ZINC01760278	-28.291
ZINC01766558	-27.878
ZINC01769127	-26.7869
ZINC01808204	-33.4432
ZINC01819785	-24.0916
ZINC01848447	-27.3114
ZINC01870905	-51.7768
ZINC01871095	-41.8023
ZINC01888987	-32.5355
ZINC01908604	-47.0237
ZINC01934830	-41.1914
ZINC01945538	-44.4789
ZINC01954687	-37.8185
ZINC01957738	-39.457
ZINC01958016	-44.8617
ZINC01958016	-48.4233
ZINC01958642	-31.1664
ZINC02004082	-32.5541
ZINC02006683	-33.0346
ZINC02017337	-32.2242
ZINC02023069	-37.8626
ZINC02026023	-40.6011
ZINC02050429	-31.9293
ZINC02057295	-40.2241
ZINC02066524	-33.0975
ZINC02071256	-39.6293
ZINC02075264	-37.8238
ZINC02077785	-27.672
ZINC02088226	-40.811
ZINC02095851	-40.9422
ZINC02104517	-41.2303
ZINC02106698	-34.1593
ZINC02121521	-47.3121
ZINC02129020	-42.409
ZINC02140305	-30.4134
ZINC02141082	-41.287
ZINC02159000	-44.9083
ZINC02163846	-38.1479
ZINC02166229	-49.6999
ZINC02167865	-38.5477
ZINC02169599	-27.4453
ZINC02169734	-28.0137
ZINC02171586	-40.2444

ZINC02173202	-36.181
ZINC02184354	-41.1915
ZINC02198926	-29.9477
ZINC02200308	-30.3591
ZINC02201186	-35.0765
ZINC02203848	-47.1134
ZINC02219710	-42.1342
ZINC02245908	-14.4035
ZINC02247503	-38.7234
ZINC02252669	-33.6218
ZINC02257295	-41.1812
ZINC02260243	-35.165
ZINC02260243	-42.1854
ZINC02260619	-24.2688
ZINC02271750	-33.1459
ZINC02272188	-44.1827
ZINC02288758	-35.3743
ZINC02289168	-38.0347
ZINC02297243	-34.1615
ZINC02304358	-29.2546
ZINC02304855	-30.4464
ZINC02306208	-43.4083
ZINC02309184	-43.538
ZINC02309350	-37.9546
ZINC02312740	-27.5045
ZINC02316538	-45.6416
ZINC02317230	-39.8155
ZINC02334363	-36.813
ZINC02334745	-29.9867
ZINC02357721	-45.5524
ZINC02359613	-40.2205
ZINC02359704	-37.412
ZINC02360611	-34.7001
ZINC02364159	-27.1759
ZINC02367932	-42.7745
ZINC02369241	-36.6971
ZINC02384115	-24.8176
ZINC02387828	-34.9073
ZINC02391801	-47.4563
ZINC02396923	-42.6186
ZINC02396923	-45.0862
ZINC02397450	-39.3667
ZINC02397587	-37.6629
ZINC02397787	-30.3682

ZINC02408820	-36.6091
ZINC02408820	-38.2566
ZINC02411850	-33.3973
ZINC02425726	-40.3292
ZINC02433746	-28.2021
ZINC02435635	-33.5903
ZINC02435637	-30.7117
ZINC02438523	-35.8706
ZINC02452225	-22.1307
ZINC02452914	-41.7203
ZINC02454596	-40.0576
ZINC02463309	-31.5979
ZINC02464582	-36.1995
ZINC02466542	-26.1315
ZINC02466563	-30.5495
ZINC02467739	-30.2098
ZINC02468580	-25.7831
ZINC02473958	-28.6277
ZINC02480065	-18.1431
ZINC02481755	-29.749
ZINC02483136	-44.306
ZINC02484245	-30.0507
ZINC02489976	-33.5268
ZINC02500793	-28.116
ZINC02502660	-43.6759
ZINC02508142	-29.3088
ZINC02508142	-29.3504
ZINC02508486	-36.2232
ZINC02521384	-41.1782
ZINC02526853	-31.6011
ZINC02531058	-44.1917
ZINC02538021	-40.6942
ZINC02546429	-37.7058
ZINC02548243	-34.3717
ZINC02557861	-35.0614
ZINC02566482	-29.5487
ZINC02570906	-40.2383
ZINC02570996	-38.2588
ZINC02576670	-36.3144
ZINC02581863	-44.9899
ZINC02586221	-37.5593
ZINC02593941	-32.8996
ZINC02601181	-30.4705
ZINC02622181	-30.9212

ZINC02626384	-40.9277
ZINC02628378	-47.1477
ZINC02640191	-39.0609
ZINC02640191	-47.8131
ZINC02642567	-28.8234
ZINC02643050	-36.9074
ZINC02659969	-30.7154
ZINC02666969	-29.8867
ZINC02686400	-30.9287
ZINC02704300	-44.6901
ZINC02709006	-36.8778
ZINC02709261	-43.7505
ZINC02719228	-24.355
ZINC02719228	-30.6888
ZINC02719228	-34.693
ZINC02730637	-34.5403
ZINC02737465	-47.1811
ZINC02738003	-50.0889
ZINC02741107	-37.9948
ZINC02743243	-33.4789
ZINC02744481	-34.4331
ZINC02756010	-46.8932
ZINC02757031	-27.481
ZINC02758681	-39.5898
ZINC02764583	-37.3162
ZINC02765009	-35.6754
ZINC02766640	-40.2725
ZINC02770132	-30.0045
ZINC02776171	-34.675
ZINC02777816	-42.2229
ZINC02778159	-37.3135
ZINC02778238	-49.9379
ZINC02779190	-46.734
ZINC02787929	-33.9888
ZINC02792807	-39.9369
ZINC02794471	-34.0288
ZINC02794714	-48.6833
ZINC02795372	-51.0022
ZINC02797945	-49.1329
ZINC02828199	-44.7387
ZINC02837885	-45.7516
ZINC02850194	-43.0889
ZINC02852285	-43.8027
ZINC02852644	-41.6636

ZINC02852717	-46.2954
ZINC02866608	-36.6965
ZINC02872565	-39.6074
ZINC02872692	-44.1321
ZINC02873928	-39.6117
ZINC02874606	-41.1987
ZINC02877558	-44.848
ZINC02879092	-35.4923
ZINC02879092	-36.9593
ZINC02881736	-43.1448
ZINC02884938	-44.7654
ZINC02890322	-33.6488
ZINC02891894	-31.9137
ZINC02892927	-45.244
ZINC02902659	-36.0776
ZINC02911491	-38.8843
ZINC02911507	-34.1342
ZINC02913128	-38.582
ZINC02914549	-34.9465
ZINC02916227	-41.0386
ZINC02919744	-25.0058
ZINC02919744	-38.0576
ZINC02925795	-39.4281
ZINC02927333	-37.8737
ZINC02932968	-39.3724
ZINC02934416	-50.5322
ZINC02944456	-23.2142
ZINC02947983	-33.3209
ZINC02953586	-53.3448
ZINC02975992	-44.3751
ZINC02975992	-50.546
ZINC02979681	-31.9606
ZINC02981439	-34.4627
ZINC02982588	-38.5786
ZINC02990268	-39.0685
ZINC02991174	-33.0575
ZINC02999792	-34.3286
ZINC03006242	-38.2799
ZINC03016867	-46.1132
ZINC03027636	-34.4489
ZINC03029348	-36.4098
ZINC03029622	-36.5286
ZINC03043685	-40.7025
ZINC03043685	-42.3913

ZINC03068044	-44.1698
ZINC03068439	-31.8477
ZINC03070627	-38.2877
ZINC03073046	-44.4027
ZINC03084551	-48.5244
ZINC03089798	-40.9383
ZINC03091632	-36.0753
ZINC03096165	-36.7556
ZINC03121363	-41.4408
ZINC03121962	-42.2202
ZINC03122928	-35.0258
ZINC03130674	-33.4405
ZINC03133618	-35.4311
ZINC03135219	-43.123
ZINC03137939	-38.2893
ZINC03142788	-36.9501
ZINC03149500	-33.092
ZINC03150035	-39.4907
ZINC03150693	-42.821
ZINC03154282	-44.0038
ZINC03159303	-36.4056
ZINC03162857	-30.5144
ZINC03164422	-24.4076
ZINC03164895	-32.5512
ZINC03165579	-25.4828
ZINC03166258	-36.3148
ZINC03167350	-40.2438
ZINC03171111	-33.9871
ZINC03174999	-38.3729
ZINC03180170	-28.7372
ZINC03181301	-45.4808
ZINC03194801	-42.2486
ZINC03195576	-31.5847
ZINC03223720	-33.3761
ZINC03228758	-37.4885
ZINC03231992	-53.928
ZINC03238881	-28.3198
ZINC03241872	-43.9622
ZINC03242427	-28.4667
ZINC03244254	-36.8236
ZINC03248143	-52.6777
ZINC03272159	-46.3648
ZINC03275258	-35.0471
ZINC03276236	-40.5524

ZINC03277836	-42.1514
ZINC03279981	-38.5082
ZINC03302300	-39.1283
ZINC03316045	-32.473
ZINC03317807	-49.2672
ZINC03321151	-40.8559
ZINC03331148	-35.6577
ZINC03331531	-43.0196
ZINC03333382	-36.6294
ZINC03334345	-37.6427
ZINC03343796	-40.4337
ZINC03347441	-36.4462
ZINC03353371	-35.4187
ZINC03355366	-52.6036
ZINC03359723	-42.1046
ZINC03369303	-29.9451
ZINC03374104	-44.7863
ZINC03394045	-33.343
ZINC03403087	-44.493
ZINC03415425	-33.9113
ZINC03431000	-33.3763
ZINC03461890	-39.4258
ZINC03463450	-42.5287
ZINC03464164	-41.0974
ZINC03465787	-36.6708
ZINC03478785	-44.1616
ZINC03479820	-34.6917
ZINC03503941	-37.1741
ZINC03505140	-46.0297
ZINC03533907	-26.9022
ZINC03542416	-39.543
ZINC03545203	-38.9927
ZINC03549235	-44.867
ZINC03549890	-43.1781
ZINC03555319	-34.5548
ZINC03562880	-38.3561
ZINC03566573	-36.6396
ZINC03570269	-42.5557
ZINC03570269	-47.0004
ZINC03572565	-42.6312
ZINC03591042	-45.0667
ZINC03617143	-45.9591
ZINC03621486	-49.5382
ZINC03622749	-31.574

ZINC03631503	-31.5022
ZINC03636421	-32.7175
ZINC03646523	-24.5727
ZINC03671727	-44.5602
ZINC03682860	-31.7494
ZINC03732371	-27.0784
ZINC03831238	-33.6065
ZINC03832937	-38.8447
ZINC03833057	-38.3421
ZINC03838956	-52.8316
ZINC03841443	-34.2923
ZINC03841476	-33.3616
ZINC03845553	-32.4044
ZINC03847171	-31.6474
ZINC03849107	-44.9106
ZINC03849349	-32.9318
ZINC03850851	-36.1545
ZINC03850973	-32.176
ZINC03868856	-40.4625
ZINC03871859	-35.9427
ZINC03873990	-32.9827
ZINC03875357	-28.3486
ZINC03883856	-34.2297
ZINC03884039	-37.4126
ZINC03886166	-32.9869
ZINC03888342	-43.3758
ZINC03888385	-42.2457
ZINC03888456	-31.2818
ZINC03896836	-43.1524
ZINC03896836	-44.2764
ZINC03897280	-40.2935
ZINC03898289	-42.6051
ZINC03902923	-47.9812
ZINC03906266	-40.0766
ZINC03911119	-49.8407
ZINC03911126	-44.6428
ZINC03911159	-36.6936
ZINC03911947	-44.8958
ZINC03912733	-36.707
ZINC03912733	-39.0031
ZINC03913813	-46.292
ZINC03977921	-34.1154
ZINC03978192	-24.8409
ZINC03978455	-39.5222

ZINC03996385	-41.2007
ZINC03998673	-37.2705
ZINC03999935	-38.7738
ZINC04000434	-37.9085
ZINC04012523	-29.3648
ZINC04013628	-35.6868
ZINC04015200	-45.3991
ZINC04015976	-36.8749
ZINC04027458	-25
ZINC04043390	-29.9518
ZINC04046438	-44.0475
ZINC04054419	-37.2644
ZINC04058221	-41.7507
ZINC04064583	-38.2403
ZINC04065461	-44.8288
ZINC04073346	-40.9508
ZINC04078438	-34.0859
ZINC04078832	-41.5805
ZINC04081854	-27.4993
ZINC04082387	-23.989
ZINC04086411	-43.5874
ZINC04094466	-35.3464
ZINC04095269	-39.1802
ZINC04095269	-48.3775
ZINC04095358	-41.5289
ZINC04095388	-31.6131
ZINC04095388	-38.6323
ZINC04099272	-34.3928
ZINC04107063	-36.0916
ZINC04120200	-41.7629
ZINC04122361	-39.7418
ZINC04123021	-43.6617
ZINC04125019	-42.4257
ZINC04125028	-41.033
ZINC04125112	-26.3567
ZINC04127211	-24.3769
ZINC04139963	-26.6307
ZINC04146940	-24.1102
ZINC04151414	-28.0725
ZINC04154660	-24.0355
ZINC04154660	-26.9129
ZINC04157288	-42.561
ZINC04158516	-47.2939
ZINC04159037	-37.0903

ZINC04159133	-48.592
ZINC04165282	-26.5952
ZINC04167418	-42.0044
ZINC04167418	-43.4154
ZINC04169875	-41.4516
ZINC04172026	-28.0765
ZINC04172270	-30.3415
ZINC04173468	-27.6225
ZINC04183962	-40.9359
ZINC04184281	-28.2194
ZINC04188721	-41.6877
ZINC04192772	-32.6235
ZINC04200428	-36.9321
ZINC04201777	-36.0842
ZINC04206375	-36.2137
ZINC04206661	-41.3287
ZINC04206968	-41.8979
ZINC04207823	-32.0824
ZINC04215173	-41.9186
ZINC04216775	-34.4418
ZINC04218683	-34.7994
ZINC04228036	-24.2998
ZINC04233563	-40.0678
ZINC04233940	-40.5996
ZINC04236184	-36.3885
ZINC04237659	-37.6122
ZINC04249555	-42.1094
ZINC04253138	-33.6065
ZINC04255654	-25.401
ZINC04258186	-45.411
ZINC04263107	-42.3109
ZINC04263965	-43.174
ZINC04264755	-36.1248
ZINC04267587	-28.7484
ZINC04269055	-52.2582
ZINC04278160	-41.4671
ZINC04281003	-40.7582
ZINC04284509	-44.8589
ZINC04290573	-26.8025
ZINC04299525	-47.7025
ZINC04306182	-39.6465
ZINC04311027	-32.8736
ZINC04312120	-38.6502
ZINC04315865	-29.7387

ZINC04316286	-29.0576
ZINC04322967	-39.2686
ZINC04323948	-45.4156
ZINC04325218	-42.1165
ZINC04326730	-45.3075
ZINC04327837	-50.1564
ZINC04327985	-17.266
ZINC04327985	-25.684
ZINC04333136	-40.0381
ZINC04334957	-32.7992
ZINC04339583	-36.4348
ZINC04343684	-43.0357
ZINC04344411	-36.6732
ZINC04347724	-40.2361
ZINC04347724	-45.317
ZINC04347962	-42.2816
ZINC04352517	-40.1003
ZINC04352629	-42.8068
ZINC04356863	-50.0724
ZINC04358144	-38.3245
ZINC04358210	-40.3471
ZINC04359708	-34.9695
ZINC04359708	-35.2111
ZINC04359708	-41.8275
ZINC04359708	-42.2553
ZINC04361514	-26.0906
ZINC04370879	-42.7291
ZINC04373750	-42.0843
ZINC04387389	-26.2177
ZINC04412609	-36.1838
ZINC04414653	-30.9675
ZINC04416656	-41.7941
ZINC04438680	-35.7574
ZINC04450963	-39.0597
ZINC04473365	-33.7361
ZINC04474253	-27.7595
ZINC04477286	-36.8499
ZINC04477797	-44.9909
ZINC04477967	-39.9595
ZINC04481396	-40.1157
ZINC04482076	-44.3733
ZINC04483717	-41.9405
ZINC04483717	-46.8095
ZINC04484093	-52.9342

ZINC04487917	-45.6892
ZINC04488739	-40.1799
ZINC04494355	-29.2066
ZINC04494355	-34.1072
ZINC04494355	-38.8636
ZINC04496193	-37.5475
ZINC04496246	-43.8281
ZINC04501626	-44.1934
ZINC04501626	-48.5204
ZINC04503538	-50.8796
ZINC04508190	-44.3525
ZINC04510682	-36.9752
ZINC04510928	-43.034
ZINC04511168	-36.0135
ZINC04514024	-42.7061
ZINC04514315	-45.4003
ZINC04517837	-48.2696
ZINC04521614	-29.0108
ZINC04524557	-43.8166
ZINC04525648	-40.1976
ZINC04525648	-42.8197
ZINC04530683	-22.3098
ZINC04535157	-32.0296
ZINC04536174	-37.4631
ZINC04545174	-39.37
ZINC04547584	-35.0871
ZINC04552476	-24.0871
ZINC04552476	-33.0293
ZINC04577745	-24.0471
ZINC04578168	-34.7158
ZINC04580283	-36.0456
ZINC04589224	-38.8544
ZINC04589458	-40.9218
ZINC04590114	-38.7933
ZINC04592485	-20.9797
ZINC04596620	-37.9816
ZINC04611339	-40.1948
ZINC04614669	-37.3435
ZINC04614774	-44.4704
ZINC04618978	-32.5619
ZINC04632383	-39.5896
ZINC04638321	-22.3962
ZINC04638321	-26.1683
ZINC04644238	-30.6459

ZINC04653509	-24.9514
ZINC04656222	-39.4731
ZINC04660973	-34.1529
ZINC04660973	-39.9231
ZINC04663296	-37.184
ZINC04672720	-47.1216
ZINC04672852	-41.9338
ZINC04678865	-37.6161
ZINC04678865	-38.9225
ZINC04680387	-38.6535
ZINC04682193	-37.5779
ZINC04684539	-38.0339
ZINC04685232	-47.7564
ZINC04686011	-37.7754
ZINC04689777	-45.3859
ZINC04690232	-43.9481
ZINC04694373	-30.7791
ZINC04695198	-37.3532
ZINC04696878	-44.4598
ZINC04697168	-40.5228
ZINC04697169	-28.3529
ZINC04698907	-38.7125
ZINC04701476	-44.5474
ZINC04702170	-27.5659
ZINC04703545	-53.8526
ZINC04704085	-40.7095
ZINC04705336	-32.1872
ZINC04705336	-40.0841
ZINC04705352	-45.0435
ZINC04711834	-33.0556
ZINC04712513	-45.2357
ZINC04713069	-32.6624
ZINC04715522	-35.954
ZINC04715794	-32.5575
ZINC04716141	-27.9083
ZINC04716141	-33.8446
ZINC04716141	-35.9023
ZINC04718298	-37.6332
ZINC04721515	-35.3803
ZINC04724645	-37.8585
ZINC04725627	-33.4815
ZINC04725997	-34.6862
ZINC04725997	-35.4483
ZINC04725997	-36.3263

ZINC04730757	-28.8717
ZINC04730757	-30.3089
ZINC04733854	-45.0031
ZINC04737962	-46.0313
ZINC04737985	-32.6541
ZINC04738055	-41.2757
ZINC04738596	-46.2612
ZINC04738718	-49.367
ZINC04740055	-45.68
ZINC04742909	-26.0487
ZINC04743947	-51.0517
ZINC04744362	-45.8105
ZINC04746156	-35.3858
ZINC04751307	-32.4729
ZINC04762525	-42.437
ZINC04764709	-32.7108
ZINC04767607	-27.4885
ZINC04770238	-47.3541
ZINC04775566	-34.2651
ZINC04776233	-34.7239
ZINC04785237	-37.1908
ZINC04785237	-46.6428
ZINC04785485	-43.5068
ZINC04785529	-36.8308
ZINC04789853	-35.3567
ZINC04794500	-30.7759
ZINC04800835	-38.0342
ZINC04802945	-22.784
ZINC04803160	-21.8608
ZINC04803160	-32.0051
ZINC04804124	-46.7986
ZINC04804475	-39.1655
ZINC04808940	-32.9577
ZINC04809784	-26.375
ZINC04809784	-28.2418
ZINC04810489	-34.3451
ZINC04813539	-31.1873
ZINC04821557	-32.6824
ZINC04829831	-45.8628
ZINC04829848	-27.5942
ZINC04842307	-40.3119
ZINC04843384	-30.4782
ZINC04843980	-44.0935
ZINC04844466	-38.6836

ZINC04844469	-40.3412
ZINC04845138	-45.0095
ZINC04853551	-38.678
ZINC04854871	-33.1091
ZINC04858002	-37.9667
ZINC04859723	-45.0612
ZINC04860659	-37.6066
ZINC04863489	-38.543
ZINC04866569	-40.7713
ZINC04868875	-26.1963
ZINC04871619	-30.0235
ZINC04903953	-39.5499
ZINC04904461	-45.8208
ZINC04915070	-33.4377
ZINC04915539	-25.294
ZINC04915653	-27.7038
ZINC04919668	-39.948
ZINC04923428	-33.2242
ZINC04933949	-35.0845
ZINC04934708	-42.8655
ZINC04939046	-41.4873
ZINC04940096	-37.9816
ZINC04940142	-38.4837
ZINC04942222	-39.9599
ZINC04942964	-37.7662
ZINC04948267	-25.7111
ZINC04949359	-54.1262
ZINC04952451	-37.8595
ZINC04955818	-43.7011
ZINC04957346	-38.4107
ZINC04963818	-35.8784
ZINC04964557	-48.3676
ZINC04965030	-40.8243
ZINC04968335	-31.542
ZINC04968378	-21.4681
ZINC04969787	-31.2504
ZINC04970618	-37.9947
ZINC04973596	-29.7661
ZINC04975191	-33.6909
ZINC04977437	-36.8981
ZINC04977475	-38.0811
ZINC04978238	-40.0879
ZINC04979559	-39.0301
ZINC04980897	-36.7661

ZINC04981109	-36.2838
ZINC04981166	-49.3793
ZINC04988818	-32.0727
ZINC04989077	-35.6985
ZINC04991802	-41.0582
ZINC04994896	-44.1605
ZINC04996175	-30.8837
ZINC04997631	-36.0422
ZINC04998309	-42.7194
ZINC05000337	-39.3217
ZINC05002246	-34.0716
ZINC05006086	-24.3627
ZINC05008572	-39.299
ZINC05020364	-34.2114
ZINC05020364	-34.3017
ZINC05022388	-42.6666
ZINC05027038	-35.2384
ZINC05027740	-33.0545
ZINC05030904	-28.034
ZINC05034722	-40.4255
ZINC05041012	-38.5733
ZINC05042498	-32.9303
ZINC05042697	-38.9711
ZINC05045705	-40.587
ZINC05047091	-36.5956
ZINC05055814	-38.6339
ZINC05055969	-31.4474
ZINC05061377	-46.6925
ZINC05070527	-40.768
ZINC05078230	-38.0136
ZINC05080674	-37.3661
ZINC05080702	-36.1254
ZINC05091909	-37.427
ZINC05095408	-29.2987
ZINC05100648	-40.4488
ZINC05111498	-35.8392
ZINC05122686	-46.1021
ZINC05123364	-54.1358
ZINC05123931	-48.2896
ZINC05125267	-38.0858
ZINC05127139	-38.2238
ZINC05129356	-31.0997
ZINC05130240	-22.2624
ZINC05142013	-35.2656

ZINC05144838	-43.1012
ZINC05154524	-43.4886
ZINC05156269	-39.2125
ZINC05156415	-30.2279
ZINC05167702	-48.976
ZINC05171886	-38.768
ZINC05174773	-39.799
ZINC05179313	-45.9478
ZINC05179700	-38.1068
ZINC05186322	-36.8928
ZINC05189661	-50.2552
ZINC05190429	-31.2552
ZINC05191348	-37.8044
ZINC05192841	-33.6847
ZINC05198010	-34.8888
ZINC05199418	-34.9084
ZINC05199849	-27.1463
ZINC05199849	-27.7103
ZINC05203636	-33.3352
ZINC05211401	-33.1117
ZINC05211711	-34.2233
ZINC05211910	-32.4956
ZINC05222921	-41.9381
ZINC05225154	-38.4982
ZINC05225298	-38.9277
ZINC05226853	-40.3544
ZINC05234221	-41.199
ZINC05237849	-41.8775
ZINC05237849	-48.4493
ZINC05240605	-28.3929
ZINC05240897	-34.5045
ZINC05241436	-50.7744
ZINC05245435	-39.0647
ZINC05248309	-34.5722
ZINC05248613	-31.3331
ZINC05253025	-36.1277
ZINC05269850	-35.0422
ZINC05276520	-33.6979
ZINC05279547	-37.4587
ZINC05283078	-32.616
ZINC05284997	-33.5269
ZINC05286113	-25.9406
ZINC05289406	-34.9263
ZINC05291760	-41.7978

ZINC05293471	-39.7492
ZINC05293471	-41.3794
ZINC05296809	-37.5819
ZINC05296809	-41.5527
ZINC05299341	-44.1356
ZINC05306721	-32.3395
ZINC05318087	-29.5949
ZINC05319204	-35.4846
ZINC05337536	-42.005
ZINC05338054	-32.7411
ZINC05343181	-26.1879
ZINC05347537	-37.1612
ZINC05351546	-26.9495
ZINC05351546	-27.676
ZINC05351791	-30.9951
ZINC05359394	-37.0249
ZINC05371069	-40.5827
ZINC05371213	-36.3168
ZINC05371590	-31.9231
ZINC05371988	-44.9546
ZINC05376664	-42.3226
ZINC05377128	-33.2756
ZINC05377635	-38.897
ZINC05380861	-39.8582
ZINC05380874	-35.3469
ZINC05382686	-29.5501
ZINC05382784	-30.4959
ZINC05382784	-34.574
ZINC05382784	-37.1876
ZINC05385438	-27.9891
ZINC05385438	-27.9891
ZINC05385438	-35.1183
ZINC05387413	-32.6732
ZINC05390969	-35.2981
ZINC05396121	-46.8614
ZINC05396214	-39.6756
ZINC05397191	-40.0829
ZINC05399887	-37.3254
ZINC05399887	-37.4114
ZINC05401434	-35.5876
ZINC05408551	-47.3383
ZINC05408751	-33.1712
ZINC05415320	-34.8934
ZINC05417151	-28.6788

ZINC05421045	-37.4567
ZINC05427175	-38.3415
ZINC05428676	-39.0146
ZINC05438219	-32.74
ZINC05440811	-34.6751
ZINC05445456	-23.6529
ZINC05445499	-32.1378
ZINC05445499	-45.2002
ZINC05446127	-37.7757
ZINC05461161	-38.7016
ZINC05461161	-42.036
ZINC05461939	-35.0757
ZINC05470890	-35.1311
ZINC05477995	-30.3805
ZINC05478153	-46.4111
ZINC05478326	-40.7196
ZINC05484000	-31.0709
ZINC05484000	-31.4423
ZINC05490062	-32.4882
ZINC05490442	-26.8014
ZINC05490664	-31.1258
ZINC05499876	-41.4291
ZINC05503790	-40.6567
ZINC05504034	-36.2946
ZINC05504034	-45.1373
ZINC05506138	-44.7179
ZINC05514932	-42.6264
ZINC05515886	-38.6732
ZINC05516049	-32.1767
ZINC05516502	-30.1789
ZINC05517465	-37.9366
ZINC05521512	-29.3107
ZINC05526054	-34.6856
ZINC05527019	-34.5367
ZINC05527827	-34.7498
ZINC05541837	-28.5228
ZINC05545465	-42.4453
ZINC05545465	-42.9688
ZINC05545465	-50.8767
ZINC05546124	-30.6551
ZINC05550596	-32.9436
ZINC05557925	-36.2126
ZINC05559597	-39.4631
ZINC05576333	-48.0607

ZINC05578071	-42.1272
ZINC05578133	-43.4769
ZINC05578619	-31.2724
ZINC05583092	-27.861
ZINC05584552	-36.0866
ZINC05589896	-31.4428
ZINC05595918	-31.4155
ZINC05595918	-35.7588
ZINC05596041	-29.0964
ZINC05596041	-35.6242
ZINC05600304	-40.3631
ZINC05602880	-46.9648
ZINC05603204	-40.896
ZINC05604306	-31.5221
ZINC05604609	-34.7055
ZINC05604711	-33.3659
ZINC05613949	-30.3701
ZINC05619944	-43.5345
ZINC05627055	-45.7602
ZINC05627353	-36.6465
ZINC05638430	-31.5415
ZINC05638430	-33.5049
ZINC05659858	-39.1076
ZINC05667451	-41.9032
ZINC05673158	-38.3003
ZINC05674952	-38.6955
ZINC05674981	-38.8786
ZINC05675129	-33.537
ZINC05677073	-31.5328
ZINC05677587	-37.2945
ZINC05680994	-33.6294
ZINC05682969	-49.4502
ZINC05685213	-36.8521
ZINC05685917	-37.8946
ZINC05685917	-44.1193
ZINC05687130	-37.8312
ZINC05699437	-39.4057
ZINC05703390	-45.9866
ZINC05717325	-43.4536
ZINC05729826	-33.2045
ZINC05734375	-40.2498
ZINC05739447	-36.1121
ZINC05739825	-30.2388
ZINC05740471	-39.2592

ZINC05741693	-28.8607
ZINC05753695	-37.9207
ZINC05767689	-39.5541
ZINC05769174	-43.1268
ZINC05769759	-43.7828
ZINC05776151	-49.0956
ZINC05781225	-25.5454
ZINC05785177	-28.1593
ZINC05793397	-39.9357
ZINC05793398	-31.6628
ZINC05793398	-31.6628
ZINC05793563	-44.4709
ZINC05798582	-36.907
ZINC05798723	-34.9414
ZINC05813261	-28.0574
ZINC05813261	-28.1946
ZINC05829297	-43.3522
ZINC05835383	-32.2408
ZINC05837030	-35.749
ZINC05838198	-37.4134
ZINC05843511	-45.2092
ZINC05854274	-30.0822
ZINC05855383	-31.8323
ZINC05863997	-35.2741
ZINC05882709	-30.1745
ZINC05884068	-28.2057
ZINC05884068	-32.5395
ZINC05886785	-40.274
ZINC05887336	-37.1153
ZINC05895328	-42.3396
ZINC05898668	-40.7969
ZINC05899449	-35.4218
ZINC05906601	-27.179
ZINC05907983	-36.2331
ZINC05907983	-37.4725
ZINC05909089	-26.1577
ZINC05921232	-33.8037
ZINC05921568	-37.0272
ZINC05936369	-44.8557
ZINC05936493	-42.1598
ZINC05944258	-37.95
ZINC05967203	-31.7677
ZINC05976469	-25.81
ZINC05997244	-41.6435

ZINC06005321	-29.3474
ZINC06005321	-32.7809
ZINC06024394	-42.1105
ZINC06024394	-53.5004
ZINC06048799	-38.7848
ZINC06051217	-38.9096
ZINC06059651	-38.8544
ZINC06059948	-42.4583
ZINC06066054	-40.0501
ZINC06073994	-44.9949
ZINC06085651	-43.2271
ZINC06090272	-34.5546
ZINC06143896	-41.7014
ZINC06154325	-41.1353
ZINC06158519	-39.0705
ZINC06159108	-36.4313
ZINC06176752	-33.2071
ZINC06181670	-46.5442
ZINC06183929	-27.7168
ZINC06187452	-32.3079
ZINC06187452	-33.5244
ZINC06188226	-39.0288
ZINC06191794	-51.4603
ZINC06195819	-36.7715
ZINC06195819	-38.0859
ZINC06211469	-37.6411
ZINC06214626	-40.8006
ZINC06221640	-40.3463
ZINC06222696	-34.5505
ZINC06228100	-43.1123
ZINC06229395	-37.158
ZINC06230618	-39.8309
ZINC06238547	-37.2141
ZINC06238829	-26.8939
ZINC06238829	-33.7721
ZINC06238829	-39.2936
ZINC06240019	-40.8012
ZINC06242377	-27.9159
ZINC06243401	-43.4927
ZINC06248799	-19.3074
ZINC06258350	-50.708
ZINC06267411	-29.4404
ZINC06269125	-50.6044
ZINC06276842	-37.1159

ZINC06279368	-35.9019
ZINC06282835	-43.9442
ZINC06314808	-39.5099
ZINC06314834	-32.9889
ZINC06326155	-46.69
ZINC06328842	-49.827
ZINC06330558	-44.084
ZINC06331924	-41.2055
ZINC06334169	-42.0575
ZINC06337736	-44.9274
ZINC06337808	-28.2671
ZINC06342715	-38.756
ZINC06381872	-42.919
ZINC06382439	-30.1304
ZINC06382439	-40.4079
ZINC06383110	-45.9252
ZINC06394406	-35.1748
ZINC06398751	-42.5158
ZINC06399836	-41.9912
ZINC06401508	-33.1106
ZINC06406697	-19.9926
ZINC06420371	-31.8009
ZINC06423978	-21.8907
ZINC06442596	-33.6647
ZINC06445610	-29.3027
ZINC06445610	-35.7273
ZINC06468091	-33.4363
ZINC06468091	-43.7372
ZINC06468200	-35.9259
ZINC06479984	-39.4994
ZINC06492639	-41.2526
ZINC06493767	-38.0733
ZINC06510554	-25.2763
ZINC06514147	-36.0061
ZINC06526976	-37.3141
ZINC06527999	-32.9634
ZINC06532382	-33.0728
ZINC06547195	-39.1269
ZINC06553818	-27.8773
ZINC06557394	-33.8587
ZINC06570064	-31.274
ZINC06588894	-40.0287
ZINC06614150	-38.0099
ZINC06625478	-40.5066

ZINC06633873	-40.4278
ZINC06640691	-38.8781
ZINC06650996	-35.5407
ZINC06657315	-38.1216
ZINC06659067	-43.0772
ZINC06662613	-37.4405
ZINC06664716	-36.238
ZINC06667693	-32.2039
ZINC06668516	-29.4843
ZINC06668516	-30.5362
ZINC06679196	-29.4428
ZINC06682128	-35.2698
ZINC06684013	-35.1243
ZINC06700928	-42.2638
ZINC06714395	-37.9567
ZINC06720064	-33.3184
ZINC06720064	-35.7471
ZINC06724157	-29.6392
ZINC06729239	-37.1314
ZINC06731937	-27.8018
ZINC06733045	-39.7526
ZINC06736926	-42.2422
ZINC06737038	-34.5665
ZINC06737848	-37.4899
ZINC06744711	-40.0715
ZINC06748342	-33.7142
ZINC06748414	-41.347
ZINC06752718	-43.3516
ZINC06756478	-34.29
ZINC06756582	-39.9336
ZINC06756582	-41.4372
ZINC06767235	-31.7685
ZINC06776117	-40.1853
ZINC06786117	-29.7586
ZINC06786117	-31.1027
ZINC06786362	-33.4342
ZINC06787652	-27.7489
ZINC06790386	-46.2416
ZINC06790623	-38.5151
ZINC06790633	-45.3231
ZINC06796393	-39.8744
ZINC06802518	-34.5436
ZINC06809804	-30.1298
ZINC06817442	-37.9976

ZINC06823386	-35.8828
ZINC06825200	-39.6435
ZINC06829201	-34.5616
ZINC06832180	-34.8079
ZINC06841154	-39.604
ZINC06842982	-39.4641
ZINC06849419	-36.434
ZINC06849547	-45.3348
ZINC06850625	-38.3846
ZINC06850784	-36.6309
ZINC06859652	-36.9941
ZINC06864593	-38.2275
ZINC06866687	-46.9262
ZINC06877634	-33.5533
ZINC06879783	-44.5939
ZINC06880126	-37.5495
ZINC06881386	-43.1501
ZINC06891670	-45.3092
ZINC06895305	-38.9266
ZINC06900928	-36.7513
ZINC06903318	-45.8286
ZINC06908366	-36.5599
ZINC06908366	-37.6606
ZINC06910866	-40.5909
ZINC06914810	-44.4735
ZINC06921321	-50.0355
ZINC06922229	-38.7563
ZINC06925357	-39.6393
ZINC06926820	-37.4365
ZINC06942632	-48.9562
ZINC06944093	-36.3751
ZINC06944574	-36.9939
ZINC06947616	-38.3571
ZINC06948589	-42.1237
ZINC06963959	-34.9175
ZINC06983015	-38.8223
ZINC06989522	-40.0521
ZINC07023291	-34.7568
ZINC07028165	-45.345
ZINC07056042	-44.2709
ZINC07059210	-32.9454
ZINC07092445	-46.28
ZINC07124118	-48.735
ZINC07137559	-42.1249

ZINC07139201	-36.5701
ZINC07144115	-32.8762
ZINC07150049	-50.3512
ZINC07161667	-51.2134
ZINC07161960	-47.1998
ZINC07169020	-43.428
ZINC07185995	-39.1759
ZINC07191917	-37.0882
ZINC07193883	-39.7933
ZINC07194399	-37.8897
ZINC07194841	-43.8752
ZINC07236541	-45.7877
ZINC07236671	-44.8886
ZINC07257434	-36.9962
ZINC07274758	-35.9237
ZINC07318674	-46.4366
ZINC07329146	-38.9989
ZINC07336048	-45.8021
ZINC07340180	-33.5441
ZINC07341507	-32.8227
ZINC07343133	-46.1505
ZINC07343133	-49.3092
ZINC07347387	-48.6191
ZINC07366464	-44.2485
ZINC07377507	-29.4897
ZINC07384835	-33.0931
ZINC07392407	-37.2549
ZINC07396949	-41.5707
ZINC07400464	-44.2499
ZINC07402259	-40.5201
ZINC07450070	-37.3745
ZINC07454401	-22.2093
ZINC07497902	-37.7008
ZINC07519880	-34.2869
ZINC07529762	-32.5196
ZINC07553158	-33.0218
ZINC07558626	-29.8589
ZINC07586595	-46.5722
ZINC07587794	-40.0498
ZINC07594128	-33.0508
ZINC07600902	-42.3033
ZINC07609761	-39.0061
ZINC07619677	-31.1778
ZINC07622914	-36.0444

ZINC07676053	-42.3856
ZINC07676053	-43.2873
ZINC07706639	-37.7033
ZINC07742016	-40.0595
ZINC07752197	-35.7728
ZINC07756036	-37.3504
ZINC07756949	-36.6971
ZINC07756949	-42.2671
ZINC07777397	-40.6471
ZINC07779127	-41.5643
ZINC07779332	-44.933
ZINC07779393	-38.5375
ZINC07779646	-41.6749
ZINC07784224	-45.4204
ZINC07786015	-44.6066
ZINC07787552	-34.8349
ZINC07805298	-37.0622
ZINC07811990	-36.3186
ZINC07814183	-38.3147
ZINC07852692	-35.2255
ZINC07882770	-43.5982
ZINC07883232	-40.944
ZINC07898964	-38.3761
ZINC07911637	-41.5464
ZINC07912003	-49.8621
ZINC07916480	-37.8245
ZINC07945623	-45.9032
ZINC07945623	-47.9156
ZINC07960656	-33.5886
ZINC07962000	-35.7644
ZINC07983583	-43.0518
ZINC07985852	-48.5739
ZINC07985897	-36.3293
ZINC07996520	-33.0939
ZINC08014302	-46.1228
ZINC08022333	-39.1605
ZINC08023504	-32.8206
ZINC08024689	-34.1475
ZINC08028282	-46.8021
ZINC08028490	-41.9848
ZINC08033983	-41.1368
ZINC08038421	-37.6632
ZINC08047054	-38.8219
ZINC08053558	-34.961

ZINC08062657	-45.7918
ZINC08105877	-48.5263
ZINC08109851	-34.6363
ZINC08109851	-46.2825
ZINC08112101	-33.2429
ZINC08133921	-34.9636
ZINC08152283	-46.9083
ZINC08152283	-49.3017
ZINC08175003	-46.294
ZINC08179255	-45.6926
ZINC08184046	-35.9274
ZINC08184219	-44.6218
ZINC08186477	-44.1368
ZINC08186828	-35.528
ZINC08189832	-40.6354
ZINC08190336	-47.9533
ZINC08203469	-35.0131
ZINC08216759	-41.0919
ZINC08232632	-45.9069
ZINC08274767	-45.0579
ZINC08276946	-36.3768
ZINC08277010	-30.2513
ZINC08295748	-42.9684
ZINC08296263	-36.6885
ZINC08299650	-35.1659
ZINC08312530	-51.738
ZINC08317645	-39.3869
ZINC08322906	-47.9216
ZINC08327214	-44.4668
ZINC08345819	-40.1026
ZINC08359850	-32.9635
ZINC08376823	-43.1494
ZINC08388682	-33.438
ZINC08389196	-40.106
ZINC08399763	-30.8958
ZINC08400213	-40.7287
ZINC08401993	-50.8088
ZINC08408522	-34.7529
ZINC08410168	-33.0381
ZINC08419573	-39.4874
ZINC08427796	-37.2762
ZINC08429437	-44.0389
ZINC08432294	-10.6897
ZINC08432294	-16.6216

ZINC08435970	-37.0382
ZINC08441023	-31.4048
ZINC08447348	-36.9011
ZINC08451220	-32.6571
ZINC08453225	-31.1519
ZINC08470446	-45.6269
ZINC08486625	-42.5557
ZINC08513603	-43.7134
ZINC08528836	-44.2986
ZINC08553319	-35.5754
ZINC08559217	-36.2466
ZINC08559237	-30.762
ZINC08559938	-39.9225
ZINC08560107	-36.3635
ZINC08564938	-47.4184
ZINC08565092	-46.5178
ZINC08567334	-39.3561
ZINC08588554	-25.475
ZINC08589182	-34.0085
ZINC08595113	-30.2846
ZINC08600680	-33.9521
ZINC08611138	-38.5193
ZINC08615688	-33.3966
ZINC08617648	-31.2053
ZINC08620248	-39.0737
ZINC08648557	-34.5052
ZINC08656933	-33.7043
ZINC08671881	-36.051
ZINC08673311	-30.7127
ZINC08685246	-32.5009
ZINC08687450	-37.4581
ZINC08694977	-34.8224
ZINC08694977	-40.7688
ZINC08695098	-33.8049
ZINC08695098	-36.0901
ZINC08698080	-25.6304
ZINC08698254	-22.9288
ZINC08699106	-36.022
ZINC08707956	-40.2406
ZINC08709304	-34.4335
ZINC08719809	-44.222
ZINC08726307	-35.2544
ZINC08729711	-34.9674
ZINC08739963	-43.0688

ZINC08740213	-27.9615
ZINC08742753	-38.0246
ZINC08742753	-38.9368
ZINC08742871	-27.9439
ZINC08746375	-33.527
ZINC08746375	-40.2879
ZINC08749564	-42.3974
ZINC08750066	-44.8791
ZINC08756346	-40.1325
ZINC08757032	-45.4446
ZINC08757145	-34.3658
ZINC08761877	-38.008
ZINC08762557	-43.5825
ZINC08767969	-42.9977
ZINC08768326	-32.4128
ZINC08768546	-33.3913
ZINC08768559	-36.1766
ZINC08769153	-32.8936
ZINC08780700	-41.2387
ZINC08792479	-41.1562
ZINC08815844	-39.6032
ZINC08820330	-48.4789
ZINC08821479	-57.0859
ZINC08838110	-44.1346
ZINC08838854	-44.6849
ZINC08844371	-29.3852
ZINC08845150	-29.0431
ZINC08845150	-36.4649
ZINC08854984	-36.0569
ZINC08856321	-27.5417
ZINC08856321	-28.8347
ZINC08871838	-33.2013
ZINC08877973	-41.2491
ZINC08880029	-40.3159
ZINC08910445	-29.3106
ZINC08910863	-43.4657
ZINC08914358	-40.8938
ZINC08914822	-40.4693
ZINC08916237	-32.8242
ZINC08919016	-28.9628
ZINC08930366	-36.6306
ZINC08931886	-31.804
ZINC08934964	-32.9254
ZINC08935756	-38.7492

ZINC08943754	-45.1293
ZINC08970057	-42.8905
ZINC08970883	-44.5264
ZINC08972304	-30.2896
ZINC08972304	-36.539
ZINC08974420	-40.6052
ZINC08974420	-43.1799
ZINC08984747	-45.7324
ZINC08990848	-37.162
ZINC08990883	-21.2286
ZINC08991225	-52.1047
ZINC08991636	-46.3303
ZINC08996196	-19.9039
ZINC08996196	-20.9625
ZINC08997284	-34.6631
ZINC08997284	-45.5372
ZINC09005178	-34.8518
ZINC09009183	-26.2972
ZINC09009183	-28.0633
ZINC09010819	-43.2965
ZINC09012084	-32.2732
ZINC09012084	-35.8123
ZINC09012461	-36.3968
ZINC09012461	-40.5684
ZINC09012461	-44.0952
ZINC09012461	-45.4825
ZINC09012565	-36.3992
ZINC09013612	-20.0031
ZINC09015294	-26.9677
ZINC09015915	-33.0981
ZINC09015915	-37.5214
ZINC09028293	-36.1847
ZINC09029168	-40.3091
ZINC09036590	-43.173
ZINC09036701	-36.9677
ZINC09036745	-39.8356
ZINC09041244	-23.3751
ZINC09042977	-39.9698
ZINC09046326	-26.9507
ZINC09046326	-35.5518
ZINC09049457	-51.9519
ZINC09054217	-45.3619
ZINC09058864	-30.455
ZINC09065577	-45.8509

ZINC09072859	-43.8729
ZINC09073141	-21.1912
ZINC09087695	-34.8592
ZINC09088112	-40.1247
ZINC09088112	-40.2536
ZINC09088112	-42.9666
ZINC09088176	-41.8679
ZINC09096139	-40.5005
ZINC09110304	-42.4917
ZINC09111441	-29.0503
ZINC09111476	-28.4842
ZINC09114959	-35.8117
ZINC09116777	-43.5304
ZINC09123632	-33.1092
ZINC09123737	-31.1361
ZINC09124849	-31.5494
ZINC09135657	-53.1329
ZINC09140511	-42.0852
ZINC09140721	-49.1524
ZINC09143660	-42.8495
ZINC09155131	-33.4446
ZINC09155513	-27.2807
ZINC09158213	-35.5206
ZINC09160334	-27.4907
ZINC09160589	-35.3885
ZINC09160589	-35.6651
ZINC09164739	-43.0479
ZINC09193048	-43.3583
ZINC09199145	-53.9849
ZINC09203130	-42.0443
ZINC09204740	-44.8495
ZINC09206464	-32.5586
ZINC09210753	-14.2651
ZINC09216004	-41.341
ZINC09223209	-32.4586
ZINC09226478	-34.779
ZINC09227009	-34.431
ZINC09229406	-47.6733
ZINC09239670	-30.5302
ZINC09242308	-29.6736
ZINC09242389	-33.3321
ZINC09242546	-34.2301
ZINC09242546	-35.4268
ZINC09244267	-21.4259

ZINC09244267	-21.7182
ZINC09244789	-47.3647
ZINC09249771	-38.8494
ZINC09258956	-35.5351
ZINC09264510	-43.3814
ZINC09270813	-36.9168
ZINC09271129	-35.2623
ZINC09271129	-39.2229
ZINC09271978	-30.0127
ZINC09274626	-35.9174
ZINC09282603	-36.5507
ZINC09287111	-43.3625
ZINC09287725	-39.0717
ZINC09288337	-43.7134
ZINC09293255	-22.8284
ZINC09294313	-36.4697
ZINC09305250	-35.9335
ZINC09305907	-48.7599
ZINC09309136	-37.6915
ZINC09312377	-28.2253
ZINC09312377	-33.5288
ZINC09316992	-43.8881
ZINC09322167	-44.9231
ZINC09327424	-24.3916
ZINC09327424	-37.1374
ZINC09327424	-38.261
ZINC09327424	-39.3381
ZINC09327424	-39.3914
ZINC09328440	-31.9217
ZINC09329414	-44.6393
ZINC09330030	-24.6747
ZINC09346649	-46.8184
ZINC09348622	-38.8423
ZINC09353135	-31.3998
ZINC09354497	-22.7608
ZINC09354497	-34.8672
ZINC09354497	-48.3601
ZINC09356832	-34.5502
ZINC09359541	-39.6734
ZINC09374065	-34.36
ZINC09374065	-35.0412
ZINC09374065	-40.1537
ZINC09374065	-41.7841
ZINC09374173	-24.7479

ZINC09374173	-38.4173
ZINC09378395	-37.2325
ZINC09378395	-41.9982
ZINC09378487	-35.772
ZINC09380496	-19.6576
ZINC09380496	-30.6148
ZINC09380496	-35.4683
ZINC09388073	-33.1038
ZINC09401902	-47.0996
ZINC09406226	-39.489
ZINC09407642	-26.9648
ZINC09410753	-27.2464
ZINC09413509	-29.5294
ZINC09419790	-36.4768
ZINC09425097	-38.316
ZINC09434348	-49.7474
ZINC09434772	-37.0798
ZINC09437316	-36.4267
ZINC09437316	-37.2344
ZINC09439954	-46.4973
ZINC09441035	-50.9667
ZINC09447857	-36.9221
ZINC09461518	-29.2566
ZINC09461780	-28.2921
ZINC09461780	-32.7319
ZINC09461780	-37.9677
ZINC09463108	-33.6719
ZINC09463108	-35.5528
ZINC09464052	-46.8436
ZINC09467347	-38.6276
ZINC09470921	-33.7698
ZINC09478520	-45.6774
ZINC09481788	-55.2752
ZINC09485634	-38.8486
ZINC09486573	-44.2637
ZINC09490617	-27.4556
ZINC09492105	-28.2791
ZINC09497321	-25.4111
ZINC09497321	-39.7253
ZINC09498980	-29.0568
ZINC09512646	-39.956
ZINC09517094	-33.3142
ZINC09517571	-29.617
ZINC09518356	-46.4429

ZINC09529739	-29.6796
ZINC09543414	-37.6665
ZINC09547320	-38.4299
ZINC09548678	-43.0496
ZINC09557127	-26.9055
ZINC09557127	-30.7296
ZINC09557514	-23.731
ZINC09557514	-36.3601
ZINC09581691	-26.8376
ZINC09586378	-43.3932
ZINC09589051	-41.4103
ZINC09601711	-43.7578
ZINC09602371	-37.3706
ZINC09621781	-38.7485
ZINC09631855	-24.4534
ZINC09634457	-35.9242
ZINC09634831	-42.7667
ZINC09641351	-28.4146
ZINC09641708	-48.0823
ZINC09666227	-37.921
ZINC09667246	-40.7573
ZINC09668916	-35.6114
ZINC09670117	-49.3747
ZINC09675194	-37.1912
ZINC09675194	-48.7011
ZINC09695503	-27.404
ZINC09704767	-48.5305
ZINC09720948	-49.3859
ZINC09725914	-34.1785
ZINC09730707	-28.8242
ZINC09730707	-30.7591
ZINC09742186	-40.7034
ZINC09750243	-42.8
ZINC09821471	-38.516
ZINC09834431	-31.9178
ZINC09865824	-30.1939
ZINC09882785	-46.6955
ZINC09910325	-49.6112
ZINC09915762	-25.5325
ZINC09927385	-41.8096
ZINC09935109	-28.3165
ZINC09935109	-35.4686
ZINC09935319	-32.2012
ZINC09936449	-35.4409

ZINC09942102	-49.9795
ZINC09949283	-34.2086
ZINC09952931	-39.886
ZINC09961477	-26.2651
ZINC09969141	-40.6698
ZINC09970507	-27.6505
ZINC09972489	-28.6226
ZINC10010570	-44.676
ZINC10012881	-42.2118
ZINC10015143	-31.5264
ZINC10025186	-43.3845
ZINC10028811	-30.9826
ZINC10033419	-37.4311
ZINC10046397	-39.1757
ZINC10075288	-44.2944
ZINC10084446	-46.1516
ZINC10109778	-46.0895
ZINC10128089	-40.9272
ZINC10142162	-30.0942
ZINC10149311	-52.3033
ZINC10156736	-39.6598
ZINC10157223	-30.8454
ZINC10157925	-33.8893
ZINC10203587	-45.3467
ZINC10214250	-28.9569
ZINC10214559	-32.034
ZINC10215526	-45.7182
ZINC10219721	-28.5726
ZINC10220677	-42.4335
ZINC10225145	-40.0862
ZINC10225145	-41.8223
ZINC10232576	-27.5281
ZINC10232576	-29.5239
ZINC10252931	-34.6135
ZINC10255312	-40.4925
ZINC10286781	-37.0623
ZINC10325267	-43.4579
ZINC10339295	-29.6772
ZINC10339374	-41.4753
ZINC10431761	-31.6376
ZINC10463105	-46.5683
ZINC10472094	-34.4592
ZINC10611804	-42.29
ZINC10643055	-41.9266

ZINC10688778	-37.098
ZINC10691072	-47.8926
ZINC10699430	-31.0353
ZINC10742803	-45.3461
ZINC10749741	-56.5946
ZINC10756312	-44.2855
ZINC10810774	-42.4243
ZINC10815974	-44.7623
ZINC10844565	-43.2143
ZINC10846171	-46.6967
ZINC10900727	-40.0599
ZINC10905060	-46.1954
ZINC11004274	-47.5134
ZINC11004658	-23.7762
ZINC11071704	-49.7721
ZINC11086262	-34.0748
ZINC11088152	-28.6476
ZINC11157318	-30.5209
ZINC11173771	-41.1251
ZINC11247022	-46.3917
ZINC11285318	-48.0556
ZINC11322841	-46.5441
ZINC11327125	-36.5085
ZINC11380126	-39.1741
ZINC11389667	-34.3005
ZINC11443185	-37.6486
ZINC11520623	-34.3333
ZINC11526394	-38.4869
ZINC11535718	-26.969
ZINC11536060	-26.386
ZINC11536060	-28.229
ZINC11536060	-34.0473
ZINC11536283	-28.6334
ZINC11544278	-39.3612
ZINC11567865	-36.6387
ZINC11568569	-39.1287
ZINC11613894	-40.9196
ZINC11615807	-44.2204
ZINC11661377	-28.2561
ZINC11666261	-34.782
ZINC11667134	-32.7033
ZINC11690300	-36.4518
ZINC11691444	-42.5403
ZINC11695836	-46.0346

ZINC11716025	-40.043
ZINC11736746	-30.1614
ZINC11740080	-41.6053
ZINC11758293	-31.3839
ZINC11763440	-45.9848
ZINC11781283	-31.4495
ZINC11782091	-40.4534
ZINC11788827	-35.1306
ZINC11790229	-29.5546
ZINC11802989	-26.3576
ZINC11814693	-43.1584
ZINC11814697	-33.5607
ZINC11817195	-33.6284
ZINC11819682	-41.4277
ZINC11819682	-49.4733
ZINC11821798	-22.2333
ZINC11822143	-29.779
ZINC11822143	-36.8346
ZINC11841486	-37.1764
ZINC11842665	-23.1568
ZINC11842665	-40.7836
ZINC11846106	-39.0362
ZINC11849504	-36.581
ZINC11853773	-40.1274
ZINC11865194	-32.336
ZINC11866088	-39.6563
ZINC11878616	-27.5708
ZINC11878942	-35.4822
ZINC11879448	-33.204
ZINC11880264	-45.9777
ZINC11888191	-37.2705
ZINC11900343	-33.48
ZINC11900664	-50.5557
ZINC11907383	-45.2809
ZINC11907571	-40.9651
ZINC11907860	-40.6319
ZINC11916442	-34.8849
ZINC11917651	-39.7758
ZINC11932744	-43.18
ZINC11933641	-33.8412
ZINC11935049	-46.8816
ZINC11936971	-36.8631
ZINC11937636	-31.163
ZINC11957070	-36.1316

ZINC11977431	-41.4679
ZINC11998171	-36.0421
ZINC12001767	-44.3617
ZINC12002060	-29.4783
ZINC12016188	-32.7223
ZINC12028194	-41.4186
ZINC12029380	-45.1508
ZINC12033635	-39.5245
ZINC12038095	-23.9537
ZINC12053763	-38.2257
ZINC12062795	-37.3706
ZINC12072162	-47.223
ZINC12080520	-42.0533
ZINC12086194	-30.7037
ZINC12087001	-34.1239
ZINC12087653	-35.0435
ZINC12087653	-43.1496
ZINC12092985	-29.9263
ZINC12113298	-42.8037
ZINC12113779	-40.3417
ZINC12113835	-35.2741
ZINC12113835	-38.9235
ZINC12117168	-34.2142
ZINC12117727	-33.482
ZINC12119207	-39.8858
ZINC12121546	-52.9992
ZINC12121799	-27.4876
ZINC12134000	-30.7796
ZINC12139030	-39.9183
ZINC12139584	-28.642
ZINC12139612	-37.7962
ZINC12144485	-30.7805
ZINC12167537	-35.1033
ZINC12175905	-34.082
ZINC12176702	-37.1895
ZINC12176702	-46.918
ZINC12182906	-51.1629
ZINC12183637	-44.5651
ZINC12183740	-29.24
ZINC12184001	-37.8318
ZINC12184113	-38.9434
ZINC12184697	-38.4347
ZINC12184697	-39.027
ZINC12185316	-35.3397

ZINC12191536	-39.98
ZINC12204025	-33.2244
ZINC12207600	-29.9567
ZINC12218912	-37.2645
ZINC12222016	-46.9654
ZINC12222929	-27.483
ZINC12223317	-35.927
ZINC12236770	-38.0187
ZINC12241785	-35.0823
ZINC12243149	-31.8816
ZINC12249732	-35.2698
ZINC12256809	-35.0014
ZINC12273954	-40.0122
ZINC12275901	-41.0481
ZINC12280074	-48.3704
ZINC12288064	-44.1388
ZINC12302301	-44.3182
ZINC12302301	-46.2747
ZINC12304419	-45.4899
ZINC12306240	-52.6148
ZINC12308175	-31.3039
ZINC12308292	-42.1373
ZINC12309009	-26.8012
ZINC12314748	-36.7845
ZINC12315150	-44.9513
ZINC12318430	-43.5675
ZINC12321858	-28.9579
ZINC12323791	-39.1233
ZINC12323791	-44.1162
ZINC12324978	-40.2577
ZINC12326334	-37.7684
ZINC12329629	-32.8259
ZINC12329999	-36.0799
ZINC12331121	-28.7493
ZINC12334225	-36.9103
ZINC12334225	-41.6075
ZINC12337203	-32.2987
ZINC12337386	-49.2066
ZINC12338037	-36.4744
ZINC12338253	-41.8241
ZINC12339957	-43.7006
ZINC12341384	-26.2155
ZINC12344049	-39.2866
ZINC12345767	-38.244

ZINC12346428	-42.7661
ZINC12347665	-33.3971
ZINC12352243	-35.3155
ZINC12361064	-38.3342
ZINC12363690	-27.9474
ZINC12364018	-27.8885
ZINC12364207	-40.2014
ZINC12367334	-29.8647
ZINC12368172	-33.7897
ZINC12368508	-47.9441
ZINC12368704	-43.4264
ZINC12369295	-46.9202
ZINC12370207	-33.7301
ZINC12370568	-25.4506
ZINC12376099	-47.8968
ZINC12383925	-36.9141
ZINC12387665	-29.7238
ZINC12394735	-43.5445
ZINC12396592	-37.4163
ZINC12402523	-29.6323
ZINC12403731	-38.5873
ZINC12409952	-44.461
ZINC12410298	-40.1549
ZINC12411988	-44.9163
ZINC12412896	-39.4714
ZINC12413729	-35.302
ZINC12417488	-38.8034
ZINC12417501	-38.9487
ZINC12417869	-38.7706
ZINC12418444	-26.6889
ZINC12418445	-35.5594
ZINC12419347	-30.7835
ZINC12419801	-25.7187
ZINC12420806	-41.5767
ZINC12429904	-38.4266
ZINC12442577	-30.2597
ZINC12447835	-32.7395
ZINC12453195	-39.7036
ZINC12456691	-47.5304
ZINC12456786	-43.4019
ZINC12462517	-42.3305
ZINC12464100	-28.9506
ZINC12468305	-43.8233
ZINC12468966	-39.331

ZINC12469205	-36.269
ZINC12483857	-42.5958
ZINC12485929	-35.4858
ZINC12495694	-28.9356
ZINC12497262	-33.4954
ZINC12513610	-37.9243
ZINC12516695	-29.8781
ZINC12517199	-35.3894
ZINC12520147	-41.9952
ZINC12520147	-43.0165
ZINC12521802	-20.2988
ZINC12526097	-42.4219
ZINC12528253	-39.7219
ZINC12530642	-29.9976
ZINC12535388	-47.9752
ZINC12535388	-49.7365
ZINC12537022	-30.357
ZINC12540719	-37.0098
ZINC12541397	-34.3257
ZINC12541397	-44.9451
ZINC12543323	-40.6902
ZINC12543559	-39.3852
ZINC12543750	-35.799
ZINC12547943	-44.4375
ZINC12548840	-44.4246
ZINC12551250	-36.1279
ZINC12553143	-37.2954
ZINC12553230	-40.2274
ZINC12557856	-30.6994
ZINC12561465	-40.4891
ZINC12562226	-47.2721
ZINC12563221	-39.9168
ZINC12566057	-39.8474
ZINC12566741	-52.7854
ZINC12569218	-32.0996
ZINC12572139	-26.8367
ZINC12575450	-32.0992
ZINC12580345	-21.9991
ZINC12580345	-42.8368
ZINC12592542	-37.448
ZINC12593778	-39.1286
ZINC12598759	-33.1034
ZINC12599819	-44.5002
ZINC12601140	-35.0049

ZINC12602525	-34.6151
ZINC12615591	-41.0148
ZINC12616465	-39.4469
ZINC12622489	-34.6015
ZINC12623488	-45.8271
ZINC12627972	-43.0518
ZINC12644349	-47.37
ZINC12644432	-31.2886
ZINC12658261	-44.0973
ZINC12664197	-38.229
ZINC12682618	-29.6788
ZINC12682618	-38.1494
ZINC12683124	-34.0395
ZINC12685661	-44.6401
ZINC12690907	-38.9436
ZINC12710542	-41.1487
ZINC12733339	-40.1274
ZINC12733339	-50.8132
ZINC12734207	-30.7481
ZINC12747139	-26.8031
ZINC12750450	-52.6575
ZINC12755380	-40.0765
ZINC12756061	-38.4423
ZINC12756409	-36.2008
ZINC12758186	-30.3803
ZINC12763552	-37.884
ZINC12763979	-38.4671
ZINC12771868	-41.4661
ZINC12774285	-39.2072
ZINC12774534	-48.1977
ZINC12774730	-45.7129
ZINC12781209	-43.9004
ZINC12782539	-37.5801
ZINC12800394	-39.4604
ZINC12805474	-34.8057
ZINC12807921	-32.6735
ZINC12814966	-31.5058
ZINC12815324	-37.298
ZINC12816101	-28.3322
ZINC12816101	-34.3806
ZINC12816338	-41.7487
ZINC12817606	-41.9725
ZINC12817857	-40.1805
ZINC12855001	-43.5667

ZINC12856216	-37.9896
ZINC12858034	-41.5217
ZINC12877276	-30.6351
ZINC12879538	-36.9137
ZINC12880723	-49.8036
ZINC12884281	-49.4823
ZINC12887522	-46.4003
ZINC12909700	-35.7706
ZINC12912084	-38.9116
ZINC12912283	-28.8778
ZINC12912950	-41.0126
ZINC12913008	-33.0517
ZINC12916743	-44.8198
ZINC12917484	-25.9482
ZINC12919114	-35.6296
ZINC12925763	-40.3154
ZINC12930015	-49.6516
ZINC12932066	-46.2952
ZINC12932913	-38.229
ZINC12932913	-47.8803
ZINC12933582	-32.7118
ZINC12933582	-37.8877
ZINC12935885	-35.5296
ZINC12943288	-33.2692
ZINC12947470	-49.6137
ZINC12949311	-33.5972
ZINC12949800	-46.0738
ZINC12956522	-27.4847
ZINC12977907	-43.7627
ZINC12980319	-48.956
ZINC12981608	-40.8995
ZINC12981757	-42.8051
ZINC12985182	-41.5245
ZINC12985432	-37.7153
ZINC12986413	-36.6039
ZINC12986658	-42.1178
ZINC12986709	-42.1221
ZINC12986966	-40.3141
ZINC12988087	-28.4395
ZINC12997569	-37.6511
ZINC12998361	-43.5921
ZINC13004939	-32.6653
ZINC13004939	-35.8805
ZINC13004939	-40.438

ZINC13029459	-30.8417
ZINC13036395	-32.681
ZINC13038679	-28.4945
ZINC13038679	-43.0759
ZINC13058204	-43.5359
ZINC13061821	-55.8663
ZINC13068520	-40.0688
ZINC13069605	-39.1092
ZINC13074917	-49.7121
ZINC13076644	-39.3999
ZINC13092156	-51.4353
ZINC13092445	-53.9301
ZINC13094910	-45.3396
ZINC13096032	-38.5668
ZINC13096442	-40.1415
ZINC13099276	-39.5422
ZINC13100765	-29.6855
ZINC13112974	-42.3736
ZINC13118893	-22.0579
ZINC13119497	-38.8109
ZINC13119538	-46.5041
ZINC13120677	-40.6861
ZINC13120677	-52.5647
ZINC13121823	-37.5065
ZINC13122216	-38.9866
ZINC13122263	-37.8767
ZINC13122263	-40.6117
ZINC13122263	-41.0058
ZINC13122343	-32.2988
ZINC13124474	-38.6946
ZINC13125573	-36.183
ZINC13126978	-28.4855
ZINC13126978	-29.2477
ZINC13129218	-31.7951
ZINC13134513	-49.7196
ZINC13134513	-51.3061
ZINC13141169	-51.6288
ZINC13141675	-31.4141
ZINC13141675	-37.4044
ZINC13153139	-32.2568
ZINC13153854	-31.6694
ZINC13154246	-46.1376
ZINC13154912	-37.8467
ZINC13154912	-38.1622

ZINC13161713	-23.5791
ZINC13161713	-28.3073
ZINC13161713	-32.1849
ZINC13207124	-35.2218
ZINC13208208	-31.09
ZINC13213746	-40.7005
ZINC13213783	-37.2615
ZINC13248968	-47.7823
ZINC13260735	-44.4026
ZINC13282445	-28.583
ZINC13285785	-56.0582
ZINC13305955	-49.3101
ZINC13320997	-42.9773
ZINC13321222	-47.2903
ZINC13331762	-31.7416
ZINC13395229	-44.8438
ZINC13403895	-27.1722
ZINC13407427	-33.7102
ZINC13424651	-20.973
ZINC13466938	-29.6341
ZINC13467220	-23.6383
ZINC13471082	-40.4926
ZINC13476624	-43.3855
ZINC13476624	-43.9353
ZINC13482521	-29.4119
ZINC13496078	-27.6165
ZINC13496731	-35.0452
ZINC13496731	-37.0737
ZINC13497293	-47.7753
ZINC13503815	-29.0287
ZINC13504962	-32.5829
ZINC13511310	-30.3263
ZINC13511448	-34.5655
ZINC13512068	-30.1333
ZINC13512242	-35.0605
ZINC13512376	-23.5451
ZINC13512508	-31.2524
ZINC13539894	-44.0813
ZINC13546440	-30.6528
ZINC13549095	-39.7148
ZINC13549142	-40.0564
ZINC13551488	-36.5904
ZINC13561612	-33.7058
ZINC13567806	-30.502

ZINC13568712	-42.3737
ZINC13568712	-55.4362
ZINC13569927	-32.5431
ZINC13569927	-36.5612
ZINC13571455	-30.7278
ZINC13571455	-31.8528
ZINC13573443	-37.7994
ZINC13574891	-43.1468
ZINC13591052	-31.0028
ZINC13606862	-29.558
ZINC13617265	-35.7746
ZINC13617265	-38.6394
ZINC13624960	-40.5075
ZINC13626258	-41.3266
ZINC13628979	-50.5563
ZINC13629369	-50.5997
ZINC13629661	-42.1815
ZINC13629981	-23.6804
ZINC13638122	-48.4278
ZINC13640103	-29.1156
ZINC13640405	-33.1947
ZINC13641677	-32.6682
ZINC13642049	-35.4088
ZINC13653871	-21.354
ZINC13669148	-31.2211
ZINC13669743	-38.2115
ZINC13671004	-34.6865
ZINC13688566	-33.151
ZINC13688801	-47.4949
ZINC13688954	-28.1516
ZINC13689518	-43.5172
ZINC13689667	-39.483
ZINC13689802	-31.0643
ZINC13689802	-36.7085
ZINC13690346	-31.1933
ZINC13690346	-33.9925
ZINC13692437	-43.3522
ZINC13698199	-40.8495
ZINC13720674	-37.0884
ZINC13722232	-29.3184
ZINC13722232	-44.8609
ZINC13722557	-30.9471
ZINC13722557	-31.507
ZINC13728484	-52.8042

ZINC13729393	-29.3066
ZINC13732684	-30.8421
ZINC13732684	-35.6829
ZINC13736292	-34.7107
ZINC13736743	-37.633
ZINC13747777	-38.2172
ZINC13757679	-31.1636
ZINC13757679	-40.9241
ZINC13757679	-42.0469
ZINC13757679	-42.1179
ZINC13757679	-44.9877
ZINC13758705	-28.2064
ZINC13768404	-38.5929
ZINC13784479	-44.3556
ZINC13789402	-38.8018
ZINC13789458	-44.358
ZINC13790199	-33.1557
ZINC13790199	-38.0549
ZINC13804934	-47.3372
ZINC13805582	-39.4247
ZINC13810589	-31.9762
ZINC13810790	-39.0793
ZINC13840372	-36.5023
ZINC13856968	-31.8568
ZINC13881073	-36.5068
ZINC13881311	-38.1774
ZINC13893466	-42.1429
ZINC13894668	-51.9838
ZINC13941188	-34.3858
ZINC13943258	-51.1127
ZINC13957003	-25.5399
ZINC13960276	-44.4334
ZINC13973445	-34.2535
ZINC13986081	-39.0632
ZINC13988515	-39.25
ZINC14000255	-39.193
ZINC14000255	-43.2878
ZINC14000490	-43.6084
ZINC14002728	-37.2322
ZINC14020083	-41.8281
ZINC14020849	-38.4478
ZINC14021842	-42.0806
ZINC14038673	-46.8177
ZINC14050094	-47.0821

ZINC14059141	-34.9122
ZINC14064691	-51.0679
ZINC14086188	-39.3192
ZINC14093783	-23.3369
ZINC14116446	-37.9316
ZINC14121191	-54.6107
ZINC14137538	-38.5099
ZINC14162293	-16.1989
ZINC14162357	-44.6198
ZINC14181170	-41.9865
ZINC14185239	-32.7737
ZINC14185468	-37.2237
ZINC14187150	-28.6214
ZINC14187472	-39.5536
ZINC14187472	-41.2094
ZINC14187826	-49.271
ZINC14196711	-34.6019
ZINC14201516	-36.3082
ZINC14215233	-24.3588
ZINC14215233	-30.9468
ZINC14220162	-41.548
ZINC14233941	-41.6441
ZINC14241021	-32.4311
ZINC14247780	-33.5242
ZINC14254316	-26.4884
ZINC14257517	-47.7148
ZINC14258308	-41.5928
ZINC14270749	-40.4522
ZINC14284795	-47.2289
ZINC14310267	-31.1129
ZINC14310267	-41.4077
ZINC14366259	-30.6345
ZINC14383228	-29.4001
ZINC14455812	-35.4481
ZINC14456370	-36.6213
ZINC14456867	-40.7112
ZINC14457479	-43.6138
ZINC14458976	-31.6231
ZINC14458982	-33.4647
ZINC14459329	-34.4655
ZINC14459530	-28.9576
ZINC14459596	-28.1293
ZINC14459606	-21.592
ZINC14459876	-34.894

ZINC14460077	-32.2244
ZINC14460828	-35.266
ZINC14461525	-33.5512
ZINC14468398	-30.7842
ZINC14520627	-36.1505
ZINC14520627	-41.5611
ZINC14531825	-29.9888
ZINC14537294	-26.4409
ZINC14537294	-31.7905
ZINC14538150	-43.8028
ZINC14540550	-33.2132
ZINC14540700	-39.9648
ZINC14542873	-35.3576
ZINC14542874	-34.3724
ZINC14546400	-32.5302
ZINC14546694	-28.5962
ZINC14546795	-26.9585
ZINC14547356	-35.6562
ZINC14548324	-36.3419
ZINC14550961	-36.1874
ZINC14554785	-42.7847
ZINC14607559	-32.7435
ZINC14607559	-37.6098
ZINC14607708	-29.1433
ZINC14607888	-30.9186
ZINC14607888	-34.3416
ZINC14610133	-36.7814
ZINC14610644	-48.1592
ZINC14610915	-45.1739
ZINC14611195	-47.0375
ZINC14625303	-32.1925
ZINC14629137	-46.5874
ZINC14632434	-35.7775
ZINC14632655	-38.4322
ZINC14642852	-44.1617
ZINC14656094	-38.5505
ZINC14660639	-43.1933
ZINC14666079	-27.577
ZINC14668160	-47.3118
ZINC14730764	-34.9186
ZINC14748047	-39.1745
ZINC14748762	-22.6593
ZINC14749681	-32.0882
ZINC14765313	-22.2444

ZINC14770043	-39.4649
ZINC14770700	-38.463
ZINC14783467	-38.5214
ZINC14821962	-46.7726
ZINC14834593	-45.2597
ZINC14880700	-33.4774
ZINC14880718	-27.3726
ZINC14881961	-38.2593
ZINC14881969	-31.1389
ZINC14883023	-30.2236
ZINC14885210	-30.5356
ZINC14886008	-39.6618
ZINC14898235	-44.9272
ZINC14944142	-26.2287
ZINC14944373	-38.3835
ZINC14944414	-37.2563
ZINC14944993	-29.3989
ZINC14960953	-43.6772
ZINC14970426	-28.4007
ZINC14971458	-24.9601
ZINC14975807	-21.2474
ZINC14975807	-25.9135
ZINC14978068	-39.2093
ZINC14981551	-31.5706
ZINC14982248	-24.7928
ZINC14984991	-35.2136
ZINC14985634	-51.3244
ZINC14986066	-35.708
ZINC14992377	-32.9085
ZINC14999961	-36.6682
ZINC15000925	-28.4849
ZINC15002270	-39.9447
ZINC15007999	-49.7129
ZINC15010987	-44.5166
ZINC15015230	-45.0729
ZINC15019228	-27.6557
ZINC15021427	-32.2988
ZINC15035085	-40.1543
ZINC15035610	-44.4929
ZINC15039395	-40.9678
ZINC15048633	-36.8304
ZINC15049874	-41.746
ZINC15050869	-40.3445
ZINC15057208	-54.1215

ZINC15062207	-28.7926
ZINC15062207	-32.9774
ZINC15062488	-36.7284
ZINC15063299	-26.235
ZINC15063299	-34.7841
ZINC15064166	-37.5512
ZINC15066615	-42.4798
ZINC15072263	-41.9169
ZINC15079630	-35.3841
ZINC15081302	-36.0869
ZINC15082127	-28.5873
ZINC15084811	-29.2095
ZINC15085954	-44.7649
ZINC15086017	-30.3052
ZINC15086017	-32.2207
ZINC15086017	-36.8834
ZINC15108220	-39.9524
ZINC15114179	-23.2516
ZINC15125044	-39.2754
ZINC15205352	-40.4571
ZINC15206309	-32.4011
ZINC15206360	-41.1366
ZINC15231398	-29.7369
ZINC15248789	-30.2294
ZINC15250696	-34.0413
ZINC15281214	-38.6432
ZINC15303705	-40.5109
ZINC15306579	-14.4279
ZINC15306579	-27.5204
ZINC15335671	-30.3209
ZINC15354226	-39.5196
ZINC15365057	-31.855
ZINC15401592	-26.2959
ZINC15418331	-35.5864
ZINC15434141	-29.8429
ZINC15435317	-46.9221
ZINC15441714	-29.7352
ZINC15441754	-36.7201
ZINC15453278	-37.8559
ZINC15454115	-37.3581
ZINC15454115	-38.1883
ZINC15455498	-36.6676
ZINC15455592	-30.8181
ZINC15456418	-39.5011

ZINC15464665	-29.67
ZINC15466080	-31.7667
ZINC15497668	-31.3549
ZINC15537580	-43.4487
ZINC15557143	-43.5164
ZINC15562174	-36.713
ZINC15569500	-29.0162
ZINC15570401	-28.0827
ZINC15588483	-40.7294
ZINC15592066	-36.5666
ZINC15658711	-45.9881
ZINC15671781	-40.17
ZINC15675499	-40.5307
ZINC15675894	-40.7298
ZINC15676292	-36.824
ZINC15676292	-42.5261
ZINC15677416	-50.4933
ZINC15679891	-32.4612
ZINC15680028	-35.5277
ZINC15684471	-41.0844
ZINC15707228	-30.016
ZINC15707228	-33.7009
ZINC15707228	-38.039
ZINC15731467	-60.0497
ZINC15768061	-27.746
ZINC15768061	-34.7078
ZINC15768611	-40.7003
ZINC15775392	-40.3341
ZINC15778125	-40.6871
ZINC15778125	-49.2768
ZINC15778149	-36.0775
ZINC15784805	-30.1142
ZINC15786843	-40.4367
ZINC15786844	-42.7998
ZINC15829448	-31.6119
ZINC15829876	-45.423
ZINC15833115	-38.4213
ZINC15833902	-32.5728
ZINC15834433	-28.8307
ZINC15834579	-40.6363
ZINC15842632	-33.2457
ZINC15859899	-40.5588
ZINC15860187	-42.3532
ZINC15860306	-42.5809

ZINC15863969	-27.1441
ZINC15869167	-37.9775
ZINC15904493	-48.0116
ZINC15905454	-43.5718
ZINC15905552	-31.7129
ZINC15910389	-40.0591
ZINC15914347	-35.6184
ZINC15914718	-44.2413
ZINC15932110	-37.8862
ZINC15933214	-38.1286
ZINC15952564	-37.3249
ZINC15952564	-38.9753
ZINC15952564	-41.9911
ZINC15953091	-35.4229
ZINC15956835	-28.0628
ZINC15961651	-24.3408
ZINC15961651	-29.7681
ZINC15961651	-34.1264
ZINC15969136	-29.2274
ZINC15972406	-30.1106
ZINC15986515	-40.3665
ZINC15990364	-24.3721
ZINC15990364	-29.6882
ZINC16001845	-23.9107
ZINC16001845	-26.8417
ZINC16009740	-31.246
ZINC16009877	-31.5123
ZINC16009877	-32.5465
ZINC16019804	-32.5702
ZINC16022207	-29.1147
ZINC16022207	-33.6664
ZINC16024531	-27.7646
ZINC16024923	-25.9002
ZINC16024923	-32.8318
ZINC16032562	-29.303
ZINC16037547	-31.6812
ZINC16085162	-43.5876
ZINC16086102	-25.804
ZINC16088755	-42.6641
ZINC16090693	-33.793
ZINC16121737	-32.8255
ZINC16124529	-31.6378
ZINC16129246	-42.5301
ZINC16158495	-25.0815

ZINC16158770	-31.5051
ZINC16159215	-24.116
ZINC16183738	-43.2363
ZINC16183738	-54.9208
ZINC16184858	-40.9774
ZINC16189349	-35.8504
ZINC16194816	-39.9044
ZINC16213227	-34.2214
ZINC16217209	-37.4436
ZINC16228049	-29.8797
ZINC16228049	-30.1107
ZINC16228049	-31.5514
ZINC16234069	-37.7744
ZINC16243475	-32.822
ZINC16247920	-28.8505
ZINC16265874	-43.3299
ZINC16268219	-37.4484
ZINC16274255	-39.4911
ZINC16281545	-45.0341
ZINC16284633	-38.3411
ZINC16292797	-36.714
ZINC16292837	-25.5289
ZINC16310232	-41.9948
ZINC16310778	-24.2083
ZINC16310778	-29.4556
ZINC16350951	-34.0666
ZINC16352005	-44.0448
ZINC16383082	-38.2346
ZINC16383082	-38.9212
ZINC16401856	-40.8714
ZINC16423597	-34.7187
ZINC16423597	-37.5365
ZINC16429299	-42.5227
ZINC16447799	-36.4659
ZINC16448092	-32.0599
ZINC16449894	-42.4047
ZINC16481328	-47.2273
ZINC16503908	-33.5174
ZINC16575443	-28.6959
ZINC16575443	-33.3783
ZINC16576051	-56.6888
ZINC16578118	-31.5082
ZINC16578118	-40.7442
ZINC16604995	-35.1826

ZINC16622419	-32.8909
ZINC16643489	-36.5341
ZINC16661415	-29.6582
ZINC16662920	-36.1504
ZINC16663057	-33.2364
ZINC16663057	-33.6361
ZINC16663057	-35.1833
ZINC16663445	-34.5774
ZINC16667499	-36.7111
ZINC16672961	-48.1927
ZINC16675842	-35.0105
ZINC16678193	-42.2199
ZINC16682629	-51.2845
ZINC16694385	-33.8882
ZINC16730264	-37.2243
ZINC16733796	-39.5888
ZINC16733796	-42.0944
ZINC16739837	-38.5669
ZINC16741222	-30.201
ZINC16741908	-33.9895
ZINC16742372	-37.9299
ZINC16750516	-29.607
ZINC16752167	-28.787
ZINC16752599	-31.1428
ZINC16790337	-30.5566
ZINC16790338	-25.5491
ZINC16807470	-31.4896
ZINC16808142	-32.7413
ZINC16808376	-33.2949
ZINC16808637	-42.1884
ZINC16808669	-38.2242
ZINC16808916	-36.6663
ZINC16841352	-27.8457
ZINC16841585	-34.8817
ZINC16843704	-41.0985
ZINC16844365	-31.2651
ZINC16844365	-41.1271
ZINC16844650	-44.8913
ZINC16851947	-33.9875
ZINC16855098	-38.8575
ZINC16856166	-38.5445
ZINC16857316	-33.8676
ZINC16880776	-38.5763
ZINC16884616	-31.1658

ZINC16899457	-47.208
ZINC16899559	-35.4986
ZINC16900164	-46.9145
ZINC16901896	-47.818
ZINC16902102	-41.71
ZINC16905813	-45.7447
ZINC16906064	-52.9071
ZINC16911179	-49.8273
ZINC16916187	-39.8632
ZINC16922126	-39.729
ZINC16923064	-39.9381
ZINC16924681	-38.2726
ZINC16924881	-56.2275
ZINC16925000	-45.8217
ZINC16925844	-45.6914
ZINC16930904	-48.6884
ZINC16936319	-47.2554
ZINC16940576	-42.5987
ZINC16948389	-36.091
ZINC16955760	-35.8544
ZINC16958317	-44.7919
ZINC16958817	-31.5498
ZINC16964464	-35.6542
ZINC16970592	-31.2686
ZINC16972905	-32.5919
ZINC16972983	-38.8234
ZINC16989661	-40.2524
ZINC16993926	-36.3555
ZINC17009032	-37.0049
ZINC17015577	-24.5654
ZINC17015577	-33.4189
ZINC17020754	-30.3885
ZINC17021063	-40.6246
ZINC17028119	-34.1597
ZINC17028895	-40.7342
ZINC17060314	-40.1018
ZINC17067226	-43.7827
ZINC17071734	-36.4186
ZINC17071954	-36.0199
ZINC17072682	-31.8422
ZINC17074563	-43.7722
ZINC17076778	-47.619
ZINC17076778	-49.8298
ZINC17077059	-42.01

ZINC17079381	-25.9904
ZINC17081810	-40.0225
ZINC17082666	-45.8788
ZINC17085453	-34.1897
ZINC17085453	-37.1386
ZINC17086327	-31.7062
ZINC17095571	-43.7151
ZINC17095571	-47.5809
ZINC17096532	-38.8621
ZINC17111763	-36.7586
ZINC17112495	-35.9932
ZINC17112655	-27.1098
ZINC17112655	-29.8526
ZINC17112655	-35.3769
ZINC17113602	-34.0161
ZINC17117828	-33.3639
ZINC17138571	-41.577
ZINC17149731	-23.6548
ZINC17156215	-36.7459
ZINC17162657	-32.5982
ZINC17164366	-45.1491
ZINC17169824	-36.5876
ZINC17180061	-33.776
ZINC17180481	-29.2705
ZINC17180481	-39.9884
ZINC17188346	-37.4564
ZINC17197222	-24.8154
ZINC17207663	-45.8958
ZINC17211438	-40.8488
ZINC17211438	-48.333
ZINC17212668	-47.2431
ZINC17242099	-56.4816
ZINC17248991	-46.2235
ZINC17250036	-41.8495
ZINC17250417	-47.613
ZINC17252546	-37.999
ZINC17268850	-36.5508
ZINC17268850	-40.8391
ZINC17309668	-38.0989
ZINC17326077	-38.0477
ZINC17327312	-42.9301
ZINC17353221	-36.2289
ZINC17723776	-36.011
ZINC17730024	-28.4617

ZINC17749224	-37.8497
ZINC17778358	-34.3081
ZINC17780337	-37.6884
ZINC17815812	-48.1282
ZINC17837565	-37.5756
ZINC17857799	-47.3071
ZINC17857799	-52.5097
ZINC17860988	-41.8146
ZINC17862477	-51.8674
ZINC17921318	-27.5275
ZINC17957550	-36.6027
ZINC17959493	-38.229
ZINC17983007	-45.8907
ZINC18039582	-39.7069
ZINC18042398	-33.7368
ZINC18042398	-38.1737
ZINC18067558	-36.8528
ZINC18077457	-43.3013
ZINC18132425	-44.5316
ZINC18140768	-29.5562
ZINC18155179	-33.9356
ZINC18167392	-32.3311
ZINC18167392	-35.9391
ZINC18180976	-37.0124
ZINC18194078	-26.3247
ZINC18194095	-44.444
ZINC18195745	-36.0086
ZINC18195745	-38.078
ZINC18195745	-42.2215
ZINC18196155	-33.5676
ZINC18196155	-35.332
ZINC18196523	-31.3415
ZINC18207504	-23.7532
ZINC18207504	-27.4967
ZINC18209832	-30.2149
ZINC18228763	-29.8382
ZINC18244345	-40.3238
ZINC18250447	-37.7087
ZINC18250447	-46.7227
ZINC18271541	-41.8846
ZINC18271541	-46.3197
ZINC18272329	-44.0497
ZINC18283659	-32.4828
ZINC18283659	-43.4275

ZINC18285519	-27.5747
ZINC18399496	-44.0919
ZINC18532855	-43.3309
ZINC18891191	-34.2407
ZINC18920517	-34.6126
ZINC19022327	-39.0994
ZINC19077952	-40.1654
ZINC19077952	-57.4574
ZINC19090818	-26.3423
ZINC19096629	-27.3826
ZINC19096814	-33.7556
ZINC19096814	-34.0578
ZINC19107607	-40.9289
ZINC19133077	-44.4157
ZINC19133572	-31.3672
ZINC19148619	-38.6282
ZINC19169240	-32.8859
ZINC19169240	-34.8996
ZINC19170488	-29.5699
ZINC19170488	-31.2196
ZINC19170488	-32.0371
ZINC19170488	-32.4487
ZINC19171369	-30.7961
ZINC19216299	-29.7528
ZINC19216539	-25.7036
ZINC19220897	-34.6253
ZINC19220897	-37.663
ZINC19221382	-39.9272
ZINC19235862	-28.3606
ZINC19236699	-30.4408
ZINC19236699	-36.8628
ZINC19288275	-42.6868
ZINC19288275	-46.2755
ZINC19292330	-26.0327
ZINC19292330	-31.2204
ZINC19293231	-37.9876
ZINC19293570	-51.9027
ZINC19293782	-40.2281
ZINC19296183	-28.3163
ZINC19325006	-34.363
ZINC19328565	-32.3957
ZINC19328565	-34.5632
ZINC19328565	-37.1515
ZINC19329197	-33.3766

ZINC19329197	-34.0567
ZINC19336397	-34.7026
ZINC19347807	-39.0273
ZINC19347807	-39.1588
ZINC19355010	-32.5233
ZINC19370011	-32.2633
ZINC19371559	-31.7606
ZINC19371559	-34.5952
ZINC19372431	-39.1118
ZINC19372431	-42.9273
ZINC19376237	-45.6819
ZINC19376237	-50.0111
ZINC19415127	-39.0673
ZINC19438790	-37.4987
ZINC19438790	-41.481
ZINC19440516	-32.6091
ZINC19440516	-32.9984
ZINC19446411	-36.836
ZINC19446411	-43.5079
ZINC19448522	-31.6222
ZINC19456189	-25.2981
ZINC19456189	-27.4907
ZINC19456189	-30.2466
ZINC19476016	-26.1165
ZINC19485788	-33.9895
ZINC19495851	-44.0632
ZINC19496792	-39.6804
ZINC19496808	-34.7089
ZINC19496808	-40.7516
ZINC19497594	-25.0002
ZINC19497594	-32.9703
ZINC19499625	-37.2595
ZINC19517376	-32.215
ZINC19520761	-31.8309
ZINC19526477	-37.6862
ZINC19527990	-37.2329
ZINC19530803	-45.6039
ZINC19530803	-46.524
ZINC19530803	-46.99
ZINC19532689	-44.2042
ZINC19534950	-32.0251
ZINC19535286	-54.4355
ZINC19536644	-29.3553
ZINC19549428	-27.0521

ZINC19549428	-31.5966
ZINC19549787	-18.7574
ZINC19549787	-23.6104
ZINC19549787	-28.3186
ZINC19550143	-33.1978
ZINC19554376	-44.0826
ZINC19560893	-40.0517
ZINC19561057	-39.2612
ZINC19564867	-35.4908
ZINC19568213	-35.3314
ZINC19570035	-49.0648
ZINC19570035	-57.9412
ZINC19571022	-39.0761
ZINC19577926	-32.0469
ZINC19581269	-36.8833
ZINC19581359	-42.184
ZINC19581359	-47.4226
ZINC19582756	-37.5726
ZINC19586511	-34.6492
ZINC19592420	-25.3706
ZINC19592420	-29.6301
ZINC19593740	-35.4283
ZINC19593740	-38.2797
ZINC19593751	-53.2953
ZINC19594358	-49.2602
ZINC19594358	-49.3156
ZINC19594358	-49.3886
ZINC19594655	-33.5324
ZINC19594864	-36.8142
ZINC19595007	-34.6415
ZINC19595007	-45.859
ZINC19658614	-31.3328
ZINC19659476	-37.1287
ZINC19659476	-41.073
ZINC19693943	-32.2093
ZINC19694330	-34.3118
ZINC19695381	-36.0848
ZINC19695381	-38.8138
ZINC19699408	-32.0087
ZINC19700765	-35.7889
ZINC19702066	-25.155
ZINC19703041	-52.9986
ZINC19703041	-55.2294
ZINC19710098	-34.8343

ZINC19716680	-47.2321
ZINC19716816	-29.127
ZINC19716816	-34.414
ZINC19720676	-32.972
ZINC19720676	-53.5386
ZINC19732277	-44.1838
ZINC19735945	-34.0955
ZINC19736442	-43.7714
ZINC19738385	-39.8121
ZINC19746114	-40.4003
ZINC19746114	-48.1482
ZINC19746868	-33.8914
ZINC19747266	-36.8434
ZINC19756243	-48.7736
ZINC19765220	-33.7386
ZINC19765477	-39.3801
ZINC19765933	-38.9388
ZINC19766797	-42.1912
ZINC19766797	-43.2003
ZINC19766820	-38.3876
ZINC19771252	-44.7112
ZINC19774479	-44.762
ZINC19779371	-34.8064
ZINC19790755	-45.5445
ZINC19792805	-44.4867
ZINC19796890	-26.107
ZINC19796890	-28.921
ZINC19797003	-31.7785
ZINC19798186	-41.442
ZINC19798409	-38.3677
ZINC19798409	-45.0408
ZINC19798914	-41.2843
ZINC19798914	-44.2797
ZINC19800575	-27.0235
ZINC19801491	-24.0492
ZINC19802094	-39.0348
ZINC19808025	-40.2852
ZINC19808118	-37.2956
ZINC19809541	-38.4312
ZINC19809541	-48.0768
ZINC19815258	-47.2333
ZINC19815271	-53.1537
ZINC19819757	-41.6545
ZINC19821804	-35.1154

ZINC19825581	-46.315
ZINC19825913	-38.9426
ZINC19831189	-45.2734
ZINC19831189	-45.7644
ZINC19831603	-39.8045
ZINC19834146	-30.5442
ZINC19834146	-31.4125
ZINC19835685	-40.5924
ZINC19838350	-38.1307
ZINC19840086	-33.7669
ZINC19840425	-35.5072
ZINC19844512	-31.6079
ZINC19844512	-35.8977
ZINC19845627	-34.3239
ZINC19851335	-34.7385
ZINC19852411	-21.1218
ZINC19852429	-47.8138
ZINC19853089	-42.0121
ZINC19855484	-41.9015
ZINC19855484	-47.1724
ZINC19863019	-46.6896
ZINC19863923	-39.5326
ZINC19866008	-32.7197
ZINC19872434	-48.8179
ZINC19874664	-51.7376
ZINC19879510	-24.706
ZINC19882609	-27.5235
ZINC19882609	-40.6772
ZINC19883586	-32.6057
ZINC19889426	-32.9932
ZINC19890602	-48.9924
ZINC19891250	-40.8012
ZINC19891459	-36.5142
ZINC19891653	-31.4252
ZINC19891653	-34.9172
ZINC19897843	-33.9592
ZINC19898932	-36.0126
ZINC19899798	-41.5107
ZINC19909211	-48.036
ZINC19910636	-37.6299
ZINC19912400	-32.7756
ZINC19927974	-39.3593
ZINC19938621	-26.8783
ZINC19948272	-37.3085

ZINC19948532	-41.4827
ZINC19964436	-36.7418
ZINC19967897	-34.6379
ZINC19968783	-33.5713
ZINC19968783	-35.3047
ZINC19974057	-39.6721
ZINC19978525	-46.3077
ZINC20027284	-39.732
ZINC20028816	-25.0515
ZINC20045182	-37.0929
ZINC20054150	-43.9434
ZINC20056710	-34.4243
ZINC20058217	-32.5232
ZINC20068979	-34.6512
ZINC20069564	-29.5088
ZINC20070282	-22.0273
ZINC20070305	-31.9778
ZINC20077268	-32.6443
ZINC20077268	-32.7056
ZINC20080107	-33.6305
ZINC20082525	-34.9138
ZINC20082525	-35.608
ZINC20083660	-27.3487
ZINC20083660	-35.0149
ZINC20086390	-37.9193
ZINC20094949	-36.6797
ZINC20112893	-39.2673
ZINC20114235	-29.2178
ZINC20121515	-34.4325
ZINC20122069	-35.9231
ZINC20122069	-35.9936
ZINC20122753	-36.248
ZINC20130193	-38.3895
ZINC20130299	-31.8487
ZINC20130299	-32.0388
ZINC20135457	-45.0054
ZINC20135474	-35.3492
ZINC20136513	-36.1984
ZINC20137624	-42.0092
ZINC20140673	-38.0055
ZINC20141625	-44.1372
ZINC20142367	-42.3957
ZINC20142834	-39.2074
ZINC20144643	-47.8872

ZINC20145174	-45.9365
ZINC20146456	-43.8121
ZINC20149543	-33.7177
ZINC20150803	-26.3281
ZINC20150803	-26.3578
ZINC20152546	-48.8598
ZINC20158318	-47.8499
ZINC20176536	-35.8303
ZINC20176536	-36.9385
ZINC20177672	-27.06
ZINC20192742	-31.2164
ZINC20192742	-36.9348
ZINC20209411	-45.0548
ZINC20217350	-28.373
ZINC20220132	-35.4152
ZINC20220132	-38.5084
ZINC20238120	-44.0961
ZINC20246277	-52.3281
ZINC20247307	-25.1618
ZINC20247448	-35.9732
ZINC20254864	-33.8219
ZINC20254864	-37.182
ZINC20274795	-35.3788
ZINC20275178	-45.0422
ZINC20282680	-31.8289
ZINC20285801	-37.0828
ZINC20290574	-36.2104
ZINC20298567	-39.8157
ZINC20302757	-39.1538
ZINC20308213	-31.512
ZINC20308216	-31.7133
ZINC20308895	-30.7713
ZINC20311052	-39.1782
ZINC20311067	-41.4841
ZINC20315544	-24.0601
ZINC20318515	-38.7745
ZINC20318515	-41.9868
ZINC20319186	-50.9506
ZINC20327777	-30.5591
ZINC20353529	-38.9402
ZINC20357500	-26.8524
ZINC20357637	-33.2043
ZINC20357809	-22.9702
ZINC20357809	-31.8951

ZINC20357900	-36.6377
ZINC20358830	-39.3115
ZINC20360366	-35.5878
ZINC20366272	-28.8905
ZINC20366451	-32.6657
ZINC20367490	-32.7465
ZINC20374483	-30.9164
ZINC20374483	-35.5484
ZINC20374483	-44.6377
ZINC20374736	-40.5736
ZINC20374736	-41.0972
ZINC20375383	-36.1779
ZINC20375580	-32.3553
ZINC20381639	-30.6796
ZINC20389667	-34.6413
ZINC20392089	-29.198
ZINC20392435	-32.8536
ZINC20392494	-27.6974
ZINC20392728	-43.3718
ZINC20393771	-37.135
ZINC20393771	-43.1596
ZINC20404824	-47.1586
ZINC20405413	-36.1309
ZINC20413592	-42.178
ZINC20416480	-35.3157
ZINC20421525	-50.542
ZINC20421586	-37.9417
ZINC20421741	-36.2895
ZINC20426427	-37.3088
ZINC20427081	-41.7394
ZINC20427387	-45.0145
ZINC20436262	-40.0733
ZINC20436387	-46.1201
ZINC20436387	-47.5403
ZINC20444015	-28.0891
ZINC20448479	-41.8659
ZINC20453122	-37.1542
ZINC20454456	-45.8185
ZINC20455778	-32.9969
ZINC20455778	-35.0146
ZINC20463313	-40.266
ZINC20463423	-46.0916
ZINC20467633	-47.1632
ZINC20468909	-28.3007

ZINC20477937	-30.5632
ZINC20486871	-43.6866
ZINC20492256	-34.8375
ZINC20492268	-33.3275
ZINC20496469	-45.7142
ZINC20503372	-36.9824
ZINC20506843	-30.3881
ZINC20508051	-38.3896
ZINC20512198	-38.7377
ZINC20514886	-38.5656
ZINC20528421	-46.2157
ZINC20528724	-45.3909
ZINC20535550	-34.8651
ZINC20535550	-37.5385
ZINC20538801	-35.02
ZINC20538801	-37.486
ZINC20541102	-36.4048
ZINC20541841	-36.8525
ZINC20541841	-40.4752
ZINC20544028	-34.63
ZINC20544028	-35.784
ZINC20547866	-38.911
ZINC20556074	-28.2753
ZINC20556074	-34.3704
ZINC20561747	-37.8622
ZINC20566279	-24.4457
ZINC20566279	-30.6621
ZINC20566419	-36.988
ZINC20567672	-30.158
ZINC20567815	-35.9315
ZINC20572322	-33.6603
ZINC20572498	-30.4995
ZINC20575554	-52.0728
ZINC20587722	-43.9121
ZINC20588752	-33.5922
ZINC20588752	-33.7196
ZINC20588752	-44.3225
ZINC20591998	-34.1665
ZINC20595299	-27.5474
ZINC20595299	-36.753
ZINC20600101	-40.4582
ZINC20601233	-35.6341
ZINC20601233	-43.3978
ZINC20601378	-36.4376

ZINC20602137	-43.4693
ZINC20602738	-48.4476
ZINC20606245	-42.4461
ZINC20608180	-38.9982
ZINC20613078	-36.0668
ZINC20614332	-43.6068
ZINC20614332	-45.1771
ZINC20618018	-36.8918
ZINC20618247	-46.4359
ZINC20618477	-28.6499
ZINC20624371	-34.3225
ZINC20628151	-50.7516
ZINC20628151	-51.5187
ZINC20648371	-57.7909
ZINC20650714	-54.2356
ZINC20675310	-37.2
ZINC20675310	-45.701
ZINC20679803	-29.1985
ZINC20680222	-37.7562
ZINC20686099	-29.1413
ZINC20687282	-34.7954
ZINC20693707	-24.6567
ZINC20694019	-43.7026
ZINC20695696	-37.2294
ZINC20702515	-33.2922
ZINC20707254	-34.5187
ZINC20709050	-27.8266
ZINC20709050	-27.9474
ZINC20709707	-28.3646
ZINC20709707	-35.0745
ZINC20715095	-38.8372
ZINC20715095	-39.2406
ZINC20715095	-40.1831
ZINC20718308	-29.5418
ZINC20718523	-31.2484
ZINC20728305	-28.8012
ZINC20728305	-29.8806
ZINC20738936	-49.3681
ZINC20739241	-47.1558
ZINC20739813	-46.1414
ZINC20748434	-25.9301
ZINC20754518	-28.658
ZINC20755881	-31.6774
ZINC20757990	-37.6166

ZINC20758460	-46.6243
ZINC20758763	-28.2679
ZINC20764672	-26.118
ZINC20799905	-35.4071
ZINC20799905	-39.393
ZINC20808341	-25.8474
ZINC20813457	-29.161
ZINC20816069	-40.2408
ZINC20820844	-36.1952
ZINC20820844	-39.8314
ZINC20828186	-44.4774
ZINC20854988	-31.5524
ZINC20861485	-35.6639
ZINC20869270	-37.1725
ZINC20869270	-41.5938
ZINC20893896	-29.0518
ZINC20893947	-30.3063
ZINC20899247	-2.30923
ZINC20904273	-30.3048
ZINC20906933	-45.9999
ZINC20918731	-41.6315
ZINC20920744	-50.259
ZINC20921571	-42.8164
ZINC20950503	-43.0577
ZINC20973062	-47.2428
ZINC20979483	-45.4386
ZINC20989025	-35.1072
ZINC20990331	-36.5178
ZINC20994168	-45.9896
ZINC21014140	-29.2824
ZINC21014140	-35.8389
ZINC21016928	-43.3396
ZINC21020189	-40.2327
ZINC21020506	-38.3051
ZINC21108012	-38.5765
ZINC21115045	-43.1145
ZINC21127390	-47.3743
ZINC21136429	-40.3742
ZINC21141595	-42.099
ZINC21146048	-45.5113
ZINC21168089	-30.9952
ZINC21168089	-46.4891
ZINC21175135	-40.2448
ZINC21175135	-42.8159

ZINC21175981	-19.2476
ZINC21186817	-34.1125
ZINC21219923	-35.7195
ZINC21228399	-44.1122
ZINC21235196	-53.1877
ZINC21242966	-37.516
ZINC21243314	-46.7391
ZINC21259496	-45.0486
ZINC21331680	-39.8559
ZINC21344464	-38.4271
ZINC21344464	-45.0396
ZINC21359503	-35.6408
ZINC21424596	-34.4988
ZINC21508381	-34.5156
ZINC21577174	-40.6621
ZINC21584400	-36.602
ZINC21584400	-40.3604
ZINC21586887	-39.7469
ZINC21587770	-38.0461
ZINC21620515	-39.4712
ZINC21652816	-41.5556
ZINC21652816	-50.2518
ZINC21653024	-34.2863
ZINC21660479	-25.2701
ZINC21684694	-45.2425
ZINC21695634	-38.3491
ZINC21728080	-38.074
ZINC21732937	-39.484
ZINC21753451	-20.1428
ZINC21774145	-43.3238
ZINC21780212	-49.1555
ZINC21797257	-36.0324
ZINC21799335	-32.0579
ZINC21810903	-33.4236
ZINC21811013	-25.855
ZINC21821395	-39.4792
ZINC21839436	-40.6719
ZINC21846951	-35.1983
ZINC21868339	-35.2058
ZINC21872587	-37.2752
ZINC21873232	-29.3672
ZINC21877675	-35.9247
ZINC21896877	-43.9131
ZINC21915738	-45.6001

ZINC21916322	-44.6119
ZINC21928156	-46.2379
ZINC21929663	-45.164
ZINC21929663	-46.114
ZINC21935426	-55.3108
ZINC21945591	-41.1272
ZINC21956734	-44.5116
ZINC21967269	-39.6334
ZINC21980865	-41.9806
ZINC21985475	-27.0034
ZINC22016968	-39.7338
ZINC22016968	-42.0571
ZINC22018280	-30.5608
ZINC22023231	-43.2509
ZINC22024406	-38.62
ZINC22024406	-41.5067
ZINC22028422	-32.5038
ZINC22028422	-41.9814
ZINC22038956	-45.9595
ZINC22048543	-25.9304
ZINC22048543	-27.771
ZINC22061647	-37.7024
ZINC22061976	-27.3384
ZINC22069708	-34.3537
ZINC22069708	-35.5332
ZINC22096231	-32.4638
ZINC22096231	-38.468
ZINC22126465	-31.9053
ZINC22135465	-39.3227
ZINC22139089	-51.3118
ZINC22200245	-39.8402
ZINC22205933	-40.1753
ZINC22233754	-41.226
ZINC22239200	-38.7542
ZINC22239297	-34.1292
ZINC22240010	-43.668
ZINC22262532	-39.1957
ZINC22262532	-45.5962
ZINC22262532	-45.7115
ZINC22263832	-37.692
ZINC22263832	-37.9498
ZINC22264639	-42.2204
ZINC22264639	-42.7543
ZINC22266651	-28.0857

ZINC22279681	-39.7063
ZINC22370812	-41.6614
ZINC22375385	-34.4894
ZINC22375385	-40.7698
ZINC22418773	-43.2861
ZINC22424713	-37.0406
ZINC22424713	-39.1284
ZINC22440330	-37.7765
ZINC22505072	-38.9514
ZINC22506638	-36.5898
ZINC22516718	-33.9227
ZINC22585585	-40.132
ZINC22589299	-39.8244
ZINC22606923	-41.3963
ZINC22606923	-44.2815
ZINC22606948	-38.4914
ZINC22608562	-30.7601
ZINC22608965	-35.8819
ZINC22609202	-31.2292
ZINC22609202	-39.8769
ZINC22611045	-41.9344
ZINC22618807	-41.8251
ZINC22688959	-25.9758
ZINC22690962	-26.6201
ZINC22697485	-30.4103
ZINC22698285	-34.4629
ZINC22698285	-36.068
ZINC22726587	-39.5525
ZINC22726587	-42.5836
ZINC22734482	-30.2758
ZINC22734482	-32.2431
ZINC22734482	-38.9564
ZINC22734497	-28.1485
ZINC22734497	-29.5136
ZINC22738414	-47.8015
ZINC22753981	-36.2045
ZINC22771422	-34.9915
ZINC22771422	-35.1444
ZINC22773299	-32.6807
ZINC22776889	-39.4995
ZINC22785803	-33.8677
ZINC22788075	-32.489
ZINC22788878	-28.7438
ZINC22791668	-36.97

ZINC22792671	-38.9751
ZINC22793644	-31.1811
ZINC22793644	-38.0948
ZINC22794439	-31.8012
ZINC22794439	-49.3646
ZINC22794439	-52.8626
ZINC22796199	-39.9153
ZINC22796199	-40.979
ZINC22800472	-29.6774
ZINC22800472	-34.4651
ZINC22804449	-34.6436
ZINC22804449	-45.1001
ZINC22831839	-40.6716
ZINC22832717	-38.6935
ZINC22836041	-39.4298
ZINC22851596	-31.8736
ZINC22858889	-28.7698
ZINC22863760	-35.5369
ZINC22866048	-47.1091
ZINC22868399	-30.6805
ZINC22881478	-34.1897
ZINC22887176	-31.5474
ZINC22902503	-30.0458
ZINC22902548	-43.2465
ZINC22909957	-30.7666
ZINC22909957	-33.5
ZINC22912882	-50.4357
ZINC22914973	-33.2128
ZINC22914973	-40.1045
ZINC22917591	-50.1766
ZINC22926488	-34.1491
ZINC22926488	-34.6184
ZINC22943476	-37.8201
ZINC22993288	-33.7718
ZINC22993288	-38.7638
ZINC22995914	-33.3443
ZINC22996805	-41.5834
ZINC23020527	-51.0796
ZINC23022754	-36.1836
ZINC23022754	-37.7672
ZINC23024606	-40.2787
ZINC23041616	-43.0185
ZINC23054300	-35.2962
ZINC23073543	-50.9152

ZINC23080368	-32.3224
ZINC23101310	-39.7556
ZINC23101310	-43.0827
ZINC23114836	-33.5719
ZINC23119133	-37.0442
ZINC23119740	-40.785
ZINC23119740	-41.0967
ZINC23119740	-43.1843
ZINC23129055	-36.9667
ZINC23131201	-39.9434
ZINC23139350	-35.1096
ZINC23140044	-42.39
ZINC23141039	-40.7503
ZINC23141039	-43.9541
ZINC23143647	-43.1894
ZINC23162572	-41.9204
ZINC23188299	-47.1302
ZINC23198250	-40.4091
ZINC23212067	-37.2475
ZINC23212067	-45.845
ZINC23212067	-47.7537
ZINC23231473	-30.5421
ZINC23231473	-37.3792
ZINC23232023	-27.5628
ZINC23239174	-29.3158
ZINC23240651	-49.1628
ZINC23241800	-50.3096
ZINC23241876	-43.0314
ZINC23248885	-40.6536
ZINC23253444	-38.2643
ZINC23262991	-38.5327
ZINC23281562	-32.1625
ZINC23281562	-36.3808
ZINC23286608	-36.9418
ZINC23363280	-39.061
ZINC23363280	-46.5342
ZINC23369034	-44.0817
ZINC23369034	-57.9604
ZINC23375071	-44.0427
ZINC23379776	-35.3566
ZINC23386784	-40.3021
ZINC23389910	-51.2813
ZINC23409854	-39.3531
ZINC23409854	-41.4779

ZINC23430056	-33.1468
ZINC23430809	-35.3025
ZINC23486534	-45.3398
ZINC23492464	-38.3242
ZINC23495794	-36.5651
ZINC23509017	-35.6988
ZINC23509017	-38.4373
ZINC23540871	-30.4253
ZINC23550103	-39.4018
ZINC23625866	-38.7468
ZINC23625866	-47.008
ZINC23643897	-40.3244
ZINC23648919	-37.3307
ZINC23653758	-46.1795
ZINC23653758	-50.9865
ZINC23692316	-45.2458
ZINC23712292	-39.2519
ZINC23717835	-40.4913
ZINC23724608	-41.0855
ZINC23732729	-42.1924
ZINC23751089	-40.5104
ZINC23761213	-40.7926
ZINC23825120	-38.0869
ZINC23846512	-35.6089
ZINC23846974	-30.5486
ZINC23847493	-36.1633
ZINC23847493	-36.8709
ZINC23862386	-35.033
ZINC23894924	-47.7976
ZINC23954682	-37.2161
ZINC23968236	-33.2033
ZINC23975568	-31.9873
ZINC23994273	-35.3568
ZINC23996002	-39.0763
ZINC23996002	-40.9041
ZINC24039729	-38.6772
ZINC24044831	-38.9823
ZINC24058166	-36.788
ZINC24113240	-26.3234
ZINC24113240	-41.8316
ZINC24147530	-32.1762
ZINC24147607	-34.7017
ZINC24193350	-34.6338
ZINC24203780	-29.4903

ZINC24228110	-27.7433
ZINC24236107	-46.416
ZINC24261475	-37.5084
ZINC24315957	-34.6724
ZINC24392131	-41.2309
ZINC24429288	-42.9818
ZINC24444833	-43.367
ZINC24451298	-44.5632
ZINC24451298	-52.8101
ZINC24467475	-36.68
ZINC24480559	-45.9857
ZINC24481795	-42.0494
ZINC24504296	-29.5551
ZINC24510304	-32.0987
ZINC24514037	-38.961
ZINC24527598	-30.9933
ZINC24527598	-36.342
ZINC24529112	-37.3692
ZINC24549208	-31.182
ZINC24622385	-35.0798
ZINC24622385	-40.9947
ZINC24634690	-44.9142
ZINC24666610	-37.3836
ZINC24666610	-43.7471
ZINC24696758	-39.1173
ZINC24717381	-30.32
ZINC24719262	-41.3504
ZINC24792709	-45.0625
ZINC24813522	-48.1635
ZINC24813522	-50.7914
ZINC24828249	-45.6716
ZINC24828432	-44.4151
ZINC24828432	-47.4524
ZINC24843342	-36.4533
ZINC24871577	-28.105
ZINC24871577	-43.9166
ZINC24878645	-38.581
ZINC24878645	-38.7425
ZINC24880051	-41.6306
ZINC24888080	-37.3606
ZINC24897399	-37.4032
ZINC24897399	-43.9121
ZINC24899249	-37.9186
ZINC24899249	-39.4079

ZINC24900103	-31.5666
ZINC24900103	-40.0317
ZINC24906019	-40.2824
ZINC24906019	-40.4325
ZINC24950944	-43.8729
ZINC24962252	-49.5648
ZINC24978661	-39.4278
ZINC24979780	-42.4015
ZINC24986424	-39.8336
ZINC25012121	-40.9132
ZINC25038461	-43.7832
ZINC25039981	-47.8702
ZINC25046544	-40.9632
ZINC25048376	-42.7835
ZINC25064472	-43.7113
ZINC25064472	-46.2776
ZINC25108749	-46.5635
ZINC25113348	-38.9679
ZINC25113348	-42.6427
ZINC25140532	-35.839
ZINC25233946	-41.6443
ZINC25233946	-45.8507
ZINC25248625	-39.9045
ZINC25248625	-45.8431
ZINC25251281	-48.965
ZINC25266583	-38.3805
ZINC25285214	-44.0761
ZINC25309382	-41.0123
ZINC25316703	-41.5359
ZINC25316703	-41.9142
ZINC25321329	-51.8636
ZINC25322049	-42.2557
ZINC25326035	-35.7726
ZINC25330519	-38.3719
ZINC25347853	-43.0297
ZINC25347853	-49.1622
ZINC25381751	-40.8047
ZINC25399370	-35.29
ZINC25399370	-44.1747
ZINC25410483	-46.0314
ZINC25432178	-34.6355
ZINC25438167	-38.678
ZINC25452923	-37.1231
ZINC25460951	-33.2889

ZINC25460951	-35.9453
ZINC25497834	-51.4212
ZINC25498318	-32.9955
ZINC25499512	-37.62
ZINC25502685	-49.9612
ZINC25518518	-36.9261
ZINC25538489	-43.0799
ZINC25549684	-45.617
ZINC25559380	-38.6746
ZINC25574105	-43.8722
ZINC25579875	-40.3934
ZINC25579875	-41.3482
ZINC25694763	-34.9255
ZINC25713189	-41.696
ZINC25741555	-38.3322
ZINC25766818	-43.8288
ZINC25776817	-39.6955
ZINC25778684	-34.9104
ZINC25798256	-44.6207
ZINC25831044	-43.7161
ZINC25831044	-50.1068
ZINC25875822	-40.1203
ZINC25893643	-35.963
ZINC25925704	-36.3483
ZINC25943355	-31.1588
ZINC25943355	-41.0414
ZINC25946651	-34.8568
ZINC25946651	-41.9551
ZINC25968031	-44.9713
ZINC26038406	-49.5352
ZINC26064700	-48.1915
ZINC26083220	-36.2245
ZINC26083220	-41.6864
ZINC26144304	-41.492
ZINC26242021	-38.2548
ZINC26263007	-43.5134
ZINC26263007	-47.8749
ZINC26338475	-45.6678
ZINC26360063	-49.3186
ZINC26361634	-34.6187
ZINC26390117	-46.705
ZINC26423644	-26.9573
ZINC26440861	-43.6713
ZINC26461454	-39.7087

ZINC26476816	-42.4154
ZINC26506737	-23.5266
ZINC26506737	-27.6425
ZINC26513748	-34.0714
ZINC26513748	-37.3462
ZINC26514546	-30.2943
ZINC26537479	-35.0647
ZINC26540064	-38.5218
ZINC26569183	-37.522
ZINC26569183	-41.331
ZINC26569183	-44.9955
ZINC26633694	-29.6677
ZINC26658959	-26.9544
ZINC26658959	-39.4332
ZINC26783901	-40.4681
ZINC26837715	-36.9813
ZINC26875150	-38.6441
ZINC26905080	-36.699
ZINC26918894	-39.5875
ZINC26919984	-41.4863
ZINC26953718	-34.7717
ZINC26993335	-37.3324
ZINC27058286	-33.1335
ZINC27139347	-34.4444
ZINC27154131	-36.9382
ZINC27203424	-37.6852
ZINC27232874	-34.5153
ZINC27317725	-37.1989
ZINC27333738	-38.5958
ZINC27344487	-43.9163
ZINC27366061	-42.634
ZINC27401351	-45.6772
ZINC27416745	-42.289
ZINC27460656	-31.1429
ZINC27496296	-36.8161
ZINC27503657	-40.0113
ZINC27527730	-41.8117
ZINC27527775	-39.9521
ZINC27534022	-44.2339
ZINC27543242	-44.2686
ZINC27543338	-48.5321
ZINC27544355	-36.1146
ZINC27561337	-36.7883
ZINC27570758	-39.5889

ZINC27572151	-48.2079
ZINC27577751	-41.7252
ZINC27577991	-34.5959
ZINC27587506	-32.0558
ZINC27633639	-49.0209
ZINC27666754	-42.8073
ZINC27736193	-39.2342
ZINC27736193	-45.5272
ZINC27764364	-36.5459
ZINC27777701	-38.3617
ZINC27795661	-41.5073
ZINC27824090	-30.7843
ZINC27829423	-37.3701
ZINC27871415	-41.4544
ZINC27908761	-38.1777
ZINC27915328	-37.5304
ZINC27965500	-27.0124
ZINC27965500	-27.4596
ZINC27968816	-40.7312
ZINC27968816	-41.217
ZINC27980689	-36.7748
ZINC27983962	-46.3683
ZINC27986810	-29.3952
ZINC28016208	-43.8203
ZINC28016208	-47.8269
ZINC28021874	-37.7553
ZINC28039403	-39.3558
ZINC28189210	-30.8199
ZINC28194597	-37.6206
ZINC28196918	-37.9141
ZINC28196918	-41.4586
ZINC28221866	-46.911
ZINC28224079	-39.8657
ZINC28230082	-41.0312
ZINC28251097	-36.0248
ZINC28254760	-41.8437
ZINC28273313	-41.9518
ZINC28283966	-38.8484
ZINC28296883	-40.9245
ZINC28307186	-39.5737
ZINC28369751	-31.6149
ZINC28384022	-39.0566
ZINC28417342	-50.717
ZINC28417766	-41.0348

ZINC28417766	-43.9844
ZINC28449820	-50.0323
ZINC28465767	-32.181
ZINC28483353	-35.5063
ZINC28575632	-50.5914
ZINC28620907	-42.3939
ZINC28620907	-50.5864
ZINC28629033	-49.3961
ZINC28634794	-42.3145
ZINC28645237	-45.304
ZINC28785019	-34.133
ZINC28817789	-47.2659
ZINC28835616	-47.0918
ZINC28835616	-55.9668
ZINC28843147	-27.4472
ZINC28924179	-25.434
ZINC28994178	-46.3211
ZINC29242906	-42.1431
ZINC29287901	-38.7646
ZINC29335388	-43.2119
ZINC29342145	-39.4137
ZINC29378325	-38.3628
ZINC29379343	-38.7611
ZINC29386605	-36.0532
ZINC29391386	-40.8902
ZINC29413706	-32.4961
ZINC29433777	-36.9129
ZINC29459734	-36.5186
ZINC29466376	-36.4185
ZINC29490753	-37.3603
ZINC29505803	-40.0173
ZINC29505803	-43.4973
ZINC29525774	-47.514
ZINC29525774	-48.1136
ZINC29576831	-33.4028
ZINC29645640	-22.7714
ZINC29674222	-41.1176
ZINC29723878	-37.2372
ZINC29736721	-46.0736
ZINC29736721	-54.307
ZINC29751042	-38.8356
ZINC29780359	-38.8913
ZINC29785995	-37.2041
ZINC29785995	-38.0337

ZINC29831565	-30.3873
ZINC29831565	-32.8954
ZINC29860374	-44.658
ZINC29866932	-37.7254
ZINC29874707	-48.0912
ZINC29903694	-39.7313
ZINC29934515	-36.5873
ZINC29953636	-35.1447
ZINC29953636	-40.2367
ZINC29963792	-44.4257
ZINC29973022	-34.2649
ZINC29973022	-35.5225
ZINC29979118	-36.9027
ZINC29979118	-44.5073
ZINC30034766	-31.9452
ZINC30034766	-32.3349
ZINC30119879	-41.61
ZINC30119879	-41.6101
ZINC30127664	-36.6081
ZINC30128460	-36.5336
ZINC30132732	-37.2573
ZINC30147556	-36.7198
ZINC30204055	-46.6319
ZINC30287864	-40.1916
ZINC30476173	-50.4708
ZINC30516341	-42.8271
ZINC30516341	-47.6572
ZINC30522562	-33.0559
ZINC30549607	-38.9304
ZINC30571462	-48.1458
ZINC30612617	-41.0853
ZINC30613595	-48.7299
ZINC30692636	-40.5181
ZINC30692636	-46.0868
ZINC30772183	-40.2454
ZINC30874254	-36.648
ZINC30876549	-41.3463
ZINC30900255	-42.8068
ZINC30900255	-43.5865
ZINC30907348	-44.3881
ZINC30989145	-44.2339
ZINC31071442	-44.269
ZINC31095243	-43.6322
ZINC31155557	-27.2252

ZINC31158967	-39.875
ZINC31158967	-43.8029
ZINC31168784	-31.8203
ZINC31264588	-45.2655
ZINC31283408	-43.5459
ZINC31295803	-38.3155
ZINC31297363	-38.9444
ZINC31342581	-43.6174
ZINC31397100	-33.9022
ZINC31398851	-47.0651
ZINC31448797	-34.4366
ZINC31614707	-45.2013
ZINC31649031	-40.4402
ZINC31660792	-41.3477
ZINC31765383	-43.1362
ZINC31775760	-38.9366
ZINC31783766	-44.6712
ZINC31791058	-34.3817
ZINC31799400	-38.6038
ZINC31809061	-33.213
ZINC31809061	-39.2947
ZINC31809061	-43.8396
ZINC31815966	-38.3621
ZINC31829166	-39.4471
ZINC31850663	-40.478
ZINC31850663	-43.3404
ZINC31871099	-44.3471
ZINC31939156	-37.9994
ZINC31945978	-34.0786
ZINC31945978	-36.1791
ZINC31979649	-41.9597
ZINC32029170	-43.3472
ZINC32031117	-42.7712
ZINC32068968	-36.7851
ZINC32073239	-36.0345
ZINC32098818	-27.4961
ZINC32099800	-37.9597
ZINC32100650	-36.6033
ZINC32101592	-41.6286
ZINC32102948	-54.4209
ZINC32102948	-59.0151
ZINC32108238	-35.1116
ZINC32109266	-36.804
ZINC32121291	-27.8597

ZINC32124106	-27.7998
ZINC32127238	-41.2787
ZINC32151018	-37.5133
ZINC32176849	-30.2345
ZINC32181108	-30.9025
ZINC32193624	-30.2618
ZINC32197470	-40.0528
ZINC32202782	-28.3771
ZINC32222876	-26.5025
ZINC32231649	-37.4627
ZINC32250130	-30.9403
ZINC32298816	-42.256
ZINC32298816	-44.7676
ZINC32299594	-30.5456
ZINC32300176	-42.8495
ZINC32302477	-33.9201
ZINC32329567	-40.1819
ZINC32330048	-37.5126
ZINC32331680	-44.5907
ZINC32332027	-42.3822
ZINC32348841	-42.1862
ZINC32375215	-34.1809
ZINC32444372	-28.7421
ZINC32456404	-45.4258
ZINC32477689	-48.0399
ZINC32502208	-40.8133
ZINC32518645	-50.0544
ZINC32518656	-50.1908
ZINC32522371	-29.3333
ZINC32523698	-36.1182
ZINC32523698	-36.9708
ZINC32526043	-40.5684
ZINC32528361	-41.9055
ZINC32534806	-35.2468
ZINC32551677	-38.4606
ZINC32553310	-31.7644
ZINC32555693	-32.6945
ZINC32559910	-24.8213
ZINC32561111	-33.7417
ZINC32579212	-27.4735
ZINC32581815	-38.6664
ZINC32581815	-52.1822
ZINC32587128	-34.9938
ZINC32592823	-38.3895

ZINC32594149	-49.9767
ZINC32595317	-41.374
ZINC32598056	-30.0317
ZINC32598056	-34.5461
ZINC32599836	-33.128
ZINC32599836	-42.604
ZINC32600230	-34.9615
ZINC32601431	-35.269
ZINC32602996	-49.375
ZINC32604874	-34.8744
ZINC32604874	-38.3377
ZINC32604874	-39.4316
ZINC32606074	-42.801
ZINC32608016	-32.2708
ZINC32611006	-46.5554
ZINC32617694	-36.1767
ZINC32617694	-38.6202
ZINC32623895	-45.9902
ZINC32625199	-24.9646
ZINC32625215	-40.0008
ZINC32633729	-38.9209
ZINC32666003	-29.6439
ZINC32666314	-35.5369
ZINC32666314	-38.5792
ZINC32676207	-39.3587
ZINC32676207	-41.662
ZINC32712845	-32.5686
ZINC32712888	-46.4014
ZINC32717162	-45.0341
ZINC32728338	-38.3606
ZINC32734513	-39.3167
ZINC32736256	-41.348
ZINC32738593	-45.8895
ZINC32747156	-40.9224
ZINC32747156	-47.0881
ZINC32748516	-44.7667
ZINC32753713	-37.1621
ZINC32753713	-43.6539
ZINC32754797	-40.0696
ZINC32759373	-44.9848
ZINC32759956	-49.9766
ZINC32762127	-30.0019
ZINC32762127	-40.7017
ZINC32776567	-41.4876

ZINC32799137	-32.4524
ZINC32809749	-32.4157
ZINC32809749	-32.6163
ZINC32815344	-42.3657
ZINC32824991	-38.3418
ZINC32825036	-41.0803
ZINC32825222	-29.5519
ZINC32825222	-35.5843
ZINC32825290	-41.0872
ZINC32825290	-43.3462
ZINC32825604	-42.207
ZINC32830477	-46.5364
ZINC32842874	-43.6908
ZINC32845802	-35.7272
ZINC32865807	-40.6378
ZINC32870619	-33.7214
ZINC32871076	-46.5431
ZINC32879670	-46.8768
ZINC32888153	-43.019
ZINC32893003	-40.4716
ZINC32894396	-33.3699
ZINC32899890	-42.3322
ZINC32901363	-35.0168
ZINC32907513	-37.1361
ZINC32912757	-40.8795
ZINC32912865	-46.8213
ZINC32913086	-41.1396
ZINC32913325	-23.4339
ZINC32913325	-39.6389
ZINC32913343	-36.2182
ZINC32913343	-43.8301
ZINC32915188	-26.8751
ZINC32915372	-41.548
ZINC32918615	-41.7975
ZINC32918776	-32.8469
ZINC32919716	-37.682
ZINC32919967	-29.4317
ZINC32922504	-41.5118
ZINC32923585	-40.2424
ZINC32927010	-44.4204
ZINC32933439	-28.5493
ZINC32933439	-35.4816
ZINC32935316	-23.3714
ZINC32943919	-34.7167

ZINC32945436	-41.5001
ZINC32947929	-37.2392
ZINC32948953	-41.3567
ZINC32960103	-37.7435
ZINC32967105	-35.491
ZINC32969749	-35.0644
ZINC32984469	-42.0314
ZINC32988028	-48.0386
ZINC32988083	-37.781
ZINC33012155	-30.5903
ZINC33026595	-36.6016
ZINC33026595	-43.4656
ZINC33027477	-38.0017
ZINC33034472	-32.0914
ZINC33039897	-47.9462
ZINC33042281	-41.0849
ZINC33048312	-34.9407
ZINC33048312	-46.6754
ZINC33048757	-40.4806
ZINC33049025	-37.2332
ZINC33066005	-40.4396
ZINC33077184	-35.7262
ZINC33077184	-50.8302
ZINC33085263	-43.2582
ZINC33085263	-49.1533
ZINC33086862	-30.4992
ZINC33087248	-27.2694
ZINC33125060	-44.7548
ZINC33126710	-38.037
ZINC33127112	-35.8318
ZINC33128202	-42.8542
ZINC33128853	-39.7296
ZINC33128925	-36.9003
ZINC33137844	-45.0626
ZINC33137918	-31.8603
ZINC33157329	-44.6486
ZINC33157338	-40.9478
ZINC33157395	-34.1368
ZINC33157395	-38.2749
ZINC33169726	-41.5918
ZINC33169726	-42.5414
ZINC33173240	-39.9411
ZINC33206909	-39.6382
ZINC33207834	-44.3056

ZINC33208506	-33.3243
ZINC33208616	-43.3891
ZINC33215924	-50.4475
ZINC33252244	-34.7901
ZINC33255767	-28.9208
ZINC33264019	-28.618
ZINC33264019	-30.6479
ZINC33264019	-36.2259
ZINC33268173	-48.4232
ZINC33270213	-33.0317
ZINC33287348	-27.6767
ZINC33288705	-44.3298
ZINC33297581	-36.4132
ZINC33301989	-53.4308
ZINC33320006	-44.3781
ZINC33320577	-37.7794
ZINC33320577	-43.4878
ZINC33320704	-37.5277
ZINC33321074	-31.586
ZINC33321084	-25.9625
ZINC33324749	-30.1871
ZINC33325585	-20.927
ZINC33327819	-41.0729
ZINC33336620	-36.1634
ZINC33337499	-44.6255
ZINC33337499	-46.5154
ZINC33341096	-47.3246
ZINC33343642	-43.4601
ZINC33369427	-30.1553
ZINC33384448	-39.6702
ZINC33402679	-41.7392
ZINC33406124	-39.0062
ZINC33409349	-45.7038
ZINC33432939	-31.5498
ZINC33438527	-21.5549
ZINC33438660	-30.7139
ZINC33494537	-34.6184
ZINC33522497	-39.0804
ZINC33531358	-22.1569
ZINC33538553	-44.8691
ZINC33541965	-24.4349
ZINC33546997	-41.0546
ZINC33565529	-43.4433
ZINC33568222	-36.4937

ZINC33568222	-47.8771
ZINC33570415	-40.7707
ZINC33624817	-32.7942
ZINC33624817	-46.8788
ZINC33635590	-29.7331
ZINC33648002	-35.667
ZINC33649272	-37.5993
ZINC33662376	-36.9205
ZINC33758273	-32.8983
ZINC33760244	-30.9619
ZINC33769471	-42.3811
ZINC33769471	-46.368
ZINC33770159	-35.7456
ZINC33775027	-20.3798
ZINC33775027	-28.7941
ZINC33810530	-33.2134
ZINC33810978	-41.0976
ZINC33812244	-30.7438
ZINC33820770	-34.3719
ZINC33928810	-26.2697
ZINC33929638	-30.2954
ZINC33929656	-42.1038
ZINC33929865	-53.332
ZINC33934834	-35.8217
ZINC33935388	-36.9079
ZINC33936365	-31.508
ZINC33937941	-44.1756
ZINC33985788	-28.8538
ZINC34050804	-39.627
ZINC34069982	-38.5762
ZINC34106673	-49.0657
ZINC34107540	-46.6398
ZINC34114993	-31.7941
ZINC34116054	-30.6223
ZINC34133121	-35.1876
ZINC34173096	-36.0038
ZINC34190910	-52.4254
ZINC34425447	-28.8996
ZINC34425447	-31.6667
ZINC34638743	-31.1685
ZINC34727089	-36.4955
ZINC34728474	-33.9264
ZINC34781632	-31.7977
ZINC34781667	-35.2821

ZINC34789393	-36.5748
ZINC34791122	-34.921
ZINC34800239	-27.8839
ZINC34800239	-35.7386
ZINC34856464	-22.5629
ZINC34856464	-24.7787
ZINC34860762	-33.4494
ZINC34868084	-44.5388
ZINC34887301	-31.1336
ZINC34911981	-31.4859
ZINC34915965	-35.0135
ZINC34934013	-39.1998
ZINC34938023	-31.5155
ZINC34938023	-33.1443
ZINC34938998	-32.3648
ZINC34939688	-33.885
ZINC34943991	-32.299
ZINC34944445	-24.1642
ZINC34944445	-40.665
ZINC34967380	-34.1841
ZINC34967953	-39.4008
ZINC34972779	-33.2001
ZINC34973255	-33.407
ZINC35004093	-36.1566
ZINC35015530	-38.3767
ZINC35015530	-40.1747
ZINC35018426	-45.0356
ZINC35022535	-30.9302
ZINC35023187	-46.1367
ZINC35043219	-33.5985
ZINC35047063	-40.9352
ZINC35055089	-37.9901
ZINC35100513	-38.1999
ZINC35135230	-41.9685
ZINC35142878	-26.0972
ZINC35146606	-37.6897
ZINC35179493	-37.8802
ZINC35185391	-33.9832
ZINC35205873	-38.1633
ZINC35231149	-34.0174
ZINC35257820	-34.2197
ZINC35269293	-45.3325
ZINC35283228	-45.3655
ZINC35288193	-36.827

ZINC35289730	-34.7186
ZINC35291848	-39.3092
ZINC35311690	-43.621
ZINC35312191	-44.7281
ZINC35312329	-44.8006
ZINC35324216	-38.5827
ZINC35361602	-30.3768
ZINC35378271	-29.327
ZINC35378451	-45.1607
ZINC35392760	-27.6017
ZINC35399277	-27.3761
ZINC35415591	-30.8184
ZINC35415837	-32.1892
ZINC35430472	-44.2267
ZINC35443733	-32.9612
ZINC35443733	-35.9454
ZINC35447787	-23.7816
ZINC35447787	-25.484
ZINC35447897	-31.3458
ZINC35454727	-30.7207
ZINC35460793	-35.6632
ZINC35475147	-31.8555
ZINC35480807	-34.4097
ZINC35505242	-29.1329
ZINC35515789	-24.6079
ZINC35520267	-40.0682
ZINC35520267	-42.1744
ZINC35520544	-30.5215
ZINC35551388	-36.3446
ZINC35561617	-37.7532
ZINC35565847	-30.9623
ZINC35570422	-33.6408
ZINC35576730	-30.6139
ZINC35577438	-40.1018
ZINC35587930	-36.2471
ZINC35598244	-35.3281
ZINC35622239	-31.922
ZINC35622692	-51.894
ZINC35622692	-55.8697
ZINC35622805	-33.0051
ZINC35622805	-33.6167
ZINC35627538	-34.6104
ZINC35682731	-32.0127
ZINC35805207	-35.5327

ZINC35828124	-18.404
ZINC35828124	-27.4147
ZINC35828124	-31.3779
ZINC35828147	-34.418
ZINC35851664	-25.1822
ZINC35851664	-27.8229
ZINC35851790	-27.4305
ZINC35877072	-33.8938
ZINC35877104	-14.1029
ZINC35897654	-37.3487
ZINC35897875	-34.1699
ZINC35923986	-30.9086
ZINC35946758	-42.565
ZINC35946758	-42.7703
ZINC36025454	-34.0287
ZINC36030381	-31.6105
ZINC36047721	-46.4536
ZINC36047721	-47.2426
ZINC36079039	-33.4997
ZINC36095480	-33.1092
ZINC36095597	-38.1509
ZINC36129825	-48.0511
ZINC36139411	-42.1849
ZINC36157522	-41.5394
ZINC36167735	-37.2034
ZINC36167735	-44.2608
ZINC36174035	-42.9749
ZINC36237470	-40.5073
ZINC36243900	-30.507
ZINC36294163	-44.8303
ZINC36325665	-33.6412
ZINC36354921	-36.39
ZINC36357283	-41.0071
ZINC36357283	-41.0695
ZINC36357984	-35.7211
ZINC36360185	-36.3367
ZINC36377919	-34.8494
ZINC36382730	-28.2884
ZINC36386249	-32.5797
ZINC36389817	-39.609
ZINC36392464	-31.3127
ZINC36392464	-39.0807
ZINC36395208	-34.9687
ZINC36395726	-42.3915

ZINC36397800	-38.3788
ZINC36457050	-31.8205
ZINC36457050	-42.8896
ZINC36496276	-52.1789
ZINC36533075	-28.4064
ZINC36533166	-20.4571
ZINC36533593	-35.034
ZINC36533593	-36.7994
ZINC36564325	-33.4746
ZINC36569656	-30.2737
ZINC36580034	-28.3933
ZINC36611150	-39.2908
ZINC36619625	-34.8454
ZINC36625392	-39.5206
ZINC36625457	-29.2035
ZINC36633875	-30.4737
ZINC36633875	-36.9219
ZINC36633875	-38.0492
ZINC36633875	-41.0115
ZINC36646757	-35.2939
ZINC36669090	-34.5344
ZINC36675751	-36.5672
ZINC36683001	-33.9065
ZINC36685862	-51.4385
ZINC36697488	-44.3882
ZINC36704735	-41.2722
ZINC36706888	-32.4799
ZINC36706888	-37.8086
ZINC36709978	-26.9424
ZINC36722408	-30.3351
ZINC36724592	-31.6682
ZINC36724592	-34.9198
ZINC36739120	-35.8842
ZINC36768449	-42.1744
ZINC36793788	-39.9937
ZINC36793851	-36.9551
ZINC36803559	-45.5062
ZINC36812397	-33.2208
ZINC36812397	-33.8254
ZINC36875640	-42.6673
ZINC36877125	-40.6483
ZINC37023424	-31.8379
ZINC37061990	-49.5926
ZINC37133037	-45.5568

ZINC37228236	-31.9694
ZINC37229535	-44.3545
ZINC37246219	-35.4388
ZINC37315191	-35.4227
ZINC37359367	-37.5291
ZINC37359367	-40.227
ZINC37360342	-35.0239
ZINC37396806	-37.9945
ZINC37419137	-28.1734
ZINC37419345	-45.8218
ZINC37448219	-29.2048
ZINC37460363	-33.8917
ZINC37466245	-28.3634
ZINC37489245	-35.6099
ZINC37518268	-49.1934
ZINC37579676	-42.4898
ZINC37624267	-34.3125
ZINC37625483	-28.839
ZINC37632667	-34.2408
ZINC37638266	-35.572
ZINC37638330	-31.7596
ZINC37638463	-24.2172
ZINC37666676	-33.415
ZINC37704458	-32.5917
ZINC37731481	-38.9046
ZINC37731481	-43.4608
ZINC37731489	-40.2018
ZINC37731489	-43.1742
ZINC37740999	-36.6146
ZINC37763792	-32.8197
ZINC37791049	-40.054
ZINC37797055	-36.196
ZINC37866039	-41.267
ZINC37867242	-41.5459
ZINC37867242	-46.8825
ZINC37933947	-34.5351
ZINC37977562	-39.9706
ZINC37979975	-37.2573
ZINC37998545	-39.659
ZINC38078360	-34.8856
ZINC38086674	-33.0643
ZINC38140740	-40.1206
ZINC38141164	-31.1813
ZINC38141164	-35.5898

ZINC38223351	-43.469
ZINC38223351	-47.2502
ZINC38237023	-32.3788
ZINC38255259	-31.9385
ZINC38255259	-36.424
ZINC38491950	-42.2465
ZINC38508067	-40.4267
ZINC38510231	-38.7534
ZINC38519546	-40.58
ZINC38529996	-37.8367
ZINC38575553	-43.8563
ZINC38580305	-30.9815
ZINC38583454	-37.8658
ZINC38587815	-41.5423
ZINC38596728	-44.7954
ZINC38597008	-38.7288
ZINC38597068	-40.1311
ZINC38599295	-36.8259
ZINC38603034	-35.5312
ZINC38605329	-36.312
ZINC38605329	-40.0062
ZINC38610182	-40.404
ZINC38611542	-35.0514
ZINC38611542	-36.5636
ZINC38613880	-34.5373
ZINC38613880	-42.0562
ZINC38615873	-49.3902
ZINC38619459	-32.4516
ZINC38622142	-31.3816
ZINC38630340	-38.0244
ZINC38630340	-44.364
ZINC38630540	-43.6969
ZINC38646193	-37.7658
ZINC38653958	-29.1883
ZINC38659253	-35.2541
ZINC38659393	-37.6158
ZINC38661619	-41.3256
ZINC38671267	-33.8813
ZINC38678404	-39.6247
ZINC38688948	-18.922
ZINC38701769	-11.5611
ZINC38701769	-26.19
ZINC38704459	-43.8775
ZINC38704553	-40.1921

ZINC38704571	-34.3398
ZINC38710425	-36.686
ZINC38710533	-36.0204
ZINC38711730	-38.7017
ZINC38712841	-30.1152
ZINC38722078	-37.4497
ZINC38725682	-39.7458
ZINC38728807	-30.4239
ZINC38728807	-40.3076
ZINC38730946	-45.3695
ZINC38736840	-34.2798
ZINC38736840	-40.809
ZINC38740295	-38.6834
ZINC38741226	-28.9225
ZINC38741226	-42.5677
ZINC38752288	-27.158
ZINC38752839	-40.1826
ZINC38754125	-45.2162
ZINC38754602	-40.0652
ZINC38767532	-31.6456
ZINC38767631	-55.2819
ZINC38767894	-46.1336
ZINC38767901	-46.5746
ZINC38776781	-37.5671
ZINC38776781	-40.7635
ZINC38782376	-37.1623
ZINC38787490	-40.0141
ZINC38787620	-34.6392
ZINC38814575	-45.0643
ZINC38835333	-43.7238
ZINC38840572	-45.329
ZINC38874865	-38.3478
ZINC38890270	-35.6423
ZINC38904970	-43.9693
ZINC38905793	-45.8085
ZINC38906031	-40.8466
ZINC38906193	-38.1578
ZINC38906306	-47.7016
ZINC38907440	-48.6271
ZINC38908430	-30.8912
ZINC38908430	-34.1593
ZINC38917508	-47.4334
ZINC38924825	-42.1124
ZINC38924969	-33.4608

ZINC38932135	-42.9643
ZINC38932135	-50.4447
ZINC38933303	-40.8321
ZINC38943817	-39.0004
ZINC38946807	-49.9445
ZINC38958973	-26.2061
ZINC38959233	-41.7908
ZINC38959362	-31.6067
ZINC38979560	-37.9653
ZINC38979661	-45.6553
ZINC38979757	-28.3275
ZINC38980060	-42.2214
ZINC38980158	-46.8544
ZINC38980158	-50.1037
ZINC38980656	-36.245
ZINC38980954	-46.2273
ZINC38984503	-37.1719
ZINC38984682	-34.0738
ZINC38984682	-38.4622
ZINC38989892	-34.928
ZINC38999571	-27.6645
ZINC39006658	-37.0854
ZINC39006658	-37.7174
ZINC39006658	-39.3531
ZINC39006717	-36.9408
ZINC39007295	-35.0364
ZINC39007295	-45.5954
ZINC39016281	-45.3681
ZINC39016648	-35.7623
ZINC39022335	-32.8309
ZINC39024924	-55.2008
ZINC39029652	-33.3701
ZINC39034889	-40.9624
ZINC39034889	-42.7437
ZINC39055076	-28.4539
ZINC39055270	-26.9064
ZINC39055305	-32.1524
ZINC39055451	-27.0282
ZINC39056944	-28.9861
ZINC39057203	-27.3621
ZINC39085153	-28.7639
ZINC39147799	-24.1129
ZINC39152888	-36.8435
ZINC39164205	-43.8172

ZINC39164422	-22.1688
ZINC39164422	-31.4136
ZINC39214534	-55.0239
ZINC39217266	-35.3953
ZINC39217266	-45.699
ZINC39217598	-21.4625
ZINC39217598	-25.5623
ZINC39218159	-37.2648
ZINC39231769	-33.2632
ZINC39237537	-27.4646
ZINC39237537	-32.1474
ZINC39237697	-46.1874
ZINC39237901	-30.9093
ZINC39239872	-27.968
ZINC39243629	-34.5935
ZINC39257430	-33.792
ZINC39268976	-25.3059
ZINC39276494	-49.7392
ZINC39280075	-30.2465
ZINC39280092	-46.7336
ZINC39288287	-41.755
ZINC39299327	-30.633
ZINC39304040	-42.9427
ZINC39313365	-39.8974
ZINC39314136	-43.0436
ZINC39327746	-36.4212
ZINC39340410	-36.3368
ZINC39345150	-39.5398
ZINC39350910	-35.7998
ZINC39352779	-33.3637
ZINC39352779	-36.6692
ZINC39353023	-37.6219
ZINC39356597	-43.3339
ZINC39358453	-33.6532
ZINC39359959	-29.712
ZINC39360234	-42.1382
ZINC39360369	-40.2871
ZINC39360882	-38.0224
ZINC39360976	-38.5941
ZINC39369803	-44.1215
ZINC39371164	-38.7525
ZINC39373063	-40.4495
ZINC39373302	-39.1054
ZINC39373470	-34.6902

ZINC39380677	-33.5973
ZINC39380716	-44.0209
ZINC39381113	-35.1099
ZINC39383770	-30.6264
ZINC39394123	-21.2001
ZINC39394729	-26.9048
ZINC39394729	-29.4599
ZINC39395766	-27.1104
ZINC39399684	-24.5714
ZINC39404648	-34.6812
ZINC39409778	-31.5524
ZINC39412158	-27.2469
ZINC39421663	-44.3858
ZINC39421930	-44.2627
ZINC39430769	-45.8274
ZINC39437579	-36.2635
ZINC39439911	-17.7757
ZINC39442068	-36.3056
ZINC39454991	-37.4848
ZINC39461405	-34.064
ZINC39461593	-41.1064
ZINC39478536	-35.0271
ZINC39490387	-37.8374
ZINC39490670	-34.1975
ZINC39500368	-34.614
ZINC39518421	-37.9746
ZINC39543138	-31.23
ZINC39551974	-33.0007
ZINC39551974	-40.1067
ZINC39552477	-42.9181
ZINC39572416	-44.4536
ZINC39572423	-43.9558
ZINC39574939	-27.9827
ZINC39586179	-33.7586
ZINC39588241	-38.5551
ZINC39605894	-30.9205
ZINC39617578	-45.5752
ZINC39698322	-28.4388
ZINC39702446	-45.7904
ZINC39707384	-41.4593
ZINC39711733	-41.0229
ZINC39727183	-32.059
ZINC39729729	-36.3405
ZINC39729729	-37.0884

ZINC39736963	-32.3278
ZINC39760575	-30.5286
ZINC39764008	-34.4121
ZINC39776530	-46.0993
ZINC39778619	-35.7553
ZINC39780810	-30.9184
ZINC39843620	-47.4317
ZINC39843978	-26.83
ZINC39844184	-28.3977
ZINC39845101	-32.6406
ZINC39845392	-34.7674
ZINC39845796	-21.4634
ZINC39845985	-25.9901
ZINC39846032	-27.5456
ZINC39848532	-29.5493
ZINC39849454	-28.7324
ZINC39851185	-37.6725
ZINC39851591	-33.8134
ZINC39851844	-40.8366
ZINC39852515	-45.3942
ZINC39854184	-36.3562
ZINC39854712	-35.9917
ZINC39859978	-46.4592
ZINC39860219	-47.6294
ZINC39860474	-40.8744
ZINC39860781	-42.4167
ZINC39861013	-38.1409
ZINC39868100	-45.6969
ZINC39870269	-47.5212
ZINC39870269	-49.0097
ZINC39870610	-38.9448
ZINC39870755	-41.7155
ZINC39871006	-30.187
ZINC39871033	-38.1287
ZINC39871164	-31.0129
ZINC39871391	-42.1425
ZINC39880199	-28.1167
ZINC39880212	-26.3856
ZINC39880224	-36.9492
ZINC39880575	-39.403
ZINC39881113	-32.6345
ZINC39885494	-44.9538
ZINC39885494	-51.3144
ZINC39886407	-33.3197

ZINC39887797	-49.561
ZINC39891733	-36.3056
ZINC39892472	-39.0123
ZINC39893993	-31.6767
ZINC39898777	-22.5122
ZINC39899425	-36.0257
ZINC39899482	-41.8685
ZINC39899495	-37.8929
ZINC39912837	-33.8314
ZINC39916902	-27.0177
ZINC39916902	-29.7915
ZINC39917172	-30.3751
ZINC39917172	-31.3662
ZINC39917181	-31.5043
ZINC39917181	-31.8103
ZINC39918825	-34.679
ZINC39919000	-15.7279
ZINC39930266	-29.5759
ZINC39932362	-50.5321
ZINC39932362	-52.2669
ZINC39935718	-33.1488
ZINC39936148	-42.4246
ZINC39936148	-45.6223
ZINC39939630	-42.3056
ZINC39940188	-40.73
ZINC39940188	-47.4538
ZINC39940598	-38.6595
ZINC39948835	-38.7506
ZINC39949008	-30.3133
ZINC39949234	-40.8813
ZINC39949350	-42.7909
ZINC39950901	-33.6305
ZINC39952830	-39.9621
ZINC39952830	-40.2184
ZINC39952831	-41.4939
ZINC39952831	-41.5124
ZINC39952893	-40.4658
ZINC39952977	-39.3625
ZINC39954197	-47.9151
ZINC39955810	-42.1278
ZINC39956165	-35.726
ZINC39956863	-41.4566
ZINC39961292	-28.2194
ZINC39961400	-42.7411

ZINC39962456	-43.0105
ZINC39967901	-36.4567
ZINC39967901	-41.2771
ZINC39978300	-37.7864
ZINC39978300	-39.5318
ZINC39978991	-35.4198
ZINC39979790	-39.4482
ZINC39995837	-48.3386
ZINC39996160	-35.5635
ZINC40001206	-38.0414
ZINC40004967	-40.2259
ZINC40017439	-44.5584
ZINC40018010	-38.4069
ZINC40024483	-45.7599
ZINC40034258	-50.1997
ZINC40049600	-38.732
ZINC40049956	-39.8403
ZINC40050456	-39.8297
ZINC40050563	-28.937
ZINC40051022	-45.2862
ZINC40052124	-50.033
ZINC40060420	-38.4088
ZINC40063042	-51.4283
ZINC40071398	-36.3675
ZINC40071544	-50.9207
ZINC40072699	-43.8542
ZINC40081266	-28.9037
ZINC40087567	-33.7982
ZINC40087802	-45.2392
ZINC40095312	-36.4415
ZINC40099061	-38.7892
ZINC40106073	-33.0906
ZINC40107133	-32.2404
ZINC40118589	-26.4857
ZINC40119382	-50.3963
ZINC40121816	-40.1326
ZINC40128178	-43.2032
ZINC40128602	-45.8576
ZINC40139240	-39.9107
ZINC40141047	-48.9778
ZINC40142421	-29.3531
ZINC40142421	-35.4665
ZINC40143951	-47.6869
ZINC40144124	-35.5029

ZINC40148178	-28.4639
ZINC40148776	-28.0525
ZINC40153983	-46.8774
ZINC40154266	-43.9333
ZINC40155388	-36.1502
ZINC40156916	-31.0638
ZINC40156916	-43.0159
ZINC40161064	-44.6099
ZINC40161719	-38.4716
ZINC40167703	-43.827
ZINC40173562	-35.1564
ZINC40182213	-30.676
ZINC40216724	-29.3111
ZINC40216724	-31.4908
ZINC40216835	-30.7859
ZINC40216835	-32.0858
ZINC40216890	-37.1257
ZINC40218820	-37.6049
ZINC40229984	-44.8485
ZINC40277149	-40.0489
ZINC40278448	-42.1512
ZINC40284277	-47.8883
ZINC40285907	-37.859
ZINC40287151	-48.1313
ZINC40295514	-31.4046
ZINC40295556	-30.9582
ZINC40296709	-41.379
ZINC40299514	-31.0113
ZINC40299514	-31.4777
ZINC40309831	-43.4531
ZINC40312865	-33.23
ZINC40322184	-41.4403
ZINC40322342	-23.7307
ZINC40322406	-37.1375
ZINC40322585	-34.4572
ZINC40343967	-34.2721
ZINC40351136	-43.5038
ZINC40383898	-37.9462
ZINC40384879	-41.1053
ZINC40406177	-43.6607
ZINC40406177	-43.941
ZINC40422433	-32.992
ZINC40436131	-37.289
ZINC40436131	-39.2634

ZINC40449901	-29.2326
ZINC40450912	-36.5471
ZINC40450916	-37.1218
ZINC40451414	-26.5703
ZINC40455404	-41.1309
ZINC40458720	-33.1559
ZINC40460677	-37.8941
ZINC40460677	-41.2674
ZINC40461955	-33.4768
ZINC40479625	-39.8074
ZINC40493513	-41.88
ZINC40497029	-30.0879
ZINC40497083	-33.4309
ZINC40497083	-39.0454
ZINC40510999	-38.2635
ZINC40514330	-36.1784
ZINC40514475	-38.8672
ZINC40522023	-42.7454
ZINC40522172	-42.5251
ZINC40522822	-46.1635
ZINC40523487	-33.2054
ZINC40536251	-37.061
ZINC40539314	-41.5318
ZINC40542435	-39.7317
ZINC40542957	-39.9356
ZINC40545385	-30.8485
ZINC40546200	-33.2719
ZINC40554267	-38.7544
ZINC40555902	-25.2182
ZINC40563744	-45.4456
ZINC40564571	-39.9271
ZINC40569507	-28.4915
ZINC40571151	-38.2707
ZINC40571151	-40.6201
ZINC40571683	-36.5212
ZINC40572461	-41.1266
ZINC40572631	-44.1047
ZINC40573059	-38.0913
ZINC40581497	-33.2882
ZINC40608852	-37.2963
ZINC40609834	-37.8146
ZINC40611566	-33.7235
ZINC40611566	-36.7638
ZINC40612553	-36.8409

ZINC40612580	-40.4058
ZINC40623856	-43.0812
ZINC40625407	-39.725
ZINC40626448	-29.4403
ZINC40626448	-30.8093
ZINC40628337	-43.0903
ZINC40645492	-41.9549
ZINC40645589	-41.2623
ZINC40645589	-46.7195
ZINC40646279	-39.0501
ZINC40646529	-27.0836
ZINC40646529	-32.1497
ZINC40647590	-34.7655
ZINC40648381	-42.8511
ZINC40657766	-45.3877
ZINC40658607	-40.6789
ZINC40659658	-37.6717
ZINC40659681	-39.6325
ZINC40659706	-35.301
ZINC40659772	-41.6515
ZINC40659776	-42.7708
ZINC40659776	-44.8472
ZINC40660873	-32.0919
ZINC40661580	-49.0854
ZINC40680404	-36.9928
ZINC40687917	-43.3851
ZINC40705478	-30.3831
ZINC40706664	-34.3647
ZINC40708432	-43.0373
ZINC40708781	-41.82
ZINC40708846	-38.5868
ZINC40708846	-43.8839
ZINC40708967	-33.5828
ZINC40708967	-35.0187
ZINC40709001	-39.2291
ZINC40709001	-39.8225
ZINC40709201	-37.0389
ZINC40723512	-37.8552
ZINC40723512	-38.9868
ZINC40727011	-39.6012
ZINC40727194	-52.0011
ZINC40735606	-23.3
ZINC40753525	-46.0205
ZINC40754066	-45.5138

ZINC40755105	-45.8056
ZINC40756077	-44.9725
ZINC40756077	-63.4435
ZINC40756640	-45.3104
ZINC40758036	-44.141
ZINC40758036	-44.5885
ZINC40771142	-29.7366
ZINC40773803	-34.7259
ZINC40774128	-40.1408
ZINC40775156	-29.0119
ZINC40775231	-31.4119
ZINC40775771	-33.9449
ZINC40775771	-37.9359
ZINC40776013	-32.5445
ZINC40776111	-39.6261
ZINC40776489	-30.7945
ZINC40776489	-32.5991
ZINC40778747	-44.4418
ZINC40779254	-36.9704
ZINC40793927	-40.6115
ZINC40797924	-30.5092
ZINC40799498	-34.7198
ZINC40799498	-36.296
ZINC40801208	-35.8598
ZINC40801379	-42.408
ZINC40803209	-38.7073
ZINC40808187	-38.3058
ZINC40808187	-43.6945
ZINC40809326	-42.065
ZINC40809326	-43.8274
ZINC40811353	-41.926
ZINC40811946	-33.9791
ZINC40812002	-37.2974
ZINC40812211	-45.0194
ZINC40812259	-40.0972
ZINC40812758	-39.8162
ZINC40812975	-26.6499
ZINC40813903	-43.2887
ZINC40814590	-29.5049
ZINC40815061	-43.752
ZINC40822123	-40.13
ZINC40822123	-42.2841
ZINC40825168	-47.3083
ZINC40825202	-29.2571

ZINC40825892	-38.4518
ZINC40826444	-39.3435
ZINC40847793	-38.5074
ZINC40888358	-39.6844
ZINC40889320	-36.8151
ZINC40889944	-40.858
ZINC40898908	-40.9368
ZINC40912239	-43.0627
ZINC40940687	-34.3942
ZINC40945441	-32.2214
ZINC40980503	-36.568
ZINC40980503	-36.7175
ZINC41026641	-42.1133
ZINC41026726	-32.8614
ZINC41074364	-33.6145
ZINC41077895	-41.1999
ZINC41082244	-34.808
ZINC41082244	-35.012
ZINC41083093	-29.579
ZINC41108093	-34.3824
ZINC41108232	-28.8915
ZINC41116864	-40.1631
ZINC41146144	-40.1181
ZINC41146414	-35.6402
ZINC41149345	-39.4252
ZINC41149345	-42.1939
ZINC41152450	-30.9014
ZINC41152450	-40.6975
ZINC41153149	-38.1374
ZINC41184852	-36.1544
ZINC41197811	-34.1063
ZINC41213215	-31.2877
ZINC41229119	-31.3872
ZINC41229119	-36.0645
ZINC41275645	-40.5779
ZINC41275645	-44.5393
ZINC41275682	-41.896
ZINC41275682	-46.9905
ZINC41353875	-45.4523
ZINC41359600	-37.3714
ZINC41394401	-38.4291
ZINC41409308	-39.5596
ZINC41431613	-40.6608
ZINC41431972	-37.7244

ZINC41435352	-31.6141
ZINC41438014	-43.8312
ZINC41465523	-33.2145
ZINC41469405	-40.8167
ZINC41489259	-45.352
ZINC41494737	-40.3677
ZINC41496617	-34.7885
ZINC41511169	-39.6968
ZINC41513382	-29.0793
ZINC41513382	-32.7279
ZINC41514904	-34.5641
ZINC41515097	-33.0474
ZINC41515147	-28.7002
ZINC41515147	-42.3847
ZINC41517453	-42.2591
ZINC41537129	-36.5341
ZINC41543726	-27.1672
ZINC41544453	-26.8383
ZINC41544582	-23.5026
ZINC41544973	-50.1888
ZINC41545404	-37.1277
ZINC41545591	-47.3997
ZINC41546987	-43.0017
ZINC41547394	-36.1661
ZINC41553195	-43.716
ZINC41570268	-41.5408
ZINC41574352	-31.9505
ZINC41584886	-24.055
ZINC41584886	-24.3028
ZINC41584887	-28.7337
ZINC41584887	-43.5286
ZINC41586548	-26.3589
ZINC41586548	-30.6915
ZINC41586984	-33.9508
ZINC41586984	-35.9333
ZINC41587558	-41.8619
ZINC41590069	-44.75
ZINC41600634	-33.9872
ZINC41625934	-38.3716
ZINC41625934	-38.8595
ZINC41644892	-48.739
ZINC41645467	-39.3852
ZINC41649196	-26.1569
ZINC41649196	-28.4911

ZINC41649731	-45.4571
ZINC41651523	-23.7244
ZINC41653906	-46.2753
ZINC41655216	-31.8396
ZINC41655216	-41.1655
ZINC41658404	-35.6643
ZINC41658404	-36.8725
ZINC41687747	-46.7696
ZINC41774277	-40.2571
ZINC41818588	-38.4536
ZINC41847877	-38.3627
ZINC41893630	-43.3622
ZINC41909366	-43.7895
ZINC41941408	-35.1755
ZINC41941408	-42.6415
ZINC41952396	-25.3358
ZINC41952396	-40.4557
ZINC42078958	-41.5622
ZINC42092019	-30.467
ZINC42092658	-45.2193
ZINC42099887	-40.6481
ZINC42116249	-38.8489
ZINC42146854	-38.8326
ZINC42160809	-38.842
ZINC42181866	-36.4576
ZINC42203994	-37.2317
ZINC42203994	-40.5777
ZINC42208605	-30.1508
ZINC42208605	-31.988
ZINC42226965	-29.2756
ZINC42226965	-34.2277
ZINC42234311	-33.2629
ZINC42239858	-36.4725
ZINC42242776	-42.1575
ZINC42252291	-45.2509
ZINC42266607	-38.1018
ZINC42280052	-44.2703
ZINC42302765	-49.7215
ZINC42304843	-49.6074
ZINC42306476	-45.5904
ZINC42317918	-36.4035
ZINC42322541	-43.7243
ZINC42334484	-33.3798
ZINC42334484	-39.8953

ZINC42335468	-29.5739
ZINC42335468	-44.2313
ZINC42353569	-38.067
ZINC42353569	-40.9755
ZINC42355797	-37.5994
ZINC42355797	-45.16
ZINC42363609	-37.2853
ZINC42365325	-43.9264
ZINC42366687	-45.2336
ZINC42379258	-50.6442
ZINC42381903	-44.714
ZINC42382232	-48.3156
ZINC42386248	-40.5133
ZINC42390322	-30.1046
ZINC42390322	-30.6422
ZINC42398499	-40.7853
ZINC42398640	-41.8481
ZINC42470476	-44.6464
ZINC42508749	-40.2727
ZINC42534308	-38.4749
ZINC42536149	-35.7717
ZINC42536149	-41.1133
ZINC42566353	-39.1594
ZINC42575518	-37.762
ZINC42625895	-37.0213
ZINC42657521	-37.2787
ZINC42660568	-42.202
ZINC42664875	-46.8819
ZINC42678240	-41.8249
ZINC42696802	-50.7624
ZINC42696812	-42.8657
ZINC42710543	-36.2437
ZINC42710543	-41.6021
ZINC42721677	-37.0142
ZINC42756153	-36.1547
ZINC42838953	-38.1885
ZINC42840169	-36.2732
ZINC42840169	-40.8326
ZINC42840419	-46.1831
ZINC42845687	-38.4701
ZINC42847797	-45.2581
ZINC42898198	-38.5525
ZINC42911496	-37.2517
ZINC42911496	-40.5328

ZINC42923247	-48.0718
ZINC42935367	-38.5627
ZINC42950286	-43.9268
ZINC42950286	-44.985
ZINC42962345	-41.8583
ZINC42975703	-36.5195
ZINC42975703	-40.7185
ZINC42978342	-37.2301
ZINC42978342	-38.4773
ZINC42979179	-32.821
ZINC43044489	-35.7408
ZINC43105702	-34.5389
ZINC43107213	-42.0473
ZINC43133710	-44.0021
ZINC43144167	-41.9677
ZINC43144167	-42.2042
ZINC43160948	-40.8221
ZINC43162517	-39.1195
ZINC43182791	-40.1558
ZINC43272467	-41.5879
ZINC43272486	-43.1355
ZINC43275794	-39.615
ZINC43329041	-44.526
ZINC43454907	-46.1368
ZINC43474952	-36.1598
ZINC43475341	-43.5625
ZINC43527580	-35.8146
ZINC43534032	-45.0509
ZINC43537531	-30.2161
ZINC43574918	-39.1513
ZINC43579355	-36.1863
ZINC43585088	-41.6
ZINC43593098	-40.4393
ZINC43593098	-40.4679
ZINC43607756	-34.5509
ZINC43620241	-37.1074
ZINC43629983	-46.4783
ZINC43630718	-34.8819
ZINC43721217	-40.055
ZINC43730466	-27.9945
ZINC43732513	-32.2468
ZINC43735934	-32.5091
ZINC43736817	-46.3492
ZINC43737181	-32.7128

ZINC43746969	-38.6257
ZINC43746969	-52.8905
ZINC43762344	-37.7417
ZINC43762464	-32.0415
ZINC43762768	-36.0949
ZINC43766844	-35.8179
ZINC43785088	-37.0966
ZINC43828444	-31.5097
ZINC43832822	-42.0592
ZINC43867084	-32.995
ZINC43874169	-42.286
ZINC43875491	-43.1177
ZINC43879480	-45.5598
ZINC43954262	-32.5481
ZINC43963874	-37.191
ZINC43972309	-45.4568
ZINC43972514	-47.6862
ZINC43973021	-41.0831
ZINC43983499	-48.0278
ZINC43990524	-51.8253
ZINC44001701	-48.8748
ZINC44002019	-48.9072
ZINC44002155	-46.9988
ZINC44002196	-37.9331
ZINC44023845	-47.476
ZINC44035093	-36.5684
ZINC44038301	-52.5308
ZINC44041312	-43.5881
ZINC44076173	-30.6839
ZINC44120534	-25.4884
ZINC44136108	-35.0625
ZINC44136143	-25.7821
ZINC44137833	-38.6163
ZINC44137833	-41.8027
ZINC44137967	-35.0848
ZINC44141560	-43.8055
ZINC44158674	-44.3138
ZINC44166833	-27.8935
ZINC44248567	-29.611
ZINC44250302	-24.9246
ZINC44267461	-32.8472
ZINC44302725	-40.5848
ZINC44304853	-47.6402
ZINC44316468	-38.8548

ZINC44317365	-35.6954
ZINC44324559	-44.5934
ZINC44349237	-43.8303
ZINC44390786	-46.0268
ZINC44390838	-27.3673
ZINC44391110	-44.4357
ZINC44396384	-40.606
ZINC44440962	-44.0072
ZINC44443647	-36.1412
ZINC44443647	-46.9456
ZINC44444206	-37.9512
ZINC44444266	-37.5321
ZINC44449239	-38.4824
ZINC44449631	-31.4289
ZINC44452686	-40.2921
ZINC44457310	-38.7994
ZINC44477644	-26.587
ZINC44477644	-33.7833
ZINC44540131	-32.056
ZINC44540131	-32.5084
ZINC44542198	-30.694
ZINC44542198	-39.805
ZINC44548149	-39.3854
ZINC44548284	-34.5976
ZINC44551051	-39.9105
ZINC44551200	-41.8958
ZINC44551200	-45.3558
ZINC44565754	-47.3232
ZINC44567772	-39.0494
ZINC44571925	-47.1969
ZINC44585864	-39.9749
ZINC44637348	-31.0235
ZINC44638922	-41.6598
ZINC44641255	-36.0985
ZINC44642512	-40.0251
ZINC44642655	-35.0967
ZINC44642912	-37.6981
ZINC44697008	-39.9206
ZINC44722231	-47.0824
ZINC44722869	-41.3506
ZINC44738664	-38.8268
ZINC44757974	-38.7209
ZINC44778873	-41.1962
ZINC44789658	-40.6384

ZINC44804308	-40.1709
ZINC44829232	-32.9493
ZINC44831312	-29.6235
ZINC44858666	-31.0256
ZINC44861131	-43.3254
ZINC44867316	-34.9555
ZINC44867688	-36.5491
ZINC44870399	-42.4983
ZINC44889232	-43.3902
ZINC44889651	-39.8615
ZINC44895268	-43.6097
ZINC44913131	-38.2777
ZINC44913590	-34.5174
ZINC44915229	-38.1957
ZINC44947811	-36.6108
ZINC44948532	-39.925
ZINC44950717	-40.4863
ZINC44950717	-46.511
ZINC44954582	-40.6394
ZINC44975300	-35.7485
ZINC44975300	-37.1924
ZINC44978403	-43.2666
ZINC44989715	-46.3941
ZINC44991301	-38.3761
ZINC45013877	-30.1783
ZINC45026150	-37.8769
ZINC45028236	-32.1911
ZINC45071107	-40.0982
ZINC45071232	-26.8979
ZINC45071243	-25.1137
ZINC45076787	-41.7637
ZINC45102546	-39.3475
ZINC45110978	-43.6204
ZINC45126166	-45.7933
ZINC45152584	-47.0464
ZINC45154346	-45.5591
ZINC45154567	-45.526
ZINC45172525	-38.7811
ZINC45191755	-36.1422
ZINC45205790	-34.5594
ZINC45223618	-51.0102
ZINC45248687	-34.1731
ZINC45303710	-35.8875
ZINC45303710	-38.2239

ZINC45307942	-32.6493
ZINC45307942	-34.7875
ZINC45354223	-50.6176
ZINC45415454	-36.7875
ZINC45447706	-24.3808
ZINC45520789	-46.0174
ZINC45522174	-34.8776
ZINC45526877	-28.5888
ZINC45526916	-34.9632
ZINC45526974	-43.6282
ZINC45536681	-43.9615
ZINC45537500	-39.1416
ZINC45537500	-48.4668
ZINC45571577	-46.4406
ZINC45572982	-35.4002
ZINC45572982	-38.6023
ZINC45573582	-34.098
ZINC45578678	-38.6611
ZINC45668706	-39.5773
ZINC45682738	-33.6287
ZINC45682738	-33.6289
ZINC45712481	-48.8145
ZINC45723182	-38.4138
ZINC45723455	-34.2121
ZINC45749779	-32.5637
ZINC45749779	-33.3041
ZINC45753030	-37.2329
ZINC45787000	-26.2946
ZINC45797015	-38.4688
ZINC45822228	-34.9888
ZINC45823055	-41.5077
ZINC45824277	-32.2926
ZINC45824277	-40.4806
ZINC45851855	-33.8349
ZINC45858055	-35.3796
ZINC45869521	-42.7361
ZINC45888750	-39.1675
ZINC45908312	-41.2815
ZINC45921325	-43.7479
ZINC45924654	-43.6425
ZINC45928436	-23.9621
ZINC45942690	-33.0308
ZINC45944551	-40.3141
ZINC45944783	-40.6716

ZINC45947823	-33.6361
ZINC45947823	-36.3289
ZINC45950359	-37.7213
ZINC45954285	-42.9857
ZINC45957038	-35.1106
ZINC45957038	-41.9717
ZINC45958705	-43.8768
ZINC45958983	-32.4285
ZINC45998219	-34.3106
ZINC45999083	-37.0943
ZINC46007435	-32.5649
ZINC46007435	-43.1533
ZINC46012058	-40.9103
ZINC46017444	-41.6235
ZINC46022675	-43.0591
ZINC46030045	-33.9697
ZINC46031291	-49.0303
ZINC46053270	-45.1639
ZINC46053270	-45.5789
ZINC46054573	-39.9861
ZINC46075870	-39.0476
ZINC46079166	-33.5333
ZINC46083525	-46.075
ZINC46083947	-46.1282
ZINC46087714	-41.7899
ZINC46087958	-33.0298
ZINC46087958	-33.9896
ZINC46091144	-35.5688
ZINC46116196	-39.283
ZINC46125111	-40.7533
ZINC46125436	-30.1294
ZINC46125436	-41.0544
ZINC46130358	-41.7689
ZINC46130904	-40.6578
ZINC46132700	-39.4645
ZINC46133343	-33.6063
ZINC46138574	-39.8433
ZINC46146134	-44.403
ZINC46154489	-40.7759
ZINC46159410	-38.0048
ZINC46172049	-36.6385
ZINC46172675	-36.913
ZINC46172890	-42.2572
ZINC46173400	-37.6182

ZINC46173578	-39.401
ZINC46173630	-45.9284
ZINC46269641	-33.9784
ZINC46269641	-36.7466
ZINC46269641	-40.3852
ZINC46269641	-43.6832
ZINC46272302	-41.9362
ZINC46329879	-39.3228
ZINC46353854	-39.5468
ZINC46364613	-46.9468
ZINC46419007	-44.3973
ZINC46444043	-48.3948
ZINC46477403	-32.3219
ZINC46477403	-36.9843
ZINC46484651	-46.572
ZINC46502576	-35.7278
ZINC46502576	-36.0247
ZINC46502754	-39.0793
ZINC46594137	-38.4256
ZINC46648475	-43.5341
ZINC46654557	-31.9979
ZINC46665977	-43.7527
ZINC46708969	-49.3017
ZINC46708974	-48.5818
ZINC46744180	-42.0764
ZINC46813708	-35.3831
ZINC46826409	-41.2319
ZINC46858232	-40.0511
ZINC46869015	-45.7336
ZINC46909027	-32.1131
ZINC46913863	-46.0914
ZINC46914225	-23.1826
ZINC46914225	-32.6366
ZINC46914383	-33.9365
ZINC46927160	-49.4666
ZINC46928082	-46.7637
ZINC46961317	-35.7253
ZINC46970719	-26.1654
ZINC46971163	-37.6547
ZINC46973253	-28.0445
ZINC46995479	-46.1456
ZINC47006484	-44.3928
ZINC47025451	-44.6041
ZINC47031424	-33.9963

ZINC47042075	-50.7731
ZINC47086869	-50.2953
ZINC47131207	-41.9179
ZINC47131714	-38.6576
ZINC47132716	-35.469
ZINC47135111	-43.881
ZINC47135111	-46.6479
ZINC47145411	-26.6416
ZINC47202732	-37.0928
ZINC47202732	-52.1896
ZINC47240280	-35.5131
ZINC47266137	-36.5379
ZINC47295949	-32.7509
ZINC47321080	-33.9412
ZINC47324858	-48.7177
ZINC47350076	-42.3011
ZINC47350951	-48.9471
ZINC47355326	-43.2775
ZINC47374851	-40.6835
ZINC47374851	-48.1956
ZINC47403854	-41.858
ZINC47423866	-45.6644
ZINC47434002	-45.9801
ZINC47476498	-42.8216
ZINC47486634	-31.7806
ZINC47518123	-42.2606
ZINC47542589	-45.5289
ZINC47542732	-45.5958
ZINC47558319	-45.5352
ZINC47559418	-34.4237
ZINC47582254	-37.7612
ZINC47582254	-45.7904
ZINC47596585	-39.9739
ZINC47620437	-40.9837
ZINC47623463	-38.3569
ZINC47626768	-47.4152
ZINC47628445	-39.7686
ZINC47635887	-36.1974
ZINC47640385	-42.4887
ZINC47640890	-37.6615
ZINC47642295	-38.8791
ZINC47642634	-43.1633
ZINC47642975	-31.4385
ZINC47654362	-38.8942

ZINC47655496	-35.2306
ZINC47658801	-48.7482
ZINC47662879	-38.4865
ZINC47665621	-40.1085
ZINC47667691	-42.2556
ZINC47668433	-33.8857
ZINC47668433	-45.7162
ZINC47713794	-41.2385
ZINC47752565	-36.3123
ZINC47752565	-39.8759
ZINC47783327	-48.4327
ZINC47783331	-47.909
ZINC47784731	-42.9124
ZINC47798732	-38.3859
ZINC47807097	-38.7157
ZINC47822426	-33.6328
ZINC47830287	-43.5013
ZINC47832690	-40.7674
ZINC47844988	-35.8781
ZINC47859929	-36.1114
ZINC47859929	-37.652
ZINC47872439	-29.0024
ZINC47872439	-40.2425
ZINC47889135	-39.6977
ZINC47895897	-37.491
ZINC47915947	-33.3571
ZINC47923619	-36.2705
ZINC47940034	-41.527
ZINC47940255	-39.0329
ZINC47940293	-35.9284
ZINC47943212	-35.4129
ZINC47965352	-34.4235
ZINC47971961	-45.5195
ZINC47976627	-47.775
ZINC47979744	-32.5084
ZINC47981174	-35.7033
ZINC47981174	-37.5898
ZINC47981804	-35.755
ZINC48025186	-37.5031
ZINC48025840	-33.8829
ZINC48033066	-54.4658
ZINC48049931	-38.8777
ZINC48050088	-43.2317
ZINC48050302	-42.6122

ZINC48082578	-39.7725
ZINC48082578	-50.4755
ZINC48088024	-37.3906
ZINC48133649	-41.4858
ZINC48133649	-43.7752
ZINC48135263	-42.049
ZINC48166320	-43.1987
ZINC48170779	-33.2506
ZINC48186732	-37.522
ZINC48194480	-41.0801
ZINC48229906	-39.3806
ZINC48235431	-36.4716
ZINC48251548	-37.1654
ZINC48252961	-48.1575
ZINC48254023	-32.3056
ZINC48254061	-35.9907
ZINC48265392	-34.0059
ZINC48268719	-38.2702
ZINC48275726	-45.9583
ZINC48278878	-38.5423
ZINC48288601	-39.7767
ZINC48300893	-34.7778
ZINC48303478	-42.2171
ZINC48304336	-41.0154
ZINC48308567	-40.8844
ZINC48309058	-39.5955
ZINC48310511	-30.3721
ZINC48331546	-37.8969
ZINC48333129	-41.4147
ZINC48345239	-38.656
ZINC48352925	-47.3634
ZINC48363285	-39.3402
ZINC48366937	-45.5296
ZINC48366937	-45.5296
ZINC48369619	-38.7527
ZINC48388759	-37.6341
ZINC48393026	-33.9423
ZINC48393243	-34.8847
ZINC48393243	-35.6327
ZINC48394049	-47.2163
ZINC48408394	-43.609
ZINC48414852	-47.3786
ZINC48414852	-47.896
ZINC48417706	-44.5634

ZINC48434033	-36.4418
ZINC48452213	-46.8562
ZINC48461260	-43.8602
ZINC48461328	-49.9071
ZINC48467894	-41.1878
ZINC48473475	-45.6364
ZINC48474592	-38.5913
ZINC48494635	-43.7835
ZINC48497289	-55.6041
ZINC48501928	-37.8594
ZINC48501982	-36.8485
ZINC48503846	-50.877
ZINC48504015	-51.7038
ZINC48511857	-45.3057
ZINC48519566	-44.9671
ZINC48519758	-42.7123
ZINC48519801	-41.5965
ZINC48520547	-41.0462
ZINC48520547	-41.0971
ZINC48532805	-48.3294
ZINC48568596	-45.1548
ZINC48569017	-41.5666
ZINC48569599	-40.7187
ZINC48569990	-49.7943
ZINC48580435	-42.4091
ZINC48586849	-41.2649
ZINC48587066	-34.6179
ZINC48593514	-37.3458
ZINC48603000	-37.0266
ZINC48603006	-40.6188
ZINC48603252	-39.2142
ZINC48603527	-37.8256
ZINC48609306	-42.8113
ZINC48615176	-38.0071
ZINC48617028	-45.5554
ZINC48629608	-36.7734
ZINC48635080	-47.3092
ZINC48635134	-43.9374
ZINC48642468	-43.6035
ZINC48657991	-34.1015
ZINC48658142	-41.4908
ZINC48658303	-43.5143
ZINC48686171	-41.243
ZINC48694433	-39.0149

ZINC48696064	-34.9101
ZINC48697993	-42.9898
ZINC48703598	-39.1088
ZINC48706076	-41.1025
ZINC48706556	-34.4321
ZINC48706610	-34.5133
ZINC48712057	-37.841
ZINC48715232	-39.6536
ZINC48715232	-45.8617
ZINC48773474	-33.1892
ZINC48806747	-38.2208
ZINC48808853	-39.3893
ZINC48872748	-43.3013
ZINC48875156	-38.7444
ZINC48909968	-46.6986
ZINC48984561	-37.0159
ZINC49006361	-37.7441
ZINC49022881	-47.7265
ZINC49028727	-43.0768
ZINC49044757	-41.0591
ZINC49049867	-41.7246
ZINC49049867	-43.6268
ZINC49059551	-45.0123
ZINC49065647	-37.106
ZINC49065648	-31.2666
ZINC49077867	-39.2182
ZINC49091086	-35.2315
ZINC49092346	-39.4445
ZINC49092346	-40.5497
ZINC49101572	-37.1096
ZINC49102440	-37.1545
ZINC49170697	-28.4736
ZINC49208094	-36.6294
ZINC49314199	-33.127
ZINC49318579	-35.6852
ZINC49318999	-38.2186
ZINC49321655	-42.0755
ZINC49331900	-33.5428
ZINC49365626	-39.664
ZINC49377444	-53.9035
ZINC49394357	-35.3709
ZINC49396237	-42.5481
ZINC49398727	-40.1877
ZINC49402108	-36.7531

ZINC49402138	-26.9657
ZINC49402695	-34.6092
ZINC49402997	-28.6685
ZINC49410377	-38.1062
ZINC49410664	-42.9375
ZINC49410765	-33.204
ZINC49411048	-43.7171
ZINC49412312	-47.7318
ZINC49425892	-40.7775
ZINC49431003	-47.9977
ZINC49431951	-36.5323
ZINC49432542	-51.736
ZINC49432551	-34.3653
ZINC49442769	-32.5879
ZINC49442821	-35.9299
ZINC49442821	-36.4906
ZINC49449736	-46.0975
ZINC49456054	-53.0718
ZINC49456702	-42.8534
ZINC49461402	-43.9968
ZINC49463295	-36.6239
ZINC49466525	-39.0232
ZINC49473186	-38.8271
ZINC49475233	-39.0993
ZINC49477668	-40.3119
ZINC49478277	-38.8093
ZINC49521661	-38.7901
ZINC49521997	-39.1186
ZINC49522004	-40.8495
ZINC49524506	-38.1196
ZINC49543940	-36.4304
ZINC49545177	-41.5499
ZINC49548663	-42.3031
ZINC49549102	-36.6409
ZINC49549102	-40.8165
ZINC49549648	-55.7991
ZINC49550398	-36.7051
ZINC49588566	-26.1339
ZINC49620968	-40.1417
ZINC49669410	-39.2568
ZINC49674761	-43.9449
ZINC49697894	-30.0556
ZINC49697894	-47.6673
ZINC49753220	-36.7566

ZINC49797771	-39.2839
ZINC49797771	-45.0191
ZINC49918886	-30.1564
ZINC49941920	-38.7082
ZINC49943218	-38.4732
ZINC49969716	-34.9895
ZINC49969716	-43.6624
ZINC49973644	-39.9931
ZINC50003678	-46.5969
ZINC50081702	-37.8215
ZINC50091986	-32.808
ZINC50141701	-30.954
ZINC50242441	-34.0592
ZINC50275524	-42.3323
ZINC50275524	-47.8825
ZINC50360796	-46.5273
ZINC50451130	-44.3404
ZINC50683524	-34.0964
ZINC50729711	-41.3515
ZINC50740219	-41.7809
ZINC50901242	-32.3034
ZINC50901242	-35.7887
ZINC50985565	-42.5148
ZINC50985565	-45.8299
ZINC50997889	-32.7945
ZINC51002155	-36.6403
ZINC51087444	-36.6836
ZINC51087444	-42.9415
ZINC51134798	-37.0234
ZINC51182214	-31.3552
ZINC51182728	-38.3044
ZINC51182728	-42.5738
ZINC51210524	-39.3421
ZINC51231017	-38.7003
ZINC51326087	-45.9726
ZINC51347679	-40.1358
ZINC51347679	-47.9993
ZINC51351073	-39.5094
ZINC51417985	-46.8514
ZINC51421499	-33.6034
ZINC51421499	-34.0916
ZINC51421973	-24.3892
ZINC51449491	-51.2182
ZINC51498980	-38.013

ZINC51501008	-35.5921
ZINC51531544	-38.5922
ZINC51531951	-46.9309
ZINC51532103	-41.7587
ZINC51624541	-59.0052
ZINC51739085	-42.484
ZINC51764036	-33.7463
ZINC51764036	-35.0441
ZINC51843688	-50.3274
ZINC51852669	-40.4548
ZINC51872715	-33.4221
ZINC51960458	-44.8235
ZINC52017900	-36.2199
ZINC52041670	-41.3175
ZINC52041670	-47.3837
ZINC52044531	-36.4689
ZINC52044531	-43.1449
ZINC52048982	-37.5892
ZINC52048982	-44.3517
ZINC52084315	-39.2745
ZINC52103131	-32.6457
ZINC52103131	-35.2005
ZINC52105448	-41.116
ZINC52161210	-34.4888
ZINC52161210	-35.7269
ZINC52161699	-30.4416
ZINC52161699	-32.1393
ZINC52163783	-35.0323
ZINC52166336	-46.5481
ZINC52214308	-37.2261
ZINC52214308	-42.467
ZINC52282585	-42.4661
ZINC52282830	-43.8385
ZINC52337609	-52.8573
ZINC52355843	-40.6291
ZINC52362195	-36.9969
ZINC52399132	-45.9584
ZINC52418491	-33.9308
ZINC52418491	-34.2136
ZINC52425829	-38.0133
ZINC52436073	-38.4215
ZINC52458244	-34.6139
ZINC52489017	-47.8214
ZINC52493000	-41.2886

ZINC52535339	-28.9007
ZINC52535339	-34.059
ZINC52582437	-51.4542
ZINC52662483	-38.1487
ZINC52712319	-36.3056
ZINC52712319	-44.9255
ZINC52746994	-37.1197
ZINC52754036	-38.7315
ZINC52779667	-42.5555
ZINC52801508	-30.8418
ZINC52803298	-44.1498
ZINC52816654	-33.8477
ZINC52816654	-36.2382
ZINC52819979	-34.7902
ZINC52928949	-38.9854
ZINC52954562	-43.0182
ZINC52966325	-44.0768
ZINC53016515	-49.4956
ZINC53017147	-34.2899
ZINC53017147	-40.1178
ZINC53065360	-29.3957
ZINC53094923	-35.6548
ZINC53094923	-39.9043
ZINC53184133	-32.2507
ZINC53184133	-37.9862
ZINC53185155	-47.778
ZINC53208480	-41.7932
ZINC53299316	-44.3563
ZINC53330471	-40.1457
ZINC53406243	-46.9064
ZINC53406243	-50.084
ZINC53414552	-28.9675
ZINC53419796	-38.8076
ZINC53419796	-47.0537
ZINC53421749	-37.2913
ZINC53457072	-30.2229
ZINC53506935	-48.6445
ZINC53506935	-49.9246
ZINC53526759	-36.8036
ZINC53537953	-40.5036
ZINC53549825	-45.1537
ZINC53603719	-38.6942
ZINC53603719	-40.9344
ZINC53679774	-41.7325

ZINC53681137	-38.9186
ZINC53681137	-41.2733
ZINC53745968	-45.3704
ZINC53754153	-39.7021
ZINC53759660	-38.7769
ZINC53775862	-49.1077
ZINC53781706	-37.5554
ZINC53790108	-37.0253
ZINC53815025	-35.1524
ZINC53815025	-36.8447
ZINC53835022	-44.2669
ZINC53835725	-30.7143
ZINC53835953	-36.8313
ZINC53837794	-43.0898
ZINC53841005	-37.67
ZINC53906878	-40.001
ZINC53945815	-34.9641
ZINC53994619	-40.8215
ZINC54000820	-42.4846
ZINC54016291	-47.2397
ZINC54017163	-41.4919
ZINC54039614	-41.2904
ZINC54041422	-41.8479
ZINC54042509	-34.0506
ZINC54058503	-39.7149
ZINC54072974	-31.6359
ZINC54092476	-42.7038
ZINC54116808	-41.3126
ZINC54131269	-41.6972
ZINC54137002	-39.7802
ZINC54144946	-36.0817
ZINC54150197	-39.0374
ZINC54151504	-36.0177
ZINC54152482	-41.7031
ZINC54168133	-40.1339
ZINC54175294	-50.5373
ZINC54209331	-40.2918
ZINC54215339	-33.5745
ZINC54215339	-39.2707
ZINC54229150	-43.2395
ZINC54229755	-40.2735
ZINC54229946	-46.974
ZINC54230770	-45.8371
ZINC54234915	-46.5335

ZINC54253442	-32.2113
ZINC54255777	-34.5558
ZINC54313660	-40.1373
ZINC54320232	-49.3107
ZINC54320853	-46.457
ZINC54321639	-50.455
ZINC54322078	-43.9459
ZINC54358602	-40.278
ZINC54358899	-33.0103
ZINC54360344	-30.1067
ZINC54360676	-29.8432
ZINC54361117	-47.7848
ZINC54363517	-35.0624
ZINC54368750	-38.892
ZINC54368750	-46.3442
ZINC54395718	-34.8524
ZINC54410510	-41.9206
ZINC54501145	-42.1967
ZINC54514849	-37.247
ZINC54537335	-22.0963
ZINC54537335	-28.309
ZINC54549196	-33.9047
ZINC54549196	-41.694
ZINC54550416	-35.6408
ZINC54550416	-38.6269
ZINC54551368	-31.1607
ZINC54551368	-34.1863
ZINC54570954	-38.7806
ZINC54570954	-44.7312
ZINC54613061	-37.5466
ZINC54614161	-33.5925
ZINC54619777	-29.2787
ZINC54620536	-39.7273
ZINC54672248	-33.0789
ZINC54807580	-40.7203
ZINC54811891	-35.3432
ZINC54827594	-50.1204
ZINC54913087	-38.7309
ZINC54939483	-44.6216
ZINC54939483	-48.0741
ZINC54977871	-42.517
ZINC54977871	-43.6337
ZINC55006211	-33.5801
ZINC55006211	-35.546

ZINC55009905	-39.9273
ZINC55037313	-34.0512
ZINC55072147	-34.8562
ZINC55076261	-34.723
ZINC55078384	-45.4333
ZINC55090830	-39.3003
ZINC55090965	-43.3951
ZINC55102953	-37.5649
ZINC55107863	-38.8927
ZINC55109144	-23.1076
ZINC55109144	-30.2139
ZINC55111846	-45.5368
ZINC55120493	-38.9986
ZINC55132917	-37.8792
ZINC55146941	-41.0955
ZINC55146941	-48.2839
ZINC55147710	-47.5636
ZINC55148617	-39.0549
ZINC55148617	-43.7419
ZINC55149503	-43.5423
ZINC55154185	-32.35
ZINC55157667	-40.6766
ZINC55166873	-35.0766
ZINC55179245	-40.6317
ZINC55179626	-47.6607
ZINC55179626	-49.4508
ZINC55180250	-32.5196
ZINC55181923	-32.8805
ZINC55183084	-53.8816
ZINC55185466	-41.7052
ZINC55195970	-40.7585
ZINC55195970	-43.1323
ZINC55210083	-39.8575
ZINC55214134	-38.6027
ZINC55215530	-38.6059
ZINC55219280	-40.3806
ZINC55219280	-44.0276
ZINC55253441	-34.674
ZINC55255938	-35.8349
ZINC55255938	-37.6069
ZINC55278978	-38.3004
ZINC55281661	-27.932
ZINC55307118	-49.3985
ZINC55310586	-38.8763

ZINC55327772	-37.6573
ZINC55329074	-39.7391
ZINC55337368	-42.955
ZINC55387277	-31.6376
ZINC55415082	-42.1337
ZINC55415833	-43.0439
ZINC55420326	-48.5693
ZINC55423166	-42.2704
ZINC55494260	-37.3546
ZINC55499059	-43.7789
ZINC55503532	-39.7242
ZINC55519632	-29.4171
ZINC55525746	-30.822
ZINC55525787	-21.9038
ZINC55544732	-30.6177
ZINC55544732	-36.4548
ZINC55558131	-43.0232
ZINC55565498	-41.4858
ZINC55568672	-30.6844
ZINC55574837	-34.45
ZINC55592272	-44.4368
ZINC55691332	-29.0174
ZINC55706833	-40.4437
ZINC55739355	-32.7081
ZINC55769730	-41.4406
ZINC55795994	-34.5484
ZINC55805699	-39.7596
ZINC55818997	-34.0277
ZINC55820109	-44.7693
ZINC55842663	-38.3369
ZINC55846027	-34.4808
ZINC55866930	-39.2657
ZINC55900792	-43.9222
ZINC55942339	-38.0437
ZINC55942592	-39.4546
ZINC55942622	-30.7694
ZINC55947291	-33.1482
ZINC55972109	-39.2678
ZINC55985245	-41.056
ZINC56000948	-40.0878
ZINC56025784	-45.2028
ZINC56034382	-44.1055
ZINC56036735	-40.5984
ZINC56043375	-43.3606

ZINC56044390	-37.3059
ZINC56044390	-43.2671
ZINC56054963	-24.9078
ZINC56054963	-34.5961
ZINC56068050	-39.7108
ZINC56068050	-42.6651
ZINC56068127	-44.0374
ZINC56087080	-43.7101
ZINC56090194	-40.8113
ZINC56144810	-40.3781
ZINC56154333	-45.1106
ZINC56164266	-37.2495
ZINC56164266	-41.7517
ZINC56207305	-37.9787
ZINC56231571	-45.218
ZINC56244115	-44.4926
ZINC56304803	-32.3464
ZINC56342220	-34.6567
ZINC56350814	-42.1581
ZINC56365517	-43.9448
ZINC56369708	-46.4787
ZINC56370726	-37.6885
ZINC56372409	-33.1781
ZINC56397996	-42.2324
ZINC56401890	-36.9813
ZINC56454079	-40.668
ZINC56485572	-42.8392
ZINC56485572	-43.8311
ZINC56511184	-41.8239
ZINC56511295	-44.6016
ZINC56513173	-49.0762
ZINC56533983	-45.6083
ZINC56552750	-25.0526
ZINC56552750	-31.0078
ZINC56575876	-38.6631
ZINC56594379	-50.3743
ZINC56605400	-43.5035
ZINC56662708	-40.7539
ZINC56677880	-39.7218
ZINC56718673	-42.7564
ZINC56721556	-45.6304
ZINC56721556	-46.9386
ZINC56723317	-35.9064
ZINC56739024	-45.7649

ZINC56743515	-40.1094
ZINC56799085	-39.4381
ZINC56815736	-41.7766
ZINC56826645	-30.7093
ZINC56828797	-42.7834
ZINC56863874	-38.6194
ZINC56863874	-43.6602
ZINC56880330	-41.3109
ZINC56901058	-40.6281
ZINC56904884	-42.556
ZINC56905175	-38.9756
ZINC56905552	-46.2829
ZINC56905814	-38.3985
ZINC56906443	-42.72
ZINC56909594	-43.6081
ZINC56911685	-43.7842
ZINC56919753	-44.9154
ZINC56922137	-32.3902
ZINC56925520	-44.0453
ZINC57036433	-47.7703
ZINC57062542	-44.5048
ZINC57062542	-46.1339
ZINC57091791	-39.1152
ZINC57095907	-42.1506
ZINC57101262	-35.6712
ZINC57105440	-26.9491
ZINC57105440	-27.3024
ZINC57136402	-45.4098
ZINC57137537	-34.3655
ZINC57176053	-32.6036
ZINC57182516	-41.0793
ZINC57290225	-48.0448
ZINC57294225	-32.2235
ZINC57312103	-34.4142
ZINC57322937	-44.7114
ZINC57329960	-33.2826
ZINC57331498	-38.2264
ZINC57331498	-39.8352
ZINC57343683	-37.5163
ZINC57360222	-28.9958
ZINC57364705	-50.4385
ZINC57366973	-34.5945
ZINC57369707	-36.857
ZINC57369924	-40.2227

ZINC57369924	-42.4954
ZINC57372411	-38.0768
ZINC57378278	-37.931
ZINC57378294	-45.2694
ZINC57380957	-37.2434
ZINC57423960	-33.544
ZINC57429351	-41.2384
ZINC57464615	-43.2412
ZINC57475043	-43.3464
ZINC57500287	-37.2357
ZINC57511594	-41.4051
ZINC57520275	-35.3302
ZINC57520784	-44.9089
ZINC57569874	-40.4378
ZINC57571023	-41.526
ZINC57589748	-31.8589
ZINC57622510	-37.0462
ZINC57622510	-39.0057
ZINC57622877	-37.1629
ZINC57622877	-37.4016
ZINC57650417	-38.6531
ZINC57659816	-39.449
ZINC57669581	-28.5978
ZINC57669581	-31.1963
ZINC57669589	-25.9697
ZINC57669589	-34.8501
ZINC57670275	-35.9023
ZINC57670275	-37.4926
ZINC57681524	-42.1379
ZINC57681836	-33.654
ZINC57682279	-41.7068
ZINC57710869	-33.4052
ZINC57710869	-40.3569
ZINC57731241	-36.3899
ZINC57731241	-41.011
ZINC57774428	-34.6946
ZINC57781427	-35.9857
ZINC57781427	-48.0553
ZINC57813821	-31.5052
ZINC57813821	-35.0969
ZINC57815639	-39.882
ZINC57836715	-37.5762
ZINC57885204	-35.2129
ZINC57885204	-37.1755

ZINC57893542	-36.3343
ZINC57913931	-43.1068
ZINC57972859	-38.7918
ZINC57972859	-41.0776
ZINC57979033	-36.8887
ZINC57985475	-38.2615
ZINC57998461	-29.804
ZINC57998461	-37.8363
ZINC58010296	-36.7183
ZINC58061250	-36.3734
ZINC58061500	-42.1605
ZINC58061526	-29.8469
ZINC58068686	-43.511
ZINC58075464	-34.9992
ZINC58075464	-39.6389
ZINC58096186	-41.3208
ZINC58100826	-33.5799
ZINC58100826	-34.2937
ZINC58147663	-33.6491
ZINC58152597	-37.2422
ZINC58160453	-42.7915
ZINC58160453	-44.5137
ZINC58160570	-29.2668
ZINC58165421	-42.8093
ZINC58167242	-39.7453
ZINC58175417	-45.8437
ZINC58175476	-33.8341
ZINC58179942	-34.4045
ZINC58182848	-41.6242
ZINC58182848	-44.4407
ZINC58188475	-48.85
ZINC58192964	-36.4106
ZINC58201202	-38.6902
ZINC58206150	-31.5251
ZINC58209171	-44.4179
ZINC58209171	-50.2234
ZINC58217451	-33.5577
ZINC58224060	-45.3268
ZINC58225015	-45.9007
ZINC58225428	-30.8236
ZINC58226614	-42.1472
ZINC58236918	-41.3277
ZINC58244015	-40.223
ZINC58247535	-36.3094

ZINC58264355	-39.6094
ZINC58265310	-37.2153
ZINC58265310	-37.3605
ZINC58268908	-31.1717
ZINC58271981	-33.6596
ZINC58279059	-58.7883
ZINC58281741	-36.2016
ZINC58283077	-46.1862
ZINC58292718	-41.9799
ZINC58297317	-46.4015
ZINC58300020	-41.7371
ZINC58305328	-40.7703
ZINC58313668	-27.9733
ZINC58315858	-32.0472
ZINC58316931	-39.7393
ZINC58316931	-44.2794
ZINC58321222	-37.9456
ZINC58328950	-37.5705
ZINC58328950	-41.0111
ZINC58330407	-31.3052
ZINC58330407	-33.1959
ZINC58341385	-41.6828
ZINC58342630	-44.2168
ZINC58343637	-34.3782
ZINC58343990	-39.5739
ZINC58343990	-44.0097
ZINC58354784	-35.2886
ZINC58358275	-38.5932
ZINC58358275	-43.9679
ZINC58370527	-37.6297
ZINC58392051	-42.5016
ZINC58393512	-39.0054
ZINC58426050	-38.4944
ZINC58465029	-48.9022
ZINC58491340	-41.4953
ZINC58521167	-39.6884
ZINC58540446	-42.8007
ZINC58540446	-43.7274
ZINC58576472	-30.9926
ZINC58593147	-41.5203
ZINC58602146	-38.5955
ZINC58610777	-38.6349
ZINC58619668	-40.1293
ZINC58701921	-43.422

ZINC58754087	-34.3638
ZINC58754087	-36.9697
ZINC58789564	-44.7886
ZINC58800820	-37.5165
ZINC58830357	-38.4445
ZINC58874155	-35.4561
ZINC58886201	-39.3621
ZINC58902092	-36.0852
ZINC58902120	-37.1312
ZINC58930463	-34.7354
ZINC58941147	-46.5645
ZINC58949474	-28.9609
ZINC58949749	-28.9996
ZINC58949755	-27.6133
ZINC58950687	-32.1498
ZINC58953675	-35.4051
ZINC58953675	-41.4795
ZINC58999122	-43.4363
ZINC59041827	-47.6143
ZINC59041903	-39.7094
ZINC59092880	-37.9698
ZINC59106192	-36.6212
ZINC59114067	-38.8907
ZINC59114140	-32.1005
ZINC59128232	-40.1834
ZINC59133947	-37.148
ZINC59134281	-35.7512
ZINC59138592	-46.2747
ZINC59147435	-37.9209
ZINC59147435	-38.457
ZINC59152656	-32.9181
ZINC59152786	-37.2692
ZINC59153297	-39.1436
ZINC59163070	-30.5228
ZINC59165766	-42.0938
ZINC59165895	-38.5802
ZINC59165895	-42.6668
ZINC59166408	-41.732
ZINC59170932	-35.3643
ZINC59231462	-30.4252
ZINC59232271	-40.419
ZINC59232483	-40.3959
ZINC59239093	-37.4468
ZINC59246137	-30.0702

ZINC59249595	-38.9389
ZINC59267184	-34.1695
ZINC59267184	-36.1551
ZINC59271836	-44.7358
ZINC59276711	-31.1111
ZINC59277770	-36.5388
ZINC59293309	-26.7931
ZINC59293346	-21.4794
ZINC59293460	-27.6104
ZINC59293687	-28.3265
ZINC59302085	-44.087
ZINC59302323	-51.5094
ZINC59303457	-48.0547
ZINC59303623	-46.2974
ZINC59304848	-39.6012
ZINC59316049	-43.2803
ZINC59352063	-36.9563
ZINC59366494	-36.8516
ZINC59366754	-40.1922
ZINC59366861	-38.3816
ZINC59366941	-34.197
ZINC59367375	-31.4278
ZINC59367698	-34.1761
ZINC59379690	-45.1619
ZINC59381818	-32.705
ZINC59381904	-42.1149
ZINC59383120	-35.0557
ZINC59384711	-41.0222
ZINC59384711	-45.7598
ZINC59392261	-45.8616
ZINC59397578	-31.2253
ZINC59397578	-42.2588
ZINC59399125	-29.6411
ZINC59400047	-47.3646
ZINC59417712	-44.0401
ZINC59417712	-45.3286
ZINC59418659	-31.1462
ZINC59457269	-42.4052
ZINC59459838	-42.334
ZINC59459918	-37.2145
ZINC59460019	-39.4232
ZINC59460911	-42.4709
ZINC59474727	-39.2674
ZINC59477591	-54.3903

ZINC59477833	-42.3142
ZINC59489150	-38.1847
ZINC59489284	-44.0665
ZINC59491341	-25.4468
ZINC59515246	-51.6224
ZINC59515465	-50.2831
ZINC59519649	-33.5784
ZINC59705280	-41.1365
ZINC59741829	-38.7408
ZINC59761244	-40.9118
ZINC59849469	-50.1061
ZINC59859875	-44.7517
ZINC59862613	-36.3346
ZINC59862613	-43.485
ZINC59936440	-49.4044
ZINC59948016	-34.1709
ZINC59988854	-43.5224
ZINC60197011	-38.5588
ZINC60198489	-36.2294
ZINC60212424	-48.1732
ZINC60282264	-36.506
ZINC60342684	-34.6274
ZINC60342684	-40.7565
ZINC60370253	-37.6255
ZINC60388844	-41.6086
ZINC60445418	-31.3026
ZINC60445418	-47.3252
ZINC60455622	-36.5817
ZINC60461416	-34.4661
ZINC60511540	-32.5049
ZINC60559314	-37.3293
ZINC60582856	-39.5968
ZINC60600410	-41.1904
ZINC60670024	-44.2739
ZINC60738107	-34.6868
ZINC60740776	-32.9893
ZINC60766094	-45.499
ZINC60766432	-36.0844
ZINC60783640	-39.9183
ZINC60785244	-32.6963
ZINC60791711	-42.5193
ZINC60838755	-42.5917
ZINC60865497	-44.035
ZINC60914175	-41.3568

ZINC60914521	-45.2388
ZINC60943836	-34.6871
ZINC60955626	-46.1949
ZINC60965802	-48.9999
ZINC60968756	-39.9756
ZINC60976933	-47.7288
ZINC60978997	-49.1408
ZINC61013071	-33.8859
ZINC61013071	-37.282
ZINC61028355	-37.3491
ZINC61028355	-40.973
ZINC61031065	-40.0524
ZINC61108961	-37.226
ZINC61110656	-42.2135
ZINC61189627	-40.6758
ZINC61210387	-44.931
ZINC61268827	-38.7121
ZINC61354184	-43.108
ZINC61369357	-39.6547
ZINC61399484	-41.5896
ZINC61399484	-44.1296
ZINC61444836	-51.5918
ZINC61465125	-42.5894
ZINC61548089	-37.306
ZINC61553896	-47.1405
ZINC61718107	-49.3151
ZINC61718973	-41.0308
ZINC61718973	-50.3639
ZINC61719052	-38.0488
ZINC61719052	-44.6098
ZINC61720121	-48.3655
ZINC61898108	-48.7005
ZINC61902836	-30.4229
ZINC61905233	-36.2943
ZINC61906146	-38.1399
ZINC61906146	-39.389
ZINC61906683	-32.5892
ZINC61906731	-35.2638
ZINC61906731	-37.1492
ZINC61926087	-31.8198
ZINC61926087	-37.879
ZINC61930112	-22.0136
ZINC61943401	-36.8063
ZINC61958425	-42.9532

ZINC61974478	-43.9576
ZINC61997447	-36.6174
ZINC61997952	-35.9681
ZINC62001376	-41.5822
ZINC62006686	-53.0784
ZINC62023029	-45.7879
ZINC62042279	-45.3854
ZINC62055702	-31.5119
ZINC62129789	-35.9737
ZINC62129789	-39.9608
ZINC62130984	-42.591
ZINC62145905	-42.7965
ZINC62161285	-40.8423
ZINC62250810	-38.7108
ZINC62250810	-38.7941
ZINC62268688	-51.6867
ZINC62319086	-32.6583
ZINC62395601	-51.9452
ZINC62569419	-35.8146
ZINC62674866	-44.0393
ZINC62789949	-38.3324
ZINC63060519	-33.3094
ZINC63068043	-26.2074
ZINC63068043	-37.149
ZINC63093873	-33.8847
ZINC63096928	-40.7782
ZINC63107968	-38.765
ZINC63111260	-44.2905
ZINC63122531	-45.5554
ZINC63137966	-22.5898
ZINC63152286	-34.1243
ZINC63152286	-37.8738
ZINC63160888	-28.0265
ZINC63161135	-40.2365
ZINC63172498	-36.1995
ZINC63180027	-36.4631
ZINC63180027	-37.4884
ZINC63223517	-48.6401
ZINC63225216	-26.1367
ZINC63254309	-43.8559
ZINC63269062	-46.5866
ZINC63280299	-36.3575
ZINC63280299	-37.4963
ZINC63280308	-33.3517

ZINC63280308	-34.3485
ZINC63284792	-26.5689
ZINC63294233	-31.7784
ZINC63330651	-36.656
ZINC63339735	-38.379
ZINC63350555	-28.9491
ZINC63350710	-32.9879
ZINC63351236	-33.674
ZINC63372363	-23.2834
ZINC63372363	-24.6078
ZINC63372375	-23.9524
ZINC63372375	-29.3902
ZINC63375859	-15.6405
ZINC63375859	-33.059
ZINC63376161	-34.3711
ZINC63381322	-35.1546
ZINC63381322	-42.2084
ZINC63393747	-35.698
ZINC63393959	-31.4211
ZINC63393959	-37.7152
ZINC63401882	-43.4751
ZINC63412617	-35.0869
ZINC63415789	-39.8416
ZINC63416385	-34.5991
ZINC63437128	-42.3937
ZINC63437224	-35.6053
ZINC63457982	-30.9198
ZINC63458388	-40.4982
ZINC63472362	-45.0293
ZINC63477181	-47.8264
ZINC63478943	-41.0666
ZINC63486097	-38.8959
ZINC63490387	-42.6906
ZINC63493308	-34.4004
ZINC63493308	-38.662
ZINC63515598	-35.1705
ZINC63515598	-40.9698
ZINC63518520	-30.4263
ZINC63520689	-25.9635
ZINC63520689	-55.051
ZINC63526711	-23.1857
ZINC63526711	-26.9225
ZINC63529380	-36.3659
ZINC63531437	-30.0853

ZINC63531437	-37.9502
ZINC63545969	-44.9476
ZINC63592980	-46.2284
ZINC63593441	-40.1502
ZINC63598002	-38.2115
ZINC63617266	-45.8018
ZINC63617266	-48.6183
ZINC63625624	-43.859
ZINC63625627	-38.2083
ZINC63625839	-38.0649
ZINC63626130	-31.3548
ZINC63626130	-33.5917
ZINC63626193	-29.8539
ZINC63635025	-39.9267
ZINC63637833	-33.62
ZINC63637833	-36.033
ZINC63646934	-42.6226
ZINC63647428	-43.5472
ZINC63660753	-38.7571
ZINC63660753	-47.7029
ZINC63695982	-38.3055
ZINC63695982	-38.5353
ZINC63697783	-32.3282
ZINC63730619	-29.041
ZINC63732948	-48.3764
ZINC63734899	-34.0976
ZINC63752203	-38.2161
ZINC63766461	-38.638
ZINC63786339	-36.8647
ZINC63795536	-33.7723
ZINC63803313	-47.0543
ZINC63807975	-39.7701
ZINC63808173	-36.9865
ZINC63818596	-38.4438
ZINC63820690	-53.3295
ZINC63831457	-37.7158
ZINC63845710	-47.967
ZINC63863551	-38.6855
ZINC63863596	-38.7029
ZINC63870572	-28.8865
ZINC63870851	-40.2727
ZINC63871970	-44.4517
ZINC63872003	-36.2482
ZINC63872023	-43.8563

ZINC63872023	-46.0444
ZINC63881662	-39.0139
ZINC63883586	-48.1016
ZINC63884517	-35.5283
ZINC63884517	-37.5196
ZINC63885262	-31.4849
ZINC63885262	-43.0028
ZINC63899734	-29.7654
ZINC63925876	-33.5925
ZINC63925876	-41.4667
ZINC63926092	-42.3518
ZINC63926341	-45.803
ZINC63927077	-39.3239
ZINC63949821	-44.0817
ZINC63952283	-46.6904
ZINC63952627	-34.1433
ZINC63959116	-40.0301
ZINC63963000	-45.4178
ZINC63985184	-27.7374
ZINC63985747	-22.8085
ZINC63985747	-32.8882
ZINC63985960	-43.9351
ZINC63985960	-47.9345
ZINC63996624	-34.0347
ZINC64002465	-35.6974
ZINC64006878	-29.0486
ZINC64024292	-25.1255
ZINC64032169	-44.5483
ZINC64040328	-17.6709
ZINC64045327	-36.752
ZINC64058432	-45.0596
ZINC64066950	-31.9226
ZINC64067025	-31.3345
ZINC64067025	-32.6143
ZINC64093825	-31.9991
ZINC64111138	-22.8544
ZINC64164284	-29.079
ZINC64164284	-42.9634
ZINC64166188	-28.9718
ZINC64166188	-34.0109
ZINC64166383	-45.5313
ZINC64279956	-39.749
ZINC64320207	-31.3869
ZINC64320355	-33.9026

ZINC64334328	-32.5659
ZINC64334328	-37.1429
ZINC64334472	-39.7601
ZINC64334472	-40.7283
ZINC64337250	-31.492
ZINC64337250	-33.9486
ZINC64349031	-42.2053
ZINC64442953	-34.6113
ZINC64467698	-29.3458
ZINC64467698	-38.082
ZINC64471506	-49.7419
ZINC64482419	-45.782
ZINC64492052	-38.9398
ZINC64517535	-28.9403
ZINC64537311	-47.0458
ZINC64552219	-35.0998
ZINC64553402	-36.124
ZINC64567878	-36.9681
ZINC64568209	-32.4484
ZINC64572938	-45.2477
ZINC64592151	-36.627
ZINC64606845	-38.6775
ZINC64611430	-27.7073
ZINC64662324	-43.2068
ZINC64668149	-29.8377
ZINC64674506	-36.4894
ZINC64676577	-38.2168
ZINC64702631	-47.0715
ZINC64702703	-45.5812
ZINC64714262	-52.5477
ZINC64719100	-38.3662
ZINC64740505	-50.588
ZINC64741096	-30.0063
ZINC64741359	-31.8836
ZINC64743894	-34.8221
ZINC64749949	-44.682
ZINC64756466	-40.8058
ZINC64787596	-29.7135
ZINC64788653	-50.1284
ZINC64788653	-53.4509
ZINC64788731	-27.341
ZINC64788731	-40.1702
ZINC64789081	-26.8414
ZINC64789081	-39.2616

ZINC64789158	-35.9194
ZINC64808332	-36.4881
ZINC64812727	-49.3486
ZINC64825445	-48.0467
ZINC64833329	-36.1209
ZINC64835811	-45.9753
ZINC64843832	-40.6739
ZINC64843832	-41.7963
ZINC64851899	-46.7958
ZINC64862786	-37.0256
ZINC64865295	-43.1547
ZINC64869219	-29.6084
ZINC64876944	-35.3275
ZINC64895728	-35.0696
ZINC64896181	-44.3368
ZINC64899067	-26.6402
ZINC64916705	-43.1859
ZINC64927043	-33.0994
ZINC64929451	-36.3847
ZINC64929451	-41.9035
ZINC64979205	-33.4457
ZINC64979213	-33.8532
ZINC65003883	-41.6596
ZINC65009348	-42.5383
ZINC65058248	-30.9634
ZINC65062623	-37.5131
ZINC65090551	-41.645
ZINC65090551	-45.0381
ZINC65097308	-39.8626
ZINC65120173	-38.9489
ZINC65120173	-40.3382
ZINC65120458	-49.4359
ZINC65141472	-45.0213
ZINC65147822	-47.4482
ZINC65160300	-43.618
ZINC65177863	-38.2001
ZINC65177863	-41.6185
ZINC65183356	-32.8871
ZINC65210050	-37.6633
ZINC65235324	-38.1129
ZINC65238736	-41.916
ZINC65241808	-28.5227
ZINC65241847	-34.5456
ZINC65246647	-34.517

ZINC65288787	-39.0661
ZINC65335482	-37.8137
ZINC65355614	-34.9648
ZINC65355614	-38.3486
ZINC65356826	-26.9194
ZINC65356826	-27.4993
ZINC65357253	-34.9923
ZINC65369808	-39.6328
ZINC65370073	-40.0412
ZINC65370499	-34.5068
ZINC65371888	-40.3322
ZINC65373305	-45.6698
ZINC65383567	-27.2047
ZINC65383567	-37.138
ZINC65386367	-34.3914
ZINC65387522	-31.9754
ZINC65387522	-33.1597
ZINC65404151	-40.1157
ZINC65404151	-43.6844
ZINC65404397	-36.9033
ZINC65404397	-39.4878
ZINC65408273	-25.9013
ZINC65415568	-42.117
ZINC65420565	-36.9374
ZINC65420565	-41.7606
ZINC65421487	-37.6973
ZINC65421487	-42.4369
ZINC65428157	-35.191
ZINC65428157	-35.3395
ZINC65428310	-43.0829
ZINC65430334	-37.7028
ZINC65431062	-43.7359
ZINC65431434	-24.4002
ZINC65433561	-32.2026
ZINC65433561	-39.7217
ZINC65433930	-40.3462
ZINC65435727	-41.7281
ZINC65435727	-50.5853
ZINC65444132	-39.4842
ZINC65444132	-42.5449
ZINC65449447	-38.1216
ZINC65453020	-41.6791
ZINC65454231	-32.8888
ZINC65454231	-38.6079

ZINC65456572	-30.0722
ZINC65456572	-34.1132
ZINC65458481	-31.9109
ZINC65460491	-38.0379
ZINC65460491	-39.8205
ZINC65466538	-26.1301
ZINC65470585	-39.1309
ZINC65476867	-33.1687
ZINC65476867	-35.5937
ZINC65476924	-35.0999
ZINC65487748	-35.8641
ZINC65497490	-40.401
ZINC65501801	-35.6219
ZINC65507660	-36.1438
ZINC65514733	-40.0351
ZINC65514733	-45.7029
ZINC65527611	-31.7201
ZINC65527611	-35.0228
ZINC65531959	-37.9562
ZINC65535426	-46.0357
ZINC65543073	-45.069
ZINC65548233	-44.0359
ZINC65548302	-32.5337
ZINC65552646	-37.2683
ZINC65565411	-40.8263
ZINC65567340	-41.1439
ZINC65592490	-34.7669
ZINC65593984	-51.3597
ZINC65596130	-40.2519
ZINC65608187	-37.3074
ZINC65616642	-47.0306
ZINC65643345	-45.8348
ZINC65724825	-40.2796
ZINC65726083	-41.9201
ZINC65738312	-43.0876
ZINC65742824	-40.5
ZINC65744355	-50.8494
ZINC65764398	-52.4404
ZINC65770211	-35.6617
ZINC65770211	-41.6927
ZINC65781978	-50.7954
ZINC65950699	-42.7865
ZINC65965193	-28.9415
ZINC65965193	-37.6323

ZINC66004217	-44.7377
ZINC66006541	-27.1283
ZINC66036716	-36.527
ZINC66077589	-31.0053
ZINC66077589	-33.396
ZINC66089798	-32.4267
ZINC66107140	-43.4901
ZINC66113948	-43.7462
ZINC66114580	-42.2255
ZINC66127155	-42.6543
ZINC66134104	-43.624
ZINC66134815	-32.9982
ZINC66141147	-35.1711
ZINC66143042	-33.9904
ZINC66143042	-36.179
ZINC66143455	-45.5744
ZINC66155932	-36.203
ZINC66155932	-38.017
ZINC66172156	-32.9177
ZINC66173533	-50.4085
ZINC66182694	-40.4377
ZINC66183048	-35.4153
ZINC66190791	-33.1154
ZINC66200943	-37.0154
ZINC66211898	-44.3986
ZINC66236739	-36.1754
ZINC66236739	-38.1282
ZINC66256263	-43.2586
ZINC66263686	-33.8111
ZINC66264204	-43.7775
ZINC66277557	-38.4938
ZINC66293259	-39.3313
ZINC66301525	-31.1321
ZINC66314730	-39.8088
ZINC66318847	-43.6925
ZINC66319050	-20.5685
ZINC66324255	-42.0148
ZINC66336239	-41.8049
ZINC66338862	-34.0734
ZINC66342935	-39.5491
ZINC66354314	-39.0826
ZINC66370779	-43.0939
ZINC66370779	-43.4996
ZINC66377507	-42.5244

ZINC66380315	-38.8053
ZINC66391793	-35.4072
ZINC66393113	-36.1634
ZINC66394298	-37.3442
ZINC66394428	-40.9885
ZINC66398955	-36.6119
ZINC66400910	-36.5152
ZINC66401730	-44.7964
ZINC66403111	-33.7668
ZINC66403130	-40.5636
ZINC66449890	-38.2126
ZINC66449890	-43.2374
ZINC66452093	-39.0105
ZINC66458953	-33.5005
ZINC66470945	-52.2368
ZINC66496608	-41.3481
ZINC66501977	-39.0084
ZINC66517363	-46.4095
ZINC66517607	-48.2496
ZINC66517891	-40.6898
ZINC66530621	-47.5673
ZINC66532037	-40.2662
ZINC66532037	-40.4514
ZINC66544411	-45.6631
ZINC66552133	-33.4105
ZINC66552822	-41.6164
ZINC66554163	-43.915
ZINC66561448	-35.3571
ZINC66568845	-37.0809
ZINC66569147	-37.0358
ZINC66572323	-34.9191
ZINC66578337	-40.5737
ZINC66578337	-42.7487
ZINC66636987	-45.0382
ZINC66639040	-46.2782
ZINC66648670	-44.648
ZINC66670302	-31.8509
ZINC66675658	-34.7258
ZINC66677218	-40.4485
ZINC66679125	-40.2353
ZINC66679125	-44.4346
ZINC66679229	-39.2981
ZINC66679229	-42.7509
ZINC66679354	-45.06

ZINC66679354	-45.1014
ZINC66679761	-33.8893
ZINC66679761	-40.6956
ZINC66679847	-39.5843
ZINC66679847	-43.2451
ZINC66687124	-44.6723
ZINC66695390	-48.8803
ZINC66696358	-31.2175
ZINC66697795	-44.0173
ZINC66706957	-35.9332
ZINC66708197	-42.6999
ZINC66708197	-43.4066
ZINC66717862	-48.5999
ZINC66737523	-40.2445
ZINC66737638	-29.6122
ZINC66737877	-36.3994
ZINC66741886	-43.1256
ZINC66773432	-38.2763
ZINC66786388	-34.7555
ZINC66786507	-37.6948
ZINC66786775	-38.7742
ZINC66787171	-44.152
ZINC66787245	-38.0563
ZINC66795228	-42.8634
ZINC66795848	-36.9588
ZINC66795848	-43.2613
ZINC66798361	-27.7917
ZINC66802683	-28.8928
ZINC66809039	-50.6733
ZINC66809373	-38.9851
ZINC66810876	-26.8086
ZINC66810876	-37.1277
ZINC66811580	-45.9817
ZINC66812065	-51.5902
ZINC66812339	-45.4593
ZINC66813020	-36.3448
ZINC66815059	-38.6889
ZINC66831177	-35.7812
ZINC66836088	-39.4003
ZINC66837052	-37.6063
ZINC66842067	-41.7709
ZINC66844830	-31.3149
ZINC66844830	-40.3565
ZINC66845399	-45.2318

ZINC66848410	-46.3297
ZINC66849907	-27.3445
ZINC66854305	-29.2308
ZINC66872745	-41.587
ZINC66872745	-43.2346
ZINC66884437	-33.4083
ZINC66887591	-46.1585
ZINC66887591	-54.4486
ZINC66891635	-30.37
ZINC66894196	-45.3094
ZINC66898188	-40.2041
ZINC66903329	-39.1747
ZINC66913431	-41.8592
ZINC66922043	-25.62
ZINC66951179	-42.0509
ZINC66951936	-48.1789
ZINC66951951	-43.9352
ZINC66957971	-47.9955
ZINC66961284	-44.89
ZINC66967019	-42.3058
ZINC66968977	-34.5519
ZINC66986693	-29.5326
ZINC66986693	-30.6719
ZINC66999268	-42.6135
ZINC67029076	-39.7604
ZINC67052015	-40.7896
ZINC67052015	-45.976
ZINC67071916	-40.1611
ZINC67071916	-46.1915
ZINC67079840	-44.3087
ZINC67079840	-53.8731
ZINC67094353	-28.2383
ZINC67094353	-44.444
ZINC67095272	-31.0796
ZINC67103271	-37.727
ZINC67110371	-45.2405
ZINC67113819	-33.5863
ZINC67118068	-39.4824
ZINC67128143	-35.3296
ZINC67171837	-35.3991
ZINC67171896	-35.596
ZINC67172452	-40.023
ZINC67172452	-45.1154
ZINC67172515	-37.4751

ZINC67172526	-31.0403
ZINC67172526	-36.4821
ZINC67172828	-29.1397
ZINC67172828	-34.6855
ZINC67178690	-34.7865
ZINC67189524	-38.4611
ZINC67189545	-38.1257
ZINC67194112	-33.9233
ZINC67196166	-33.9738
ZINC67202808	-43.6498
ZINC67202808	-45.2165
ZINC67211330	-40.026
ZINC67213872	-45.6328
ZINC67228270	-42.3931
ZINC67230497	-42.8027
ZINC67237703	-40.6224
ZINC67250799	-23.8692
ZINC67250799	-36.4247
ZINC67266105	-44.0152
ZINC67284254	-48.3379
ZINC67285163	-24.2599
ZINC67285163	-35.6147
ZINC67286564	-29.0687
ZINC67286564	-31.1379
ZINC67288015	-36.6021
ZINC67288015	-37.0648
ZINC67288750	-31.6405
ZINC67308300	-45.4851
ZINC67308303	-38.5401
ZINC67311174	-45.5157
ZINC67313540	-41.8958
ZINC67313765	-47.1006
ZINC67319168	-38.3744
ZINC67330146	-44.4477
ZINC67338329	-46.0008
ZINC67339274	-39.7641
ZINC67339320	-46.7599
ZINC67339623	-48.1561
ZINC67340532	-38.345
ZINC67340770	-31.5647
ZINC67343613	-44.8929
ZINC67346375	-35.1768
ZINC67346375	-39.233
ZINC67347449	-45.128

ZINC67348340	-42.134
ZINC67350925	-37.7795
ZINC67370325	-34.8639
ZINC67370325	-43.9968
ZINC67372397	-36.5786
ZINC67380560	-38.2604
ZINC67380560	-43.6817
ZINC67399381	-36.7146
ZINC67399521	-42.7555
ZINC67407063	-43.3311
ZINC67418377	-30.0761
ZINC67427131	-42.5764
ZINC67431898	-35.737
ZINC67431898	-39.6388
ZINC67432849	-37.1638
ZINC67434185	-36.963
ZINC67434185	-37.3831
ZINC67434912	-46.9522
ZINC67434920	-20.1516
ZINC67434920	-32.1905
ZINC67459613	-28.4507
ZINC67484693	-40.2037
ZINC67490147	-48.0453
ZINC67490759	-44.6889
ZINC67509363	-43.4061
ZINC67514234	-37.1428
ZINC67514234	-43.1159
ZINC67526886	-43.1048
ZINC67539183	-40.618
ZINC67546143	-42.6394
ZINC67546143	-44.0018
ZINC67549016	-39.3917
ZINC67573840	-36.6386
ZINC67585461	-44.7012
ZINC67600556	-47.9507
ZINC67633527	-43.9081
ZINC67634957	-48.1038
ZINC67641269	-22.8547
ZINC67641269	-41.6348
ZINC67674975	-31.9634
ZINC67674975	-43.9866
ZINC67675556	-37.6569
ZINC67689646	-41.1469
ZINC67690638	-29.9381

ZINC67690638	-37.8256
ZINC67698037	-42.2005
ZINC67720057	-32.7901
ZINC67721814	-47.8542
ZINC67726372	-36.5801
ZINC67726372	-37.8198
ZINC67727182	-36.3351
ZINC67734774	-40.3438
ZINC67739569	-33.1968
ZINC67741677	-31.3333
ZINC67751774	-45.4056
ZINC67756288	-37.7162
ZINC67756901	-47.8875
ZINC67771001	-29.0587
ZINC67771692	-23.6721
ZINC67771692	-30.4201
ZINC67773599	-42.5254
ZINC67773599	-48.8831
ZINC67774013	-39.5412
ZINC67785927	-33.4808
ZINC67804843	-36.1135
ZINC67816967	-36.1506
ZINC67817955	-29.0937
ZINC67818493	-36.2446
ZINC67840501	-44.4991
ZINC67841908	-45.1719
ZINC67842470	-35.2284
ZINC67842470	-38.5482
ZINC67850122	-44.9981
ZINC67850122	-47.8309
ZINC67855713	-33.8551
ZINC67869112	-38.6907
ZINC67876480	-41.0048
ZINC67882108	-49.9688
ZINC67882397	-37.7312
ZINC67884032	-40.0089
ZINC67884032	-43.0769
ZINC67895211	-39.3201
ZINC67896106	-45.7202
ZINC67896106	-49.8193
ZINC67898805	-45.3864
ZINC67898805	-49.6914
ZINC67898826	-32.2589
ZINC67898826	-38.0193

ZINC67909824	-43.4906
ZINC67937220	-40.4124
ZINC67937893	-43.5147
ZINC67946355	-22.6941
ZINC67946355	-29.5989
ZINC67948716	-42.8625
ZINC67948722	-41.816
ZINC67954796	-39.9791
ZINC67954796	-42.4375
ZINC67956316	-24.6138
ZINC67958981	-44.7883
ZINC67970649	-41.5248
ZINC67971993	-34.0005
ZINC67971993	-41.2728
ZINC67973287	-44.7123
ZINC67983431	-43.1598
ZINC67984987	-38.6375
ZINC67997415	-43.4689
ZINC68006214	-41.5818
ZINC68019089	-39.9368
ZINC68035262	-38.2104
ZINC68042067	-46.7058
ZINC68054031	-41.2762
ZINC68058421	-42.8455
ZINC68089738	-46.8764
ZINC68106375	-44.3056
ZINC68106375	-49.0057
ZINC68107764	-27.499
ZINC68124009	-29.5427
ZINC68125806	-35.3411
ZINC68125806	-40.3231
ZINC68125806	-44.4604
ZINC68136406	-32.6132
ZINC68136406	-33.1686
ZINC68143030	-33.9239
ZINC68144531	-51.1223
ZINC68159655	-27.2332
ZINC68171638	-36.2504
ZINC68201578	-40.6518
ZINC68220218	-40.6668
ZINC68227576	-38.276
ZINC68230422	-40.138
ZINC68255237	-48.2146
ZINC68263699	-43.0554

ZINC68263957	-38.2345
ZINC68263957	-46.4875
ZINC68264215	-43.9734
ZINC68283899	-39.5636
ZINC68293752	-42.6727
ZINC68316669	-35.9402
ZINC68319776	-28.06
ZINC68319937	-37.4785
ZINC68319937	-40.4139
ZINC68323351	-45.4109
ZINC68324027	-38.1932
ZINC68328692	-45.9651
ZINC68350171	-45.6507
ZINC68350171	-49.3881
ZINC68358196	-45.6114
ZINC68388176	-38.4082
ZINC68389318	-42.1543
ZINC68405445	-41.2039
ZINC68410718	-45.5648
ZINC68413697	-41.6173
ZINC68413697	-45.7454
ZINC68435013	-34.3959
ZINC68448929	-40.9574
ZINC68453674	-31.1217
ZINC68453674	-39.4383
ZINC68454866	-42.4974
ZINC68455651	-38.6156
ZINC68456079	-41.2212
ZINC68459805	-37.8917
ZINC68480630	-37.6072
ZINC68510000	-38.6815
ZINC68516114	-40.6916
ZINC68517121	-39.4934
ZINC68518218	-38.623
ZINC68527146	-39.6507
ZINC68527146	-44.3968
ZINC68527400	-43.9018
ZINC68540989	-26.6427
ZINC68549030	-44.2981
ZINC68575031	-32.8896
ZINC68583039	-32.2789
ZINC68584835	-38.8115
ZINC68585156	-31.2186
ZINC68585304	-32.8162

ZINC68585803	-38.1436
ZINC68594783	-39.4826
ZINC68662372	-40.2973
ZINC68680625	-42.3688
ZINC68706666	-36.0985
ZINC68726852	-40.4626
ZINC68736816	-34.7153
ZINC68744209	-40.9274
ZINC68744254	-47.8865
ZINC68753085	-28.1951
ZINC68768426	-38.6483
ZINC68768566	-46.779
ZINC68777782	-40.5965
ZINC68780070	-31.3038
ZINC68784489	-36.1027
ZINC68793440	-42.156
ZINC68799471	-35.2375
ZINC68844675	-43.8255
ZINC68855148	-31.2737
ZINC68864023	-36.1204
ZINC68864023	-45.8969
ZINC68865532	-34.78
ZINC68869086	-37.6393
ZINC68869428	-37.9809
ZINC68869428	-38.3908
ZINC68875631	-34.3705
ZINC68875631	-49.0545
ZINC68883081	-45.3556
ZINC68887550	-47.7601
ZINC68913446	-38.5828
ZINC68936706	-36.0431
ZINC68936771	-40.2981
ZINC68953009	-34.4221
ZINC68966498	-42.3779
ZINC68966498	-44.9992
ZINC68975731	-33.7309
ZINC68979786	-34.9961
ZINC68980664	-45.0524
ZINC68983097	-52.4865
ZINC68990691	-34.678
ZINC69008296	-43.0542
ZINC69008425	-34.0303
ZINC69008425	-39.8525
ZINC69008856	-42.2766

ZINC69008856	-46.0205
ZINC69013560	-38.3959
ZINC69013560	-41.3819
ZINC69022766	-25.3738
ZINC69022766	-26.1127
ZINC69025811	-47.0885
ZINC69027343	-37.17
ZINC69066881	-36.5496
ZINC69066881	-39.1576
ZINC69067031	-37.7312
ZINC69067062	-30.2812
ZINC69067159	-32.7682
ZINC69067159	-33.8106
ZINC69069360	-32.476
ZINC69098647	-34.6811
ZINC69098868	-39.7292
ZINC69103253	-44.2644
ZINC69104092	-35.733
ZINC69104520	-44.6051
ZINC69112819	-38.7148
ZINC69114418	-38.4413
ZINC69114418	-39.6919
ZINC69130715	-34.3671
ZINC69147338	-38.435
ZINC69162734	-42.9952
ZINC69162734	-43.1481
ZINC69163948	-43.7255
ZINC69167178	-29.5158
ZINC69170105	-41.5845
ZINC69170852	-34.1059
ZINC69170852	-41.3209
ZINC69174352	-43.518
ZINC69179639	-45.5401
ZINC69189758	-48.5291
ZINC69212498	-31.9695
ZINC69212502	-25.2439
ZINC69215144	-49.0977
ZINC69217477	-42.0687
ZINC69222864	-39.4193
ZINC69247450	-38.6814
ZINC69251682	-42.3431
ZINC69260684	-39.3819
ZINC69260684	-41.9428
ZINC69277306	-45.7062

ZINC69291928	-35.2378
ZINC69291928	-41.2442
ZINC69296375	-45.292
ZINC69302660	-36.1224
ZINC69315987	-42.5539
ZINC69324633	-46.798
ZINC69343640	-41.9097
ZINC69345489	-37.2449
ZINC69346195	-46.0386
ZINC69347506	-34.4612
ZINC69348891	-34.4536
ZINC69348891	-39.8997
ZINC69362865	-48.2669
ZINC69369000	-35.8708
ZINC69369000	-40.0698
ZINC69383739	-38.5454
ZINC69415962	-41.5186
ZINC69416863	-37.1832
ZINC69430742	-35.9089
ZINC69445114	-36.6767
ZINC69457234	-39.0676
ZINC69464506	-35.782
ZINC69464506	-36.3754
ZINC69483710	-35.9888
ZINC69485623	-42.6216
ZINC69491716	-41.0479
ZINC69508348	-33.507
ZINC69508822	-37.5453
ZINC69510347	-35.004
ZINC69512088	-35.8513
ZINC69517833	-35.3749
ZINC69518014	-26.763
ZINC69527329	-47.2299
ZINC69528257	-30.635
ZINC69528257	-32.8919
ZINC69533567	-37.7267
ZINC69534463	-42.9657
ZINC69535593	-33.122
ZINC69535593	-40.616
ZINC69538683	-44.6396
ZINC69538704	-46.9911
ZINC69539525	-48.146
ZINC69548116	-41.3097
ZINC69548175	-47.55

ZINC69553409	-40.5052
ZINC69554182	-47.4514
ZINC69564488	-38.194
ZINC69564488	-39.3426
ZINC69564648	-32.266
ZINC69565364	-26.7806
ZINC69565364	-33.2034
ZINC69570110	-41.3104
ZINC69591337	-42.3049
ZINC69591337	-45.3579
ZINC69597986	-41.8603
ZINC69612931	-46.7996
ZINC69625116	-48.4139
ZINC69626827	-45.1166
ZINC69641581	-55.2908
ZINC69641816	-46.8949
ZINC69645938	-44.3164
ZINC69647937	-49.9184
ZINC69647937	-51.7466
ZINC69652129	-46.4664
ZINC69654251	-34.844
ZINC69654544	-32.6359
ZINC69654686	-35.6904
ZINC69656618	-36.6141
ZINC69663471	-50.9677
ZINC69671933	-38.47
ZINC69675076	-33.7015
ZINC69676871	-33.1445
ZINC69676871	-36.9879
ZINC69703788	-47.5893
ZINC69704816	-41.3767
ZINC69706930	-41.1151
ZINC69709975	-35.3601
ZINC69709975	-43.6464
ZINC69731325	-41.9901
ZINC69737083	-41.9108
ZINC69739093	-30.8237
ZINC69739254	-39.8288
ZINC69739254	-42.7514
ZINC69740479	-35.1866
ZINC69740479	-43.9856
ZINC69745531	-40.2125
ZINC69745531	-47.7505
ZINC69749317	-40.0792

ZINC69770355	-45.0143
ZINC69770355	-46.3158
ZINC69771103	-38.8178
ZINC69771103	-38.9879
ZINC69775733	-39.2842
ZINC69775733	-42.7601
ZINC69780189	-32.0107
ZINC69780189	-39.65
ZINC69796595	-28.9446
ZINC69796595	-32.3362
ZINC69803930	-23.1656
ZINC69803930	-33.1526
ZINC69806123	-34.0568
ZINC69806332	-42.4086
ZINC69806532	-39.4645
ZINC69809017	-37.9526
ZINC69810091	-36.1777
ZINC69810933	-52.7684
ZINC69811155	-41.7723
ZINC69812057	-36.4174
ZINC69812057	-40.1914
ZINC69813147	-33.0074
ZINC69818161	-37.4962
ZINC69821644	-36.7593
ZINC69842435	-36.5131
ZINC69842446	-43.2793
ZINC69844039	-42.7137
ZINC69844623	-40.5717
ZINC69848532	-46.0245
ZINC69868340	-28.3105
ZINC69868682	-31.1745
ZINC69868682	-41.2498
ZINC69871684	-42.0038
ZINC69871684	-44.8484
ZINC69871813	-37.8954
ZINC69871813	-42.2725
ZINC69871813	-43.9708
ZINC69878021	-37.2054
ZINC69878021	-39.8215
ZINC69895318	-36.6355
ZINC69900443	-41.4593
ZINC69901076	-50.1304
ZINC69924504	-42.3616
ZINC69928565	-28.3509

ZINC69928565	-34.9289
ZINC69935992	-44.2515
ZINC69949848	-41.7853
ZINC69949981	-44.1301
ZINC69952963	-46.6799
ZINC69971596	-31.0121
ZINC69971596	-37.6515
ZINC69972031	-37.8781
ZINC69975510	-31.6684
ZINC69979831	-42.0787
ZINC69980388	-35.7837
ZINC69980388	-39.3967
ZINC69991748	-53.7179
ZINC70016263	-37.3098
ZINC70032374	-27.9448
ZINC70032483	-36.4021
ZINC70036721	-48.3336
ZINC70039607	-38.8354
ZINC70047521	-40.4706
ZINC70431759	-36.6964
ZINC70451514	-32.63
ZINC70451514	-36.1632
ZINC70452100	-33.4233
ZINC70453031	-43.683
ZINC70453412	-26.3106
ZINC70456128	-34.1284
ZINC70460339	-42.2101
ZINC70460774	-36.6443
ZINC70638712	-33.0953
ZINC70638712	-33.4758
ZINC70668280	-45.2045
ZINC70695717	-50.1181
ZINC70696863	-32.1768
ZINC70706741	-45.8956
ZINC70718350	-39.8779
ZINC70753259	-46.6737
ZINC70753866	-50.259
ZINC70754902	-34.6367
ZINC70754902	-38.2492
ZINC70765618	-39.2174
ZINC70772316	-35.9446
ZINC70782082	-40.0935
ZINC70786721	-38.2117
ZINC70786721	-39.3464

ZINC70790874	-34.096
ZINC70790874	-37.3152
ZINC70807683	-35.9981
ZINC70807918	-34.517
ZINC70808816	-44.5945
ZINC70813929	-47.2393
ZINC70814405	-35.0923
ZINC70814872	-39.2874
ZINC70838767	-39.7021
ZINC70839648	-42.764
ZINC70854214	-35.2911
ZINC70856219	-40.3371
ZINC70857185	-33.0441
ZINC70865100	-46.5783
ZINC70867478	-38.4506
ZINC70867848	-40.0698
ZINC70876423	-28.3036
ZINC70881902	-42.7986
ZINC70896731	-49.4216
ZINC70905534	-44.1125
ZINC70926553	-42.9151
ZINC70928991	-45.537
ZINC70929332	-42.7781
ZINC70929686	-42.5268
ZINC70932140	-39.1164
ZINC70933767	-41.6855
ZINC70997531	-44.3713
ZINC71016262	-38.6292
ZINC71019267	-28.5619
ZINC71024563	-41.5666
ZINC71047566	-44.0581
ZINC71075723	-30.954
ZINC71084535	-29.8854
ZINC71089180	-33.35
ZINC71089180	-33.4284
ZINC71089538	-41.8606
ZINC71089538	-42.0986
ZINC71089739	-37.8666
ZINC71089739	-40.4957
ZINC71089760	-46.4942
ZINC71122426	-41.1399
ZINC71185324	-46.9229
ZINC71185866	-36.3631
ZINC71195552	-43.3772

ZINC71195855	-38.2587
ZINC71236284	-39.3055
ZINC71250665	-27.7471
ZINC71280160	-46.4559
ZINC71284690	-28.0267
ZINC71285705	-35.0705
ZINC71289152	-28.6457
ZINC71315550	-24.7566
ZINC71315550	-28.8883
ZINC71388542	-36.2565
ZINC71388794	-44.4768
ZINC71405190	-48.1051
ZINC71422102	-46.8992
ZINC71439928	-29.1733
ZINC71614381	-43.8872
ZINC71614628	-35.9977
ZINC71614628	-45.4678
ZINC71618680	-33.3062
ZINC71621105	-27.5796
ZINC71623904	-33.5575
ZINC71624524	-28.9026
ZINC71625026	-31.9777
ZINC71707833	-42.7922
ZINC71711950	-34.2882
ZINC71715307	-46.7072
ZINC71716019	-35.3958
ZINC71738467	-34.1283
ZINC71738467	-35.1327
ZINC71738722	-36.8721
ZINC71745967	-23.7251
ZINC71745967	-30.3122
ZINC71746283	-27.1972
ZINC71746283	-29.2836
ZINC71747559	-33.1316
ZINC71747559	-40.561
ZINC71751421	-29.6882
ZINC71752226	-25.3325
ZINC71753963	-45.5539
ZINC71756835	-34.4567
ZINC71758799	-42.5362
ZINC71760812	-48.5475
ZINC71761793	-38.025
ZINC71762918	-40.6817
ZINC71763882	-39.4411

ZINC71763882	-43.5501
ZINC71764168	-40.7693
ZINC71764740	-28.5508
ZINC71764928	-34.6803
ZINC71767424	-32.0449
ZINC71768770	-42.6166
ZINC71776236	-39.6555
ZINC71779412	-36.5577
ZINC71779802	-34.6532
ZINC71780773	-30.8651
ZINC71781059	-33.8558
ZINC71782402	-46.078
ZINC71782435	-29.3185
ZINC71782435	-31.2119
ZINC71785275	-38.2234
ZINC71788283	-38.6508
ZINC71788283	-40.7772
ZINC71790483	-27.7121
ZINC71791341	-38.1912
ZINC71792562	-43.2243
ZINC71796283	-46.6224
ZINC71801364	-32.9405
ZINC71801364	-42.5808
ZINC71803785	-30.0819
ZINC71804504	-37.295
ZINC71806758	-40.0263
ZINC71808750	-39.57
ZINC71812712	-34.2269
ZINC71815413	-48.1407
ZINC71817165	-24.5193
ZINC71818836	-35.0525
ZINC71821454	-44.0089
ZINC71821455	-47.9158
ZINC71822306	-43.9679
ZINC71822583	-33.0722
ZINC71822583	-40.1456
ZINC71823792	-40.6109
ZINC71824132	-29.717
ZINC71825151	-37.6195
ZINC71825497	-38.7869
ZINC71825497	-41.1638
ZINC71825819	-38.682
ZINC71825819	-44.1591
ZINC71827767	-31.3036

ZINC71834702	-45.1346
ZINC71840815	-39.2158
ZINC71840815	-39.7641
ZINC71851621	-33.2014
ZINC71852848	-41.0921
ZINC71854700	-30.7044
ZINC71854733	-35.9697
ZINC71860584	-38.6194
ZINC71864778	-29.1544
ZINC71864778	-36.934
ZINC71867019	-40.1462
ZINC71868107	-38.5473
ZINC71869756	-37.5231
ZINC71870192	-39.7243
ZINC71870408	-31.9656
ZINC71876085	-33.7821
ZINC71877530	-35.0472
ZINC71881937	-40.0465
ZINC71887787	-37.643
ZINC71887787	-49.8199
ZINC71888014	-39.4083
ZINC71888922	-40.9347
ZINC71888922	-41.0761
ZINC71893313	-37.4653
ZINC71893313	-42.0486
ZINC71893643	-32.6879
ZINC71897349	-34.7323
ZINC71900609	-35.7229
ZINC71902822	-38.4464
ZINC71903334	-40.3505
ZINC71903564	-38.3129
ZINC71904340	-29.1796
ZINC71904610	-34.6041
ZINC71907119	-23.0112
ZINC71907729	-35.0591
ZINC71908767	-37.8778
ZINC71908783	-41.1633
ZINC71909095	-42.0406
ZINC71910917	-47.5087
ZINC71911017	-32.0016
ZINC71913992	-41.327
ZINC71913992	-42.4635
ZINC71914133	-41.942
ZINC71914133	-42.0213

ZINC71914375	-41.6515
ZINC71915094	-35.0952
ZINC71916386	-42.1749
ZINC71921522	-34.0308
ZINC71921522	-35.0272
ZINC71922025	-25.354
ZINC71930737	-45.5014
ZINC71932822	-29.008
ZINC71933098	-34.731
ZINC71934015	-39.9896
ZINC71937868	-50.2312
ZINC71946763	-39.1674
ZINC71951458	-36.738
ZINC71951458	-41.5195
ZINC71952241	-29.4877
ZINC71952367	-42.5194
ZINC71961421	-26.6398
ZINC71963526	-48.518
ZINC71966693	-32.4175
ZINC71968498	-46.9029
ZINC71975168	-30.762
ZINC71977052	-36.2329
ZINC71977198	-31.973
ZINC71977850	-31.9843
ZINC71977850	-36.0299
ZINC71982975	-44.6554
ZINC72002648	-29.3596
ZINC72012154	-46.8825
ZINC72016365	-31.6227
ZINC72017921	-31.448
ZINC72020901	-44.7431
ZINC72023774	-31.0957
ZINC72024398	-26.1665
ZINC72037034	-41.5941
ZINC72042606	-32.2266
ZINC72042606	-33.7822
ZINC72049628	-31.0661
ZINC72055182	-41.7447
ZINC72057058	-35.5216
ZINC72057301	-30.2295
ZINC72061135	-31.5778
ZINC72063720	-27.5859
ZINC72065983	-36.424
ZINC72074260	-43.3133

ZINC72080200	-46.8244
ZINC72080839	-45.2972
ZINC72080961	-51.0122
ZINC72081068	-31.9556
ZINC72081331	-28.779
ZINC72086695	-39.1354
ZINC72086740	-45.9204
ZINC72087162	-32.5491
ZINC72094760	-44.1733
ZINC72096472	-33.7374
ZINC72109564	-38.9483
ZINC72117630	-30.0362
ZINC72117630	-35.4774
ZINC72119130	-36.117
ZINC72119483	-37.8813
ZINC72133027	-34.5324
ZINC72135497	-34.0215
ZINC72139593	-25.3489
ZINC72139593	-25.6696
ZINC72140163	-33.4185
ZINC72140163	-35.9829
ZINC72140356	-34.0806
ZINC72142004	-50.3536
ZINC72144505	-40.4031
ZINC72144730	-35.9781
ZINC72144730	-43.9234
ZINC72148540	-45.3618
ZINC72148871	-33.6307
ZINC72148871	-43.3775
ZINC72149382	-33.1807
ZINC72149382	-41.8635
ZINC72150549	-43.7327
ZINC72150609	-45.5728
ZINC72157466	-43.5966
ZINC72160928	-36.3746
ZINC72161766	-46.223
ZINC72163587	-38.9311
ZINC72163587	-54.7408
ZINC72167403	-37.0702
ZINC72169109	-36.7791
ZINC72169287	-31.0857
ZINC72170029	-43.9374
ZINC72174294	-28.49
ZINC72187695	-33.2538

ZINC72188047	-25.0821
ZINC72190453	-29.3754
ZINC72190455	-31.0514
ZINC72192521	-29.3807
ZINC72193053	-29.3802
ZINC72193080	-29.0134
ZINC72193087	-32.0015
ZINC72195777	-30.3738
ZINC72195778	-30.222
ZINC72195828	-33.1014
ZINC72196576	-37.0039
ZINC72196916	-36.3085
ZINC72197017	-33.8443
ZINC72197356	-26.8097
ZINC72197891	-30.2193
ZINC72197891	-36.3653
ZINC72197965	-33.4199
ZINC72198871	-30.6774
ZINC72198871	-35.0211
ZINC72201923	-34.4894
ZINC72203532	-42.1844
ZINC72231971	-42.9295
ZINC72232611	-43.5592
ZINC72232611	-45.865
ZINC72233293	-30.1246
ZINC72233741	-41.3849
ZINC72233818	-35.4513
ZINC72236419	-48.3146
ZINC72237976	-33.5063
ZINC72242111	-42.0985
ZINC72243267	-38.492
ZINC72246025	-35.3688
ZINC72247396	-41.7804
ZINC72253743	-39.892
ZINC72253743	-43.3858
ZINC72255153	-31.9923
ZINC72255153	-35.4056
ZINC72255434	-30.6842
ZINC72255812	-47.6092
ZINC72255812	-51.3642
ZINC72263100	-42.4359
ZINC72263100	-44.3994
ZINC72264456	-34.9372
ZINC72264955	-46.0692

ZINC72268418	-40.1534
ZINC72268418	-43.5021
ZINC72274909	-47.1813
ZINC72275651	-42.4131
ZINC72275662	-42.8741
ZINC72275662	-47.0837
ZINC72276197	-39.4718
ZINC72276790	-38.4623
ZINC72276999	-45.8367
ZINC72277009	-40.4211
ZINC72278633	-44.7842
ZINC72281674	-27.5658
ZINC72283506	-38.2432
ZINC72284537	-29.6099
ZINC72285316	-42.3794
ZINC72285527	-40.6591
ZINC72287296	-42.8286
ZINC72291696	-36.6716
ZINC72291784	-40.4146
ZINC72291988	-36.7413
ZINC72294093	-44.3033
ZINC72294093	-44.3239
ZINC72301028	-47.4967
ZINC72302357	-36.2432
ZINC72302357	-38.9417
ZINC72302951	-43.447
ZINC72304224	-43.924
ZINC72304327	-38.157
ZINC72304327	-38.5257
ZINC72305202	-42.3939
ZINC72306152	-44.0767
ZINC72306152	-46.879
ZINC72310193	-34.7611
ZINC72310715	-41.4816
ZINC72318932	-38.7478
ZINC72320311	-30.774
ZINC72320651	-26.9182
ZINC72322100	-28.1711
ZINC72322100	-44.476
ZINC72324764	-29.6955
ZINC72324764	-31.0199
ZINC72334969	-32.6191
ZINC72335336	-43.8813
ZINC72335351	-41.746

ZINC72335617	-43.3989
ZINC72335617	-45.4605
ZINC72335853	-33.2504
ZINC72337085	-48.7267
ZINC72339273	-30.2106
ZINC72339648	-35.5815
ZINC72341609	-39.2495
ZINC72342496	-32.1614
ZINC72344201	-35.1471
ZINC72347817	-44.9203
ZINC72348687	-38.4612
ZINC72349612	-38.4119
ZINC72350229	-35.5536
ZINC72350875	-45.8407
ZINC72355565	-28.7907
ZINC72357688	-27.0478
ZINC72357688	-33.5832
ZINC72357886	-39.1263
ZINC72358621	-29.2056
ZINC72358621	-36.9051
ZINC72362180	-46.3535
ZINC72362521	-46.6567
ZINC72363141	-43.2044
ZINC72363221	-32.0647
ZINC72363221	-38.4612
ZINC72365750	-36.7895
ZINC72367940	-37.394
ZINC72368388	-42.589
ZINC72369640	-31.3465
ZINC72373130	-40.1199
ZINC72375951	-32.1272
ZINC72377162	-29.522
ZINC72379352	-38.3638
ZINC72380353	-31.8263
ZINC72380353	-36.3991
ZINC72380646	-54.3644
ZINC72380646	-55.674
ZINC72385864	-37.776
ZINC72389649	-32.3485
ZINC72389649	-37.5384
ZINC72391399	-40.0687
ZINC72392937	-33.9825
ZINC72392937	-45.4419
ZINC72393734	-33.8829

ZINC72397541	-31.4581
ZINC72397541	-31.6838
ZINC72397870	-37.5817
ZINC72398109	-40.0322
ZINC72398116	-29.902
ZINC72402440	-41.7339
ZINC72404206	-37.131
ZINC72404206	-37.715
ZINC72405035	-27.4167
ZINC72408184	-33.6836
ZINC72408421	-39.7617
ZINC72409021	-34.1255
ZINC72410104	-29.7012
ZINC72410531	-34.9105
ZINC72411281	-46.3078
ZINC72412399	-37.9844
ZINC72412399	-40.3214
ZINC72414684	-45.665
ZINC72415385	-32.0859
ZINC72415385	-33.0255
ZINC72417905	-29.4362
ZINC72418590	-42.5739
ZINC72420541	-42.2184
ZINC72421924	-32.9761
ZINC72423037	-36.0364
ZINC72423520	-41.2194
ZINC72423654	-38.9659
ZINC72427368	-34.1984
ZINC72432469	-33.0081
ZINC72432469	-36.9944
ZINC72435508	-38.769
ZINC72435847	-34.0371
ZINC72435847	-36.1566
ZINC72438149	-33.0738
ZINC72438783	-42.8515
ZINC72443346	-32.9177
ZINC72443350	-34.0336
ZINC72443929	-40.3508
ZINC72449070	-35.8545
ZINC72455413	-36.7942
ZINC72455907	-45.7705
ZINC72457942	-30.8774
ZINC72459712	-23.9064
ZINC72459752	-26.0738

ZINC72467393	-34.6285
ZINC72467539	-44.1491
ZINC72469270	-34.1351
ZINC72470667	-38.5809
ZINC72470667	-41.9481
ZINC72470860	-43.9458
ZINC72470860	-44.6031
ZINC72473236	-39.3654
ZINC72474964	-44.3634
ZINC72475889	-46.4612
ZINC72479160	-33.3935
ZINC72481243	-24.6326
ZINC72481328	-36.5011
ZINC72481328	-40.9518
ZINC72483485	-36.1336
ZINC72487517	-39.2284
ZINC72487553	-39.6866
ZINC72488848	-22.491
ZINC72489991	-38.8358
ZINC72499353	-39.7087
ZINC72508772	-44.6591
ZINC72509173	-39.1081
ZINC72513958	-38.6281
ZINC72513958	-41.6003
ZINC72514086	-31.7375
ZINC72514086	-32.5381
ZINC72518377	-38.9471
ZINC72518377	-43.8832
ZINC72529562	-40.7168
ZINC72533591	-31.6728
ZINC72538424	-34.2984
ZINC72539348	-46.1182
ZINC72539362	-47.6613
ZINC72546667	-35.3261
ZINC72557580	-37.5151
ZINC72557580	-39.2559
ZINC72562462	-34.5748
ZINC72573937	-43.6207
ZINC72575308	-41.2104
ZINC72575320	-38.0244
ZINC72578304	-35.4138
ZINC72578304	-39.9786
ZINC72578320	-43.2707
ZINC72578320	-43.9041

ZINC72591878	-36.8217
ZINC72596186	-48.9133
ZINC72596612	-37.2752
ZINC72605407	-40.568
ZINC72614742	-38.3654
ZINC72622689	-44.7942
ZINC72632646	-38.0751
ZINC72633391	-30.8144
ZINC72639997	-40.9749
ZINC72647119	-47.9461
ZINC72649815	-33.6515
ZINC72652123	-48.3215
ZINC72661551	-40.983
ZINC72663152	-37.4073
ZINC72665785	-33.8252
ZINC72665785	-39.8321
ZINC72670171	-39.1645
ZINC72670171	-40.5683
ZINC72672816	-42.852
ZINC72677537	-36.9854
ZINC72684361	-40.8089
ZINC72684361	-43.0894
ZINC72685075	-32.5882
ZINC72685075	-40.2759
ZINC72686458	-39.197
ZINC72690429	-45.3313
ZINC72692141	-40.0394
ZINC72697944	-35.8172
ZINC72698771	-36.6628
ZINC72698771	-36.9526
ZINC72705508	-40.4709
ZINC72707160	-38.8619
ZINC72725763	-39.3477
ZINC72727445	-30.9205
ZINC72730215	-36.86
ZINC72730371	-41.241
ZINC72733440	-40.2819
ZINC72739518	-41.6415
ZINC72741074	-44.4223
ZINC72742306	-41.1553
ZINC72745017	-42.1083
ZINC72748029	-37.49
ZINC72760046	-47.027
ZINC72760046	-47.367

ZINC72767186	-28.0791
ZINC72774171	-33.0685
ZINC72774171	-37.1931
ZINC72774933	-35.2315
ZINC72776506	-29.0595
ZINC72778788	-41.4635
ZINC72793345	-42.8582
ZINC72794844	-32.8378
ZINC72795144	-24.2325
ZINC72795144	-37.6201
ZINC72795719	-37.1865
ZINC72795719	-42.6003
ZINC72797358	-41.5249
ZINC72797814	-35.4418
ZINC72800012	-27.2626
ZINC72802453	-45.0717
ZINC72802453	-48.2715
ZINC72802516	-41.8661
ZINC72802984	-37.7795
ZINC72802984	-38.509
ZINC72805476	-36.9073
ZINC72806173	-42.6537
ZINC72806922	-37.778
ZINC72810741	-36.9036
ZINC72812465	-34.9521
ZINC72813784	-38.4883
ZINC72813952	-38.1373
ZINC72816456	-44.2793
ZINC72816456	-50.3374
ZINC72817366	-36.4216
ZINC72817377	-36.1795
ZINC72817597	-43.7773
ZINC72817597	-47.7273
ZINC72818073	-45.2393
ZINC72824636	-45.8543
ZINC72824688	-44.4576
ZINC72825413	-39.8856
ZINC72826909	-38.6671
ZINC72832460	-32.0289
ZINC72832460	-39.5062
ZINC72833591	-35.3542
ZINC72833591	-39.5363
ZINC72834786	-41.6261
ZINC72838301	-40.5566

ZINC72838786	-43.0698
ZINC72841954	-41.8611
ZINC72844385	-45.2295
ZINC72844385	-49.2788
ZINC72844389	-37.4441
ZINC72845100	-41.3064
ZINC72846922	-35.1105
ZINC72847137	-38.2222
ZINC72847360	-32.8881
ZINC72852156	-39.5502
ZINC72856486	-41.8477
ZINC72858151	-42.7784
ZINC72858151	-42.9315
ZINC72867344	-35.4451
ZINC72867344	-50.0356
ZINC72877902	-44.7612
ZINC72887514	-49.2255
ZINC72894210	-35.7456
ZINC72894661	-37.426
ZINC72895494	-30.5804
ZINC72895494	-35.9104
ZINC72895839	-41.9386
ZINC72896846	-37.1643
ZINC72899460	-33.3335
ZINC72899460	-37.3646
ZINC72900185	-36.2941
ZINC72900197	-42.6735
ZINC72908734	-41.4696
ZINC72908734	-42.074
ZINC72909016	-40.2589
ZINC72909972	-32.2922
ZINC72910028	-39.3889
ZINC72912717	-41.2312
ZINC72915480	-43.9685
ZINC72917300	-41.7061
ZINC72917905	-42.4012
ZINC72918852	-29.3439
ZINC72925169	-38.79
ZINC72926014	-46.0459
ZINC72926014	-48.8722
ZINC72932088	-39.2852
ZINC72945728	-37.8999
ZINC72948396	-28.1426
ZINC72948396	-35.9269

ZINC72952746	-37.4801
ZINC72952746	-40.1811
ZINC72966155	-32.3412
ZINC72966155	-39.7455
ZINC72966954	-39.065
ZINC72968567	-40.3065
ZINC72980101	-36.9725
ZINC72983124	-36.7093
ZINC72983124	-36.777
ZINC72984106	-38.0487
ZINC72986714	-40.5331
ZINC72989606	-45.845
ZINC72990619	-36.5106
ZINC72997355	-41.0159
ZINC72997474	-46.0077
ZINC72997948	-34.9479
ZINC73016142	-39.0459
ZINC73017584	-40.1898
ZINC73021336	-36.9935
ZINC73024065	-36.923
ZINC73028051	-38.1745
ZINC73028051	-40.4066
ZINC73031875	-42.6297
ZINC73035646	-46.511
ZINC73048064	-45.2078
ZINC73052818	-38.6302
ZINC73058753	-48.6951
ZINC73063900	-41.1829
ZINC73066389	-37.3685
ZINC73075526	-36.6471
ZINC73076694	-44.3438
ZINC73085070	-37.5737
ZINC73085548	-45.8658
ZINC73086209	-38.8489
ZINC73087359	-50.5204
ZINC73089686	-41.7453
ZINC73096445	-45.49
ZINC73098472	-43.1511
ZINC73099272	-45.7099
ZINC73103046	-44.9179
ZINC73104202	-35.4969
ZINC73104202	-38.4055
ZINC73106915	-32.9615
ZINC73106915	-36.6004

ZINC73110614	-35.1828
ZINC73115177	-43.0162
ZINC73122341	-39.5638
ZINC73122552	-38.0377
ZINC73123248	-42.0143
ZINC73123932	-34.8022
ZINC73124232	-40.9413
ZINC73124410	-42.1211
ZINC73124866	-33.7305
ZINC73124866	-38.1005
ZINC73125075	-41.3659
ZINC73125075	-47.4151
ZINC73131611	-36.337
ZINC73132897	-34.5403
ZINC73132897	-37.5865
ZINC73139132	-38.8708
ZINC73139334	-46.3064
ZINC73163427	-39.1996
ZINC73165145	-43.5382
ZINC73165321	-33.6276
ZINC73165321	-38.7424
ZINC73167212	-38.2613
ZINC73171348	-40.9949
ZINC73179311	-39.441
ZINC73186107	-34.7215
ZINC73186440	-36.1449
ZINC73186477	-39.9462
ZINC73186707	-38.234
ZINC73193960	-43.6651
ZINC73195383	-40.5567
ZINC73195531	-40.8459
ZINC73195531	-42.9513
ZINC73202997	-33.5043
ZINC73221423	-43.2832
ZINC73227124	-40.0337
ZINC73233786	-38.8924
ZINC73235697	-42.3201
ZINC73235697	-42.5168
ZINC73244920	-42.1581
ZINC73250935	-40.5579
ZINC73251224	-34.8322
ZINC73256859	-37.0983
ZINC73269267	-40.9054
ZINC73274975	-35.9721

ZINC73274975	-40.6574
ZINC73284358	-44.4903
ZINC73284358	-46.1934
ZINC73287011	-40.0507
ZINC73291464	-39.9378
ZINC73291464	-41.1799
ZINC73292750	-41.1597
ZINC73293926	-40.7356
ZINC73302771	-37.2187
ZINC73303253	-34.5465
ZINC73309845	-36.9663
ZINC73310142	-41.1531
ZINC73314181	-29.3347
ZINC73319615	-40.1299
ZINC73325748	-29.3926
ZINC73327563	-38.0092
ZINC73332129	-41.1693
ZINC73332501	-36.5249
ZINC73338623	-37.199
ZINC73338623	-42.0951
ZINC73342199	-41.4617
ZINC73342199	-44.5355
ZINC73344235	-39.0246
ZINC73346684	-39.1833
ZINC73347021	-41.1341
ZINC73348461	-42.1378
ZINC73349388	-38.6726
ZINC73351242	-34.6541
ZINC73351592	-40.5058
ZINC73352149	-38.6894
ZINC73353051	-42.8148
ZINC73358666	-37.2403
ZINC73358666	-40.1237
ZINC73358684	-38.781
ZINC73363322	-35.9761
ZINC73363947	-41.5965
ZINC73364510	-36.3705
ZINC73364510	-37.6641
ZINC73374441	-44.5254
ZINC73374781	-41.9422
ZINC73381673	-42.4497
ZINC73381903	-36.4076
ZINC73381962	-46.3575
ZINC73381962	-48.8211

ZINC73390077	-38.767
ZINC73398758	-38.0496
ZINC73405215	-39.7353
ZINC73420618	-40.0145
ZINC73431195	-45.5518
ZINC73432572	-44.2948
ZINC73433205	-41.768
ZINC73458086	-42.145
ZINC73462695	-35.6988
ZINC73463183	-42.2693
ZINC73463299	-34.8182
ZINC73463299	-37.6614
ZINC73469950	-36.8757
ZINC73469950	-38.7012
ZINC73471202	-41.7213
ZINC73473868	-37.1152
ZINC73474879	-41.6556
ZINC73479057	-37.1005
ZINC73479057	-38.4657
ZINC73489572	-41.9241
ZINC73490113	-33.7458
ZINC73490456	-34.9954
ZINC73490456	-36.7452
ZINC73495808	-47.3146
ZINC73496195	-30.3641
ZINC73498180	-33.7572
ZINC73498180	-41.9511
ZINC73503737	-35.8423
ZINC73507134	-30.9354
ZINC73512804	-38.3443
ZINC73516135	-39.0882
ZINC73517334	-35.3746
ZINC73518748	-39.1538
ZINC73519192	-41.1832
ZINC73523983	-33.8608
ZINC73524900	-37.048
ZINC73524900	-46.9396
ZINC73526135	-38.3119
ZINC73530102	-45.3192
ZINC73532402	-39.1462
ZINC73532402	-42.6288
ZINC73534088	-39.307
ZINC73534088	-40.9943
ZINC73540780	-33.8846

ZINC73544516	-42.2847
ZINC73544793	-42.9675
ZINC73550040	-38.6022
ZINC73553644	-34.3618
ZINC73566686	-37.0818
ZINC73566696	-39.2764
ZINC73566696	-43.1108
ZINC73585161	-35.1424
ZINC73601331	-39.1311
ZINC73601436	-38.4129
ZINC73602872	-30.731
ZINC73603186	-42.5186
ZINC73603448	-38.8715
ZINC73604776	-44.1786
ZINC73606033	-33.4157
ZINC73612836	-35.9582
ZINC73624720	-37.7615
ZINC73640314	-35.9043
ZINC73641934	-42.813
ZINC73646591	-40.3122
ZINC73646591	-42.6915
ZINC73647917	-38.2076
ZINC73648014	-44.2257
ZINC73649670	-38.9483
ZINC73665210	-42.7664
ZINC73665545	-26.8242
ZINC73667416	-37.9154
ZINC73668257	-40.121
ZINC73677241	-37.9965
ZINC73678649	-35.1903
ZINC73678701	-33.8313
ZINC73680949	-52.0146
ZINC73682132	-37.9574
ZINC73682623	-36.9095
ZINC73682623	-47.8686
ZINC73687058	-44.5389
ZINC73690608	-43.5293
ZINC73694595	-43.432
ZINC73696213	-39.6046
ZINC73696367	-37.9376
ZINC73709541	-43.3109
ZINC73709541	-48.8212
ZINC73710608	-39.3448
ZINC73710608	-49.763

ZINC73717730	-41.9605
ZINC73719372	-37.28
ZINC73731510	-32.5483
ZINC73731723	-44.1675
ZINC73739130	-36.8332
ZINC73739624	-32.7277
ZINC73739624	-37.5912
ZINC73741477	-35.0762
ZINC73746998	-35.8452
ZINC73749141	-45.6676
ZINC73750261	-46.0435
ZINC73751000	-43.9153
ZINC73757193	-43.7529
ZINC73757514	-40.09
ZINC73763666	-37.0716
ZINC73766779	-37.728
ZINC73770402	-42.2505
ZINC73771012	-36.0776
ZINC73772089	-40.4375
ZINC73775191	-43.6681
ZINC73775322	-38.1254
ZINC73775489	-41.7357
ZINC73775715	-43.2282
ZINC73778864	-40.7006
ZINC73784640	-35.0569
ZINC73790399	-40.7337
ZINC73790404	-32.2069
ZINC73791364	-48.9561
ZINC73802095	-46.6153
ZINC73823507	-32.0562
ZINC73827976	-46.3751
ZINC73828224	-34.2645
ZINC73829755	-43.4525
ZINC73835932	-32.4892
ZINC73837161	-41.8193
ZINC73848230	-39.8384
ZINC73848424	-38.5661
ZINC73851558	-41.1643
ZINC73852103	-39.4222
ZINC73852968	-35.1303
ZINC73857958	-46.9805
ZINC73865952	-53.5112
ZINC73871941	-33.9167
ZINC73880054	-43.8864

ZINC73880054	-43.9843
ZINC73880815	-43.2963
ZINC73887622	-36.3288
ZINC73890942	-37.976
ZINC73892196	-41.3761
ZINC73900703	-47.3992
ZINC73900813	-39.6021
ZINC73901025	-36.1888
ZINC73902119	-45.1395
ZINC73909930	-39.0539
ZINC73920704	-37.0345
ZINC73920704	-39.3245
ZINC73922181	-39.6543
ZINC73922181	-45.737
ZINC73922314	-38.8793
ZINC73937708	-41.9522
ZINC73937708	-43.2627
ZINC73938108	-40.0314
ZINC73938109	-37.1517
ZINC73950274	-35.3954
ZINC73950274	-49.9627
ZINC73952073	-37.846
ZINC73952831	-45.8269
ZINC73953124	-46.7173
ZINC73954957	-39.2329
ZINC73957439	-37.8666
ZINC73957439	-41.8223
ZINC73967331	-41.3686
ZINC73973089	-43.5126
ZINC73973089	-57.1056
ZINC73987354	-38.8394
ZINC73992980	-35.8397
ZINC73993555	-46.9065
ZINC73998586	-41.5644
ZINC73999035	-39.5065
ZINC74008289	-40.8603
ZINC74017248	-45.3978
ZINC74022334	-37.6126
ZINC74049929	-38.2959
ZINC74054058	-46.1041
ZINC74055248	-37.7308
ZINC74057727	-33.8024
ZINC74059201	-31.4623
ZINC74060957	-37.1993

ZINC74061896	-42.2396
ZINC74062089	-38.3582
ZINC74062599	-44.4083
ZINC74064516	-45.2884
ZINC74068763	-50.4955
ZINC74072823	-44.946
ZINC74074382	-43.0089
ZINC74087590	-39.7753
ZINC74087590	-42.8634
ZINC74098118	-36.4587
ZINC74099654	-32.1496
ZINC74104814	-43.8452
ZINC74104814	-46.2926
ZINC74106077	-41.6065
ZINC74107982	-41.5495
ZINC74107982	-43.879
ZINC74125465	-39.3945
ZINC74125465	-42.4757
ZINC74127497	-35.7016
ZINC74127497	-36.736
ZINC74137498	-35.2493
ZINC74143809	-34.8294
ZINC74159308	-41.3443
ZINC74162297	-50.5055
ZINC74162694	-38.6796
ZINC74176776	-43.3042
ZINC74190231	-41.1356
ZINC74194624	-37.4517
ZINC74201290	-34.5166
ZINC74201290	-36.5059
ZINC74208245	-50.9141
ZINC74210987	-37.1822
ZINC74212698	-40.8849
ZINC74216801	-49.0675
ZINC74228778	-39.5288
ZINC74235417	-40.6514
ZINC74238854	-43.4333
ZINC74251608	-38.7847
ZINC74251608	-40.5339
ZINC74252656	-40.3216
ZINC74257588	-41.3914
ZINC74259871	-39.4366
ZINC74264083	-44.8433
ZINC74264504	-39.9569

ZINC74269493	-40.0859
ZINC74272352	-39.121
ZINC74276797	-36.0614
ZINC74283368	-37.4077
ZINC74284535	-34.059
ZINC74289393	-43.879
ZINC74298374	-46.6282
ZINC74301624	-36.0416
ZINC74301626	-43.5125
ZINC74301927	-46.5678
ZINC74301949	-45.4349
ZINC74315041	-40.554
ZINC74315123	-39.3404
ZINC74316103	-45.7749
ZINC74323390	-39.0551
ZINC74327456	-40.1175
ZINC74328460	-48.13
ZINC74350729	-38.4351
ZINC74350729	-43.5928
ZINC74351885	-43.8692
ZINC74353813	-45.2799
ZINC74355321	-34.4218
ZINC74359484	-46.0629
ZINC74363796	-38.175
ZINC74369968	-32.4892
ZINC74372524	-35.0979
ZINC74372524	-36.6857
ZINC74372524	-40.701
ZINC74387334	-37.7126
ZINC74393594	-29.2396
ZINC74393594	-33.864
ZINC74393906	-37.1265
ZINC74394170	-38.2854
ZINC74394731	-35.7193
ZINC74397219	-37.7355
ZINC74397798	-37.2438
ZINC74400424	-37.464
ZINC74409321	-38.8444
ZINC74419091	-41.6525
ZINC74423774	-32.4717
ZINC74426089	-42.5664
ZINC74426089	-43.9314
ZINC74426121	-43.3738
ZINC74426121	-44.3765

ZINC74434031	-47.2366
ZINC74439978	-32.3023
ZINC74439978	-41.8724
ZINC74440422	-39.4212
ZINC74440835	-43.105
ZINC74440835	-49.2716
ZINC74446542	-39.3648
ZINC74451214	-41.3632
ZINC74458600	-42.8236
ZINC74458652	-44.9639
ZINC74464348	-40.45
ZINC74464348	-44.0433
ZINC74464593	-38.3303
ZINC74464720	-37.5989
ZINC74467731	-38.9003
ZINC74474218	-43.5211
ZINC74476619	-40.7226
ZINC74484922	-37.7498
ZINC74491825	-34.8887
ZINC74499672	-37.138
ZINC74504273	-44.446
ZINC74505311	-42.9324
ZINC74506298	-40.3248
ZINC74515387	-45.3293
ZINC74519030	-31.0544
ZINC74519334	-40.0621
ZINC74521531	-30.9576
ZINC74525003	-37.0751
ZINC74525444	-46.516
ZINC74532797	-41.8549
ZINC74559200	-37.1972
ZINC74562318	-43.3781
ZINC74562894	-44.5134
ZINC74569622	-47.9963
ZINC74584959	-46.6186
ZINC74605741	-45.0395
ZINC74608752	-42.8975
ZINC74615161	-37.8027
ZINC74619011	-35.4861
ZINC74619452	-38.7861
ZINC74639734	-36.2736
ZINC74640579	-28.6531
ZINC74641007	-35.3762
ZINC74641176	-46.2408

ZINC74645757	-46.7745
ZINC74646702	-35.2839
ZINC74658310	-31.5119
ZINC74658538	-37.525
ZINC74663817	-36.7223
ZINC74665414	-41.9732
ZINC74665937	-42.6409
ZINC74679581	-41.8666
ZINC74679844	-38.9047
ZINC74687488	-46.7391
ZINC74691208	-32.7414
ZINC74691208	-41.9648
ZINC74710732	-42.5541
ZINC74712443	-26.1296
ZINC74719743	-39.8242
ZINC74720380	-34.7124
ZINC74722639	-38.3678
ZINC74722639	-40.7727
ZINC74723026	-33.0209
ZINC74729391	-45.5055
ZINC74736250	-39.6329
ZINC74744950	-41.4981
ZINC74759909	-32.718
ZINC74760248	-38.9441
ZINC74762234	-38.8442
ZINC74763119	-40.551
ZINC74764038	-39.199
ZINC74770217	-33.7173
ZINC74770217	-35.9646
ZINC74773474	-44.1492
ZINC74776505	-37.5325
ZINC74776507	-35.3281
ZINC74777162	-36.9863
ZINC74779633	-41.8279
ZINC74780355	-33.9347
ZINC74780411	-45.1821
ZINC74784107	-44.135
ZINC74784107	-49.9724
ZINC74790109	-40.8153
ZINC74811977	-41.0513
ZINC74828110	-41.0922
ZINC74830894	-41.6955
ZINC74830894	-49.8459
ZINC74837565	-36.5656

ZINC74844085	-42.5913
ZINC74845691	-41.4007
ZINC74846692	-47.9511
ZINC74851188	-42.6862
ZINC74874941	-38.9214
ZINC74874941	-44.581
ZINC74874945	-36.7708
ZINC74912089	-37.5244
ZINC74912089	-43.1688
ZINC74916373	-44.5698
ZINC74926046	-36.7684
ZINC74930090	-38.5132
ZINC74934187	-37.4656
ZINC74941257	-36.8143
ZINC74941573	-32.6219
ZINC74941599	-38.7286
ZINC74943540	-32.0927
ZINC74943852	-34.5498
ZINC74973268	-38.9141
ZINC74975889	-39.4044
ZINC74980847	-36.8346
ZINC74980847	-40.2305
ZINC74992294	-39.187
ZINC74993593	-38.9023
ZINC74996816	-41.2197
ZINC75021364	-32.6459
ZINC75046613	-41.0852
ZINC75052907	-42.7572
ZINC75054788	-36.6379
ZINC75057364	-39.4915
ZINC75064318	-44.612
ZINC75064348	-40.5832
ZINC75078295	-30.4189
ZINC75092182	-45.7665
ZINC75092255	-31.8159
ZINC75092255	-34.2225
ZINC75092936	-36.1865
ZINC75092936	-38.2873
ZINC75099844	-37.9577
ZINC75099973	-39.3601
ZINC75100199	-42.7307
ZINC75106257	-29.1863
ZINC75111052	-43.4163
ZINC75116390	-35.0235

ZINC75123551	-39.4069
ZINC75126782	-43.3031
ZINC75130669	-31.9633
ZINC75157978	-49.8075
ZINC75158847	-36.3151
ZINC75158862	-41.0627
ZINC75164450	-38.3634
ZINC75164450	-43.9059
ZINC75164864	-40.0025
ZINC75165320	-39.4517
ZINC75165320	-44.5052
ZINC75166033	-42.2273
ZINC75171898	-41.0662
ZINC75178722	-49.0813
ZINC75178808	-44.0775
ZINC75181322	-36.5676
ZINC75194014	-41.6262
ZINC75221440	-35.6921
ZINC75226288	-44.5875
ZINC75236611	-38.0536
ZINC75237239	-35.5447
ZINC75237419	-40.7097
ZINC75237419	-41.9519
ZINC75239573	-29.5278
ZINC75239573	-36.5324
ZINC75240092	-41.6255
ZINC75248053	-38.4096
ZINC75249964	-32.1247
ZINC75253713	-40.7707
ZINC75267074	-36.691
ZINC75269103	-46.1908
ZINC75269998	-49.2357
ZINC75277376	-38.109
ZINC75280193	-35.9124
ZINC75286979	-41.0566
ZINC75288341	-31.8918
ZINC75292790	-30.1275
ZINC75292790	-36.6643
ZINC75292956	-27.5302
ZINC75295598	-40.1349
ZINC75299566	-31.3202
ZINC75299566	-32.5019
ZINC75299803	-36.9488
ZINC75300078	-34.5952

ZINC75300260	-41.8429
ZINC75317305	-41.2428
ZINC75337073	-43.7693
ZINC75339301	-36.8925
ZINC75340256	-39.3293
ZINC75348275	-46.4887
ZINC75353201	-40.5648
ZINC75360476	-36.1115
ZINC75369837	-43.1234
ZINC75378859	-34.245
ZINC75383730	-36.2459
ZINC75386375	-28.2349
ZINC75386375	-34.4414
ZINC75387359	-43.7382
ZINC75387874	-41.5824
ZINC75392336	-42.9527
ZINC75398817	-32.6208
ZINC75407531	-34.1951
ZINC75415902	-46.3228
ZINC75433463	-43.2502
ZINC75435842	-43.3671
ZINC75440877	-31.3678
ZINC75440881	-35.5143
ZINC75445420	-40.651
ZINC75446281	-41.8667
ZINC75447831	-38.5461
ZINC75447831	-39.8151
ZINC75449526	-35.1211
ZINC75456908	-40.9548
ZINC75456908	-43.3305
ZINC75462159	-33.4482
ZINC75463769	-38.0905
ZINC75467324	-29.0016
ZINC75478232	-43.1734
ZINC75481425	-28.7112
ZINC75486440	-43.8463
ZINC75493528	-50.8402
ZINC75503844	-31.3881
ZINC75504327	-41.1126
ZINC75508802	-34.9209
ZINC75509742	-42.5425
ZINC75533375	-38.8716
ZINC75533642	-37.1412
ZINC75534151	-39.1315

ZINC75534151	-39.4583
ZINC75535777	-37.6328
ZINC75543959	-32.9844
ZINC75543959	-39.2617
ZINC75544708	-36.8197
ZINC75546343	-40.8034
ZINC75547019	-34.7748
ZINC75547358	-49.7897
ZINC75558090	-31.6447
ZINC75559235	-40.8475
ZINC75560725	-29.0604
ZINC75561505	-39.4106
ZINC75561505	-44.698
ZINC75563734	-36.4387
ZINC75570427	-45.4901
ZINC75571976	-43.828
ZINC75578053	-42.2378
ZINC75582911	-34.3231
ZINC75587803	-44.7959
ZINC75588774	-35.8089
ZINC75589441	-36.875
ZINC75594542	-40.5996
ZINC75594542	-41.8612
ZINC75596959	-43.9901
ZINC75597671	-43.5655
ZINC75597671	-46.5144
ZINC75605706	-39.1882
ZINC75607393	-50.8245
ZINC75607627	-43.3501
ZINC75613796	-34.0024
ZINC75614274	-38.3073
ZINC75616305	-44.079
ZINC75616305	-50.7002
ZINC75624624	-35.9208
ZINC75624624	-36.916
ZINC75630366	-40.5949
ZINC75630831	-35.0391
ZINC75631493	-34.9863
ZINC75631493	-39.2607
ZINC75631891	-37.8566
ZINC75635487	-33.3507
ZINC75636997	-24.9587
ZINC75638144	-22.9149
ZINC75638793	-37.4699

ZINC75642797	-36.5186
ZINC75643156	-34.4527
ZINC75643156	-39.584
ZINC75643746	-26.7975
ZINC75645089	-42.6134
ZINC75645479	-39.2482
ZINC75645479	-40.5853
ZINC75645849	-35.799
ZINC75645849	-44.6188
ZINC75646126	-41.5628
ZINC75650946	-34.7197
ZINC75651798	-45.8529
ZINC75653582	-37.9356
ZINC75668641	-36.3523
ZINC75668884	-38.5291
ZINC75675063	-48.4183
ZINC75675158	-41.1466
ZINC75675239	-44.0354
ZINC75675242	-41.5208
ZINC75675277	-39.0245
ZINC75675277	-40.5779
ZINC75675659	-40.4878
ZINC75678778	-47.0989
ZINC75682995	-24.7985
ZINC75682995	-28.7628
ZINC75697850	-34.1674
ZINC75699111	-35.5204
ZINC75700567	-40.0474
ZINC75712627	-37.9236
ZINC75714528	-27.1237
ZINC75714528	-33.1599
ZINC75714597	-35.3198
ZINC75714597	-36.6409
ZINC75715275	-39.9481
ZINC75715441	-34.9575
ZINC75716619	-45.7095
ZINC75718604	-32.6578
ZINC75719675	-39.2519
ZINC75721673	-32.4795
ZINC75721838	-43.7428
ZINC75722312	-30.8481
ZINC75741274	-42.4129
ZINC75745265	-35.3292
ZINC75747094	-32.9584

ZINC75747828	-37.3978
ZINC75750362	-36.5806
ZINC75750362	-42.1644
ZINC75750713	-38.1697
ZINC75750713	-38.444
ZINC75750717	-27.9473
ZINC75750717	-28.7077
ZINC75751644	-32.2406
ZINC75751644	-35.413
ZINC75752432	-24.598
ZINC75755627	-39.5471
ZINC75755627	-40.9601
ZINC75763622	-44.8454
ZINC75765595	-34.564
ZINC75765744	-36.7724
ZINC75767408	-39.3348
ZINC75767408	-40.4034
ZINC75768089	-30.4013
ZINC75772802	-34.7258
ZINC75776348	-34.7303
ZINC75799425	-49.6592
ZINC75799791	-47.7508
ZINC75800277	-34.8251
ZINC75811193	-40.3103
ZINC75821186	-45.4384
ZINC75827274	-48.2073
ZINC75831215	-25.874
ZINC75833137	-42.5272
ZINC75836779	-32.328
ZINC75863745	-32.7282
ZINC75868027	-36.2424
ZINC75873565	-42.5035
ZINC75876041	-32.7231
ZINC75876041	-36.7544
ZINC75884394	-37.3336
ZINC75889688	-47.1935
ZINC75893418	-34.4829
ZINC75894785	-32.116
ZINC75894785	-37.4662
ZINC75896128	-32.7979
ZINC75897781	-38.2579
ZINC75897781	-38.9566
ZINC75922954	-30.4779
ZINC75923684	-35.7141

ZINC75923800	-28.7083
ZINC75923850	-40.4628
ZINC75923850	-45.3704
ZINC75924938	-41.6543
ZINC75935250	-38.2644
ZINC75936025	-41.1045
ZINC75961559	-46.7341
ZINC75966158	-42.4798
ZINC75984203	-37.5336
ZINC75988508	-43.8904
ZINC75989220	-34.7492
ZINC76002936	-35.0692
ZINC76002936	-37.7892
ZINC76012454	-39.5318
ZINC76021417	-39.9904
ZINC76035536	-44.5881
ZINC76036538	-35.2454
ZINC76051152	-35.5229
ZINC76051152	-38.1622
ZINC76058819	-33.398
ZINC76059009	-28.7479
ZINC76059009	-32.1721
ZINC76060083	-48.3387
ZINC76071765	-35.6775
ZINC76071765	-35.9807
ZINC76072873	-36.3944
ZINC76072873	-38.5116
ZINC76076361	-41.5134
ZINC76076505	-39.7443
ZINC76076505	-47.9006
ZINC76077304	-38.0634
ZINC76077304	-43.272
ZINC76081938	-40.6339
ZINC76093323	-40.8741
ZINC76103840	-42.7999
ZINC76107167	-41.1961
ZINC76108030	-35.9993
ZINC76118467	-36.4324
ZINC76119069	-47.0146
ZINC76119167	-33.6949
ZINC76119506	-36.9459
ZINC76120859	-34.3493
ZINC76124844	-39.4408
ZINC76128608	-37.9464

ZINC76131090	-50.4893
ZINC76137930	-42.3015
ZINC76140896	-37.1112
ZINC76141197	-39.7235
ZINC76144017	-39.5925
ZINC76144017	-39.9045
ZINC76145119	-38.5791
ZINC76148178	-47.1083
ZINC76149199	-33.234
ZINC76149381	-40.8799
ZINC76149980	-39.0198
ZINC76150302	-36.4092
ZINC76152562	-32.1
ZINC76156577	-43.7757
ZINC76156577	-45.6423
ZINC76159210	-39.3153
ZINC76162637	-43.2438
ZINC76162637	-44.96
ZINC76174866	-37.718
ZINC76183335	-40.5222
ZINC76184391	-36.8427
ZINC76184391	-39.1852
ZINC76184540	-35.9599
ZINC76184540	-46.2278
ZINC76184618	-30.0651
ZINC76200215	-39.8696
ZINC76206029	-45.834
ZINC76213518	-40.691
ZINC76215897	-46.006
ZINC76216063	-38.9506
ZINC76221836	-34.6806
ZINC76227061	-43.2201
ZINC76227439	-42.4537
ZINC76227439	-51.1811
ZINC76227767	-45.6374
ZINC76247586	-36.3903
ZINC76249721	-40.5892
ZINC76264755	-36.0177
ZINC76265029	-39.4956
ZINC76292583	-33.434
ZINC76292583	-34.3119
ZINC76292971	-35.9579
ZINC76299700	-34.8238
ZINC76299700	-35.7611

ZINC76306211	-41.0031
ZINC76309136	-37.513
ZINC76309136	-40.8363
ZINC76309227	-37.9076
ZINC76309659	-29.2023
ZINC76315784	-43.731
ZINC76331053	-33.524
ZINC76331053	-44.4364
ZINC76331364	-38.7787
ZINC76331364	-44.9447
ZINC76335123	-39.7315
ZINC76337432	-38.0141
ZINC76342683	-45.4983
ZINC76342683	-45.5338
ZINC76343407	-32.7667
ZINC76343407	-36.5473
ZINC76346277	-32.0406
ZINC76349297	-39.738
ZINC76352063	-47.3866
ZINC76352169	-38.8138
ZINC76354782	-38.6278
ZINC76357366	-45.9557
ZINC76357370	-49.014
ZINC76357806	-30.5984
ZINC76364470	-36.1871
ZINC76365923	-37.7865
ZINC76382337	-40.0605
ZINC76384043	-33.5291
ZINC76386426	-37.1464
ZINC76389503	-31.2684
ZINC76392288	-46.55
ZINC76395517	-34.9625
ZINC76395823	-34.7221
ZINC76397500	-45.1538
ZINC76400987	-37.4492
ZINC76401559	-35.1337
ZINC76403387	-35.9723
ZINC76410845	-35.9048
ZINC76411968	-35.1915
ZINC76414236	-41.8268
ZINC76416804	-38.336
ZINC76416804	-42.6019
ZINC76423159	-47.8913
ZINC76423159	-57.0451

ZINC76423175	-44.4716
ZINC76427617	-39.738
ZINC76430627	-45.5389
ZINC76453315	-28.793
ZINC76453482	-28.1398
ZINC76453482	-35.9349
ZINC76454938	-48.0449
ZINC76455239	-24.2886
ZINC76457168	-45.7074
ZINC76460042	-38.6781
ZINC76460510	-37.4798
ZINC76461486	-45.721
ZINC76461642	-41.6017
ZINC76463529	-46.575
ZINC76466763	-39.9202
ZINC76467156	-47.8184
ZINC76472339	-42.6855
ZINC76479668	-40.8828
ZINC76479712	-28.3614
ZINC76481505	-41.8686
ZINC76482459	-33.0514
ZINC76486382	-41.9105
ZINC76486573	-33.1725
ZINC76493315	-37.7858
ZINC76500143	-35.7563
ZINC76508845	-32.4456
ZINC76510167	-39.7922
ZINC76510546	-42.5284
ZINC76510774	-38.1094
ZINC76514323	-48.7372
ZINC76515265	-37.6501
ZINC76531235	-36.192
ZINC76531669	-42.5718
ZINC76546423	-36.6856
ZINC76546736	-45.1423
ZINC76546785	-29.4399
ZINC76550461	-37.4696
ZINC76558979	-48.4069
ZINC76559916	-35.0288
ZINC76561646	-39.8041
ZINC76577531	-42.9853
ZINC76587181	-40.0169
ZINC76588282	-47.4099
ZINC76592764	-33.434

ZINC76592764	-33.4531
ZINC76593139	-39.5852
ZINC76595239	-43.9554
ZINC76595239	-46.3581
ZINC76602277	-32.7816
ZINC76603756	-41.9417
ZINC76603756	-46.7027
ZINC76607927	-42.4373
ZINC76610782	-35.2388
ZINC76613088	-39.1729
ZINC76615857	-38.1262
ZINC76619882	-48.3863
ZINC76626311	-43.3878
ZINC76629468	-25.8912
ZINC76629468	-32.6333
ZINC76630663	-45.7615
ZINC76636632	-42.7094
ZINC76642022	-44.3533
ZINC76647570	-41.9409
ZINC76648788	-36.2954
ZINC76651492	-39.6186
ZINC76652886	-22.6106
ZINC76657474	-40.5396
ZINC76664334	-34.8936
ZINC76664334	-42.7535
ZINC76666781	-45.0333
ZINC76693317	-37.0107
ZINC76700068	-40.8814
ZINC76700194	-34.7519
ZINC76707825	-38.2697
ZINC76707825	-43.885
ZINC76708010	-37.0162
ZINC76708010	-41.1998
ZINC76708717	-35.972
ZINC76708717	-37.1373
ZINC76710092	-44.9708
ZINC76710360	-41.7878
ZINC76710391	-38.9105
ZINC76714628	-40.1845
ZINC76720894	-36.3624
ZINC76721925	-36.2117
ZINC76733727	-51.473
ZINC76734596	-37.8286
ZINC76734596	-38.3375

ZINC76736336	-37.1185
ZINC76736828	-35.5232
ZINC76740261	-39.7314
ZINC76745774	-42.0293
ZINC76745774	-49.5141
ZINC76752458	-34.114
ZINC76756069	-48.9758
ZINC76756541	-35.0867
ZINC76757952	-39.3798
ZINC76757967	-41.4121
ZINC76758042	-31.7394
ZINC76761609	-47.6721
ZINC76761609	-52.8202
ZINC76765037	-40.6596
ZINC76766759	-38.1821
ZINC76768827	-38.7734
ZINC76773458	-41.6207
ZINC76774692	-47.6728
ZINC76780100	-40.5401
ZINC76799268	-37.0006
ZINC76803870	-43.2569
ZINC76803870	-45.4
ZINC76804365	-48.2807
ZINC76804666	-38.2506
ZINC76810488	-32.4685
ZINC76810488	-45.4217
ZINC76815635	-36.9425
ZINC76817035	-22.1784
ZINC76817035	-40.1119
ZINC76817658	-34.6055
ZINC76819065	-33.8374
ZINC76819065	-39.9244
ZINC76820616	-40.0748
ZINC76832672	-27.1195
ZINC76843605	-45.7947
ZINC76844795	-34.283
ZINC76845415	-33.9573
ZINC76850180	-46.5583
ZINC76852611	-33.6484
ZINC76853793	-30.1848
ZINC76860310	-48.8894
ZINC76864624	-47.5905
ZINC76865154	-44.2463
ZINC76865856	-37.706

ZINC76865856	-38.673
ZINC76866405	-38.2132
ZINC76866405	-39.2617
ZINC76866407	-39.2762
ZINC76866407	-39.3568
ZINC76866501	-36.8557
ZINC76866501	-38.6457
ZINC76867342	-32.3694
ZINC76871766	-49.5129
ZINC76872325	-37.4675
ZINC76874986	-49.4158
ZINC76880161	-38.8043
ZINC76880161	-41.1422
ZINC76880258	-37.6523
ZINC76880258	-37.757
ZINC76880865	-40.6451
ZINC76896009	-44.9703
ZINC76896009	-46.9298
ZINC76910038	-37.9579
ZINC76910923	-32.3089
ZINC76911259	-38.4081
ZINC76923710	-37.516
ZINC76935088	-41.8408
ZINC76938618	-39.7989
ZINC76945773	-19.0473
ZINC76946983	-42.5198
ZINC76948193	-40.8912
ZINC76962009	-37.235
ZINC76963359	-47.1034
ZINC76987850	-37.658
ZINC76991382	-38.2487
ZINC76994266	-37.42
ZINC76994266	-38.3899
ZINC76997244	-46.9382
ZINC77002531	-35.79
ZINC77012685	-31.1424
ZINC77013038	-36.6956
ZINC77014754	-42.1727
ZINC77022583	-45.6401
ZINC77023611	-34.6425
ZINC77038315	-41.2427
ZINC77042328	-45.7542
ZINC77042328	-48.5977
ZINC77103942	-41.1302

ZINC77104669	-38.4476
ZINC77110651	-41.2752
ZINC77111023	-39.6085
ZINC77111325	-42.4023
ZINC77111690	-33.3778
ZINC77113312	-39.3074
ZINC77117163	-34.5158
ZINC77117701	-36.5413
ZINC77117755	-42.7967
ZINC77117955	-39.0709
ZINC77118044	-43.0702
ZINC77121399	-32.9959
ZINC77121656	-41.5761
ZINC77131474	-46.9665
ZINC77136364	-46.7908
ZINC77136364	-47.8653
ZINC77141611	-45.6878
ZINC77142814	-41.4223
ZINC77148050	-45.3221
ZINC77149977	-35.6461
ZINC77156725	-41.6389
ZINC77159649	-39.7083
ZINC77159997	-42.6514
ZINC77161867	-42.2397
ZINC77164803	-32.4566
ZINC77165981	-47.1857
ZINC77167594	-35.2259
ZINC77175222	-46.929
ZINC77176665	-38.4996
ZINC77177675	-34.7682
ZINC77182015	-43.2886
ZINC77192095	-36.5544
ZINC77192471	-45.059
ZINC77192655	-32.0092
ZINC77192655	-42.5089
ZINC77192660	-37.6595
ZINC77197729	-49.5051
ZINC77204124	-49.4552
ZINC77208166	-40.714
ZINC77208689	-39.2804
ZINC77209499	-37.7446
ZINC77209499	-38.5748
ZINC77210568	-42.0127
ZINC77215978	-42.9649

ZINC77218983	-43.9065
ZINC77218983	-48.3784
ZINC77220676	-45.617
ZINC77223974	-43.5394
ZINC77225471	-36.05
ZINC77225471	-37.3247
ZINC77235900	-43.035
ZINC77237068	-38.2094
ZINC77237068	-42.0031
ZINC77237068	-42.7153
ZINC77237572	-38.002
ZINC77238175	-38.3709
ZINC77238689	-41.4401
ZINC77241540	-36.8077
ZINC77242165	-34.9032
ZINC77247700	-38.4846
ZINC77247706	-36.1477
ZINC77249386	-36.5512
ZINC77265636	-42.4086
ZINC77265731	-44.4
ZINC77266690	-37.025
ZINC77266690	-47.582
ZINC77267402	-36.2542
ZINC77267613	-48.3649
ZINC77268229	-43.1159
ZINC77269172	-40.7289
ZINC77269172	-43.1243
ZINC77272072	-43.5569
ZINC77273642	-43.3537
ZINC77274956	-38.3143
ZINC77275646	-40.9645
ZINC77277682	-37.3158
ZINC77287483	-44.4005
ZINC77288120	-36.4488
ZINC77288120	-38.6802
ZINC77292238	-46.8011
ZINC77301821	-36.4656
ZINC77306834	-36.0526
ZINC77313686	-40.2949
ZINC77316385	-34.6684
ZINC77316385	-36.383
ZINC77316707	-35.5054
ZINC77316944	-33.5096
ZINC77316944	-35.0979

ZINC77322844	-42.5162
ZINC77322844	-43.5528
ZINC77323671	-39.5372
ZINC77329157	-39.2115
ZINC77329157	-42.6629
ZINC77330881	-36.4183
ZINC77330881	-38.1934
ZINC77334546	-49.1702
ZINC77340994	-28.4839
ZINC77342263	-42.0201
ZINC77342640	-29.3872
ZINC77342640	-32.066
ZINC77342801	-34.9119
ZINC77342801	-43.134
ZINC77343755	-37.7746
ZINC77343755	-42.1001
ZINC77344371	-38.9435
ZINC77347372	-43.5787
ZINC77352461	-40.1858
ZINC77352583	-37.0149
ZINC77352690	-33.2579
ZINC77353962	-38.5158
ZINC77359994	-25.9145
ZINC77359994	-33.5728
ZINC77361410	-36.0846
ZINC77361410	-39.8489
ZINC77369987	-30.9593
ZINC77369987	-39.4521
ZINC77370121	-28.7536
ZINC77370121	-38.2093
ZINC77375342	-31.7301
ZINC77375342	-46.5774
ZINC77402515	-40.1036
ZINC77407018	-35.305
ZINC77407561	-46.9668
ZINC77407780	-35.6304
ZINC77412303	-39.632
ZINC77415356	-44.0594
ZINC77422747	-50.6124
ZINC77422747	-50.7982
ZINC77425383	-39.5518
ZINC77425389	-38.3915
ZINC77430160	-42.3891
ZINC77432435	-28.1584

ZINC77444086	-36.4823
ZINC77450453	-37.421
ZINC77454869	-37.9276
ZINC77454869	-40.8187
ZINC77455321	-41.0917
ZINC77455723	-41.3235
ZINC77457238	-46.691
ZINC77458012	-28.7297
ZINC77459228	-45.0514
ZINC77459452	-46.0364
ZINC77461920	-42.0529
ZINC77466645	-33.3042
ZINC77466645	-35.9776
ZINC77467493	-41.8283
ZINC77468144	-39.741
ZINC77468144	-41.113
ZINC77469868	-24.6992
ZINC77469911	-31.9052
ZINC77469911	-35.8944
ZINC77470303	-39.697
ZINC77470303	-40.0376
ZINC77472438	-32.6267
ZINC77477412	-34.2953
ZINC77478304	-52.2872
ZINC77480787	-40.5576
ZINC77485518	-37.0621
ZINC77485518	-39.4832
ZINC77485817	-33.1934
ZINC77486801	-35.5925
ZINC77487229	-30.6469
ZINC77491353	-38.7215
ZINC77491743	-36.9622
ZINC77492262	-46.6861
ZINC77496646	-40.5788
ZINC77506414	-43.6801
ZINC77506715	-33.8665
ZINC77508086	-41.7596
ZINC77508761	-42.5658
ZINC77508891	-33.7118
ZINC77514610	-43.1089
ZINC77525838	-38.0284
ZINC77525838	-39.1112
ZINC77527657	-40.8783
ZINC77530097	-43.1082

ZINC77531221	-44.1497
ZINC77536826	-35.9569
ZINC77537307	-40.6167
ZINC77537496	-32.1497
ZINC77537756	-43.0907
ZINC77538419	-33.9292
ZINC77538434	-45.3214
ZINC77542188	-41.1354
ZINC77542854	-41.6766
ZINC77543333	-40.1958
ZINC77545458	-36.1277
ZINC77551778	-35.049
ZINC77553473	-34.5568
ZINC77557041	-28.7102
ZINC77557041	-34.9999
ZINC77569470	-40.7766
ZINC77571623	-53.8699
ZINC77573142	-39.985
ZINC77573142	-45.3274
ZINC77574915	-33.6965
ZINC77586868	-35.4403
ZINC77588942	-34.9017
ZINC77588942	-40.3076
ZINC77592347	-37.6961
ZINC77603001	-43.6369
ZINC77611073	-40.0404
ZINC77611146	-42.7389
ZINC77612327	-36.69
ZINC77614749	-39.0616
ZINC77616828	-43.8071
ZINC77617364	-47.6991
ZINC77620534	-40.8365
ZINC77630062	-42.8317
ZINC77631031	-45.5469
ZINC77634162	-40.6631
ZINC77645717	-40.6657
ZINC77646873	-32.5757
ZINC77652072	-34.4473
ZINC77653598	-38.4006
ZINC77654248	-42.7744
ZINC77654559	-38.5792
ZINC77657933	-48.9794
ZINC77661289	-45.2811
ZINC77665172	-45.3277

ZINC77665423	-44.5302
ZINC77670262	-38.39
ZINC77684692	-37.6098
ZINC77687562	-42.9372
ZINC77688957	-43.4774
ZINC77689437	-40.4173
ZINC77689776	-37.3465
ZINC77690150	-43.6696
ZINC77690169	-47.5297
ZINC77695303	-40.7477
ZINC77696146	-36.2619
ZINC77696195	-38.4766
ZINC77697903	-44.0519
ZINC77701723	-37.4669
ZINC77718924	-47.795
ZINC77718951	-41.0799
ZINC77719031	-36.7562
ZINC77721175	-37.5619
ZINC77721175	-45.4945
ZINC77727234	-47.2793
ZINC77732535	-40.9681
ZINC77732535	-46.1022
ZINC77732957	-39.8001
ZINC77739315	-37.2916
ZINC77741155	-36.7197
ZINC77741966	-39.7317
ZINC77745197	-45.3228
ZINC77752924	-40.4121
ZINC77766026	-46.8125
ZINC77767044	-47.8905
ZINC77771325	-46.0265
ZINC77781336	-45.5518
ZINC77784887	-31.7901
ZINC77790363	-41.9434
ZINC77796458	-37.1239
ZINC77802320	-45.5059
ZINC77811793	-38.4708
ZINC77811934	-38.3351
ZINC77816697	-35.255
ZINC77817501	-32.534
ZINC77865607	-41.5846
ZINC77868836	-47.9448
ZINC77872058	-45.1535
ZINC77873931	-38.8941

ZINC77880271	-35.1726
ZINC77886986	-36.1421
ZINC77887736	-39.5546
ZINC77891866	-45.8748
ZINC77892977	-30.947
ZINC77894636	-36.9387
ZINC77897414	-41.8868
ZINC77897506	-37.3241
ZINC77901410	-36.3846
ZINC77903728	-31.466
ZINC77909632	-36.5337
ZINC77917319	-48.5082
ZINC77917338	-47.5892
ZINC77927417	-37.4245
ZINC77928955	-36.3769
ZINC77928955	-41.8894
ZINC77936235	-42.4687
ZINC77941128	-40.1385
ZINC77941128	-42.9986
ZINC77942274	-42.3888
ZINC77947720	-39.4299
ZINC77947745	-51.5345
ZINC77948106	-52.3911
ZINC77948235	-44.7839
ZINC77955905	-28.3338
ZINC77956133	-39.1326
ZINC77959568	-45.026
ZINC77960086	-41.9322
ZINC77962539	-40.2815
ZINC77962539	-49.5113
ZINC77962683	-41.9484
ZINC77968772	-47.9278
ZINC77973835	-35.0601
ZINC77978155	-48.4729
ZINC77983151	-34.8498
ZINC77983151	-36.8933
ZINC77984561	-42.5073
ZINC77984561	-44.2625
ZINC77984858	-37.9104
ZINC77984858	-40.6403
ZINC77985821	-34.6126
ZINC77986493	-33.7181
ZINC77991234	-41.2297
ZINC77995049	-36.2747

ZINC77995575	-38.839
ZINC77999845	-32.2922
ZINC78000806	-43.0706
ZINC78003183	-35.9048
ZINC78006586	-39.8931
ZINC78021715	-35.3805
ZINC78032684	-47.4629
ZINC78038080	-36.9389
ZINC78040916	-45.784
ZINC78040940	-37.7517
ZINC78046616	-34.9143
ZINC78046744	-27.0587
ZINC78046744	-29.9381
ZINC78048755	-47.7885
ZINC78052301	-37.508
ZINC78052706	-38.9279
ZINC78055172	-40.5786
ZINC78064128	-32.9354
ZINC78069450	-34.5197
ZINC78071634	-39.9997
ZINC78077373	-35.1472
ZINC78079354	-39.5828
ZINC78082542	-46.5342
ZINC78084817	-33.6731
ZINC78086402	-42.4746
ZINC78091561	-40.2295
ZINC78093664	-45.4101
ZINC78093848	-32.8854
ZINC78102031	-33.6982
ZINC78109697	-38.1561
ZINC78148897	-43.1563
ZINC78149024	-38.4045
ZINC78149388	-43.6577
ZINC78150606	-36.6411
ZINC78150606	-40.1044
ZINC78155438	-35.1285
ZINC78155478	-39.1069
ZINC78169219	-35.21
ZINC78177497	-34.581
ZINC78177561	-32.4548
ZINC78181722	-32.9136
ZINC78181920	-32.9324
ZINC78187734	-44.9582
ZINC78188118	-44.0252

ZINC78190618	-37.0936
ZINC78204641	-39.5715
ZINC78206947	-42.4897
ZINC78210483	-44.2763
ZINC78224662	-37.7279
ZINC78229451	-40.9379
ZINC78231716	-37.7377
ZINC78244454	-32.9695
ZINC78248806	-41.1773
ZINC78248852	-34.1475
ZINC78248858	-43.2129
ZINC78253416	-34.9686
ZINC78254593	-42.568
ZINC78255737	-30.6633
ZINC78258627	-40.22
ZINC78259199	-36.4025
ZINC78261178	-37.2174
ZINC78262672	-36.5942
ZINC78263945	-40.0374
ZINC78264225	-34.4644
ZINC78266460	-39.8199
ZINC78266684	-44.3549
ZINC78267063	-35.3577
ZINC78267063	-37.09
ZINC78267303	-45.8101
ZINC78267303	-49.3554
ZINC78288263	-33.713
ZINC78288263	-35.1367
ZINC78289685	-29.7608
ZINC78289699	-40.9831
ZINC78289910	-33.0833
ZINC78290488	-34.9624
ZINC78290979	-43.7517
ZINC78293983	-42.4924
ZINC78294420	-37.8463
ZINC78294711	-38.7787
ZINC78294716	-30.8344
ZINC78294716	-34.9328
ZINC78295233	-26.8668
ZINC78296216	-38.8303
ZINC78298132	-38.4297
ZINC78299173	-39.0684
ZINC78299866	-32.4513
ZINC78300528	-40.0028

ZINC78300528	-41.7429
ZINC78300912	-41.5087
ZINC78301293	-31.5143
ZINC78302440	-29.9809
ZINC78302440	-42.5524
ZINC78307111	-38.8437
ZINC78308397	-40.8474
ZINC78308397	-53.7203
ZINC78308436	-44.3818
ZINC78308986	-47.3825
ZINC78315248	-32.8565
ZINC78315248	-36.2689
ZINC78319523	-51.7988
ZINC78322640	-42.7694
ZINC78325733	-35.2547
ZINC78328063	-43.0578
ZINC78330164	-39.5575
ZINC78330561	-46.2284
ZINC78331278	-38.1182
ZINC78336726	-39.3123
ZINC78337500	-39.8217
ZINC78337980	-38.9647
ZINC78337980	-44.5701
ZINC78340723	-48.1972
ZINC78340978	-38.5161
ZINC78341887	-36.5155
ZINC78341887	-41.1264
ZINC78342291	-36.3196
ZINC78345615	-32.1747
ZINC78348094	-52.1286
ZINC78348432	-38.6514
ZINC78348879	-45.9913
ZINC78351289	-40.0808
ZINC78354038	-34.3172
ZINC78354589	-41.4441
ZINC78355776	-44.4593
ZINC78358643	-39.1228
ZINC78360562	-36.8482
ZINC78362200	-42.2697
ZINC78362248	-42.0144
ZINC78362828	-39.1396
ZINC78362828	-45.7031
ZINC78363097	-32.737
ZINC78365644	-36.2927

ZINC78368509	-43.6459
ZINC78372496	-36.6436
ZINC78376011	-37.8868
ZINC78376175	-34.0417
ZINC78376549	-39.8012
ZINC78378277	-39.9322
ZINC78381935	-34.3346
ZINC78381935	-38.175
ZINC78382480	-33.4493
ZINC78385045	-37.8953
ZINC78385776	-38.3031
ZINC78385776	-39.7916
ZINC78395987	-45.1015
ZINC78396073	-35.1687
ZINC78399713	-34.9171
ZINC78400379	-41.73
ZINC78403552	-42.6139
ZINC78404272	-37.8835
ZINC78404272	-43.1155
ZINC78404585	-31.2039
ZINC78404585	-34.419
ZINC78404874	-40.5388
ZINC78406697	-44.2826
ZINC78411021	-36.8693
ZINC78415676	-46.5931
ZINC78415694	-37.58
ZINC78416148	-40.6724
ZINC78418532	-39.743
ZINC78418657	-45.2186
ZINC78418657	-47.2109
ZINC78419924	-35.0763
ZINC78424218	-42.7171
ZINC78424218	-44.8987
ZINC78424657	-39.997
ZINC78428535	-40.3616
ZINC78431209	-40.7979
ZINC78431929	-39.054
ZINC78435883	-36.6545
ZINC78436475	-36.6554
ZINC78436475	-38.3867
ZINC78437054	-39.906
ZINC78438282	-37.8596
ZINC78442229	-39.5168
ZINC78449052	-39.7608

ZINC78449052	-46.2735
ZINC78449327	-33.9399
ZINC78449327	-34.6022
ZINC78451832	-33.3343
ZINC78453405	-37.9238
ZINC78453848	-52.0292
ZINC78454980	-46.2577
ZINC78460840	-40.5837
ZINC78461109	-38.2676
ZINC78462703	-46.4448
ZINC78462768	-34.543
ZINC78463193	-42.1575
ZINC78464337	-41.6258
ZINC78468404	-45.2558
ZINC78469441	-50.3592
ZINC78474968	-40.4515
ZINC78477721	-41.4298
ZINC78479814	-39.5938
ZINC78479924	-41.8818
ZINC78481853	-40.6265
ZINC78488732	-37.8929
ZINC78488907	-41.8228
ZINC78489588	-36.3962
ZINC78493011	-41.508
ZINC78511383	-37.498
ZINC78512443	-37.4154
ZINC78513439	-38.0436
ZINC78514547	-40.2926
ZINC78514945	-38.3538
ZINC78514945	-40.5899
ZINC78515447	-35.346
ZINC78515447	-43.2553
ZINC78527736	-37.9268
ZINC78527736	-42.0059
ZINC78527978	-40.58
ZINC78527978	-45.4267
ZINC78530163	-34.7151
ZINC78531425	-38.3385
ZINC78535128	-41.6201
ZINC78535406	-37.9304
ZINC78535452	-32.9029
ZINC78535585	-47.9438
ZINC78535613	-36.5656
ZINC78540122	-39.1941

ZINC78541197	-39.1812
ZINC78542664	-43.4965
ZINC78543615	-42.5854
ZINC78543667	-38.4657
ZINC78544448	-42.6792
ZINC78545137	-46.5184
ZINC78545579	-37.1899
ZINC78546281	-34.808
ZINC78556546	-30.8184
ZINC78562376	-40.6176
ZINC78563252	-40.7758
ZINC78567687	-36.1505
ZINC78568718	-42.0367
ZINC78570042	-43.1945
ZINC78572551	-33.15
ZINC78576632	-39.8099
ZINC78577993	-37.828
ZINC78583037	-39.9844
ZINC78584352	-42.1555
ZINC78586203	-28.847
ZINC78596537	-30.6966
ZINC78598966	-32.0397
ZINC78602833	-29.155
ZINC78608404	-38.429
ZINC78611087	-34.2808
ZINC78611087	-41.4843
ZINC78614447	-43.9386
ZINC78615866	-33.8344
ZINC78618154	-39.1955
ZINC78619325	-38.7811
ZINC78619325	-44.1892
ZINC78619780	-37.3884
ZINC78619780	-37.9615
ZINC78620283	-37.71
ZINC78620313	-36.6815
ZINC78624651	-36.0457
ZINC78624657	-34.1489
ZINC78627442	-37.7449
ZINC78632147	-37.1289
ZINC78638322	-38.3119
ZINC78645707	-38.5491
ZINC78645836	-44.3224
ZINC78647867	-31.9272
ZINC78647973	-43.6448

ZINC78649133	-35.042
ZINC78649868	-41.7329
ZINC78649868	-45.8042
ZINC78650895	-38.4418
ZINC78656643	-38.5369
ZINC78659055	-42.2108
ZINC78660332	-36.2869
ZINC78665574	-35.1805
ZINC78665971	-36.3306
ZINC78665971	-44.1847
ZINC78666797	-38.1139
ZINC78666919	-36.686
ZINC78667692	-32.5495
ZINC78668089	-35.491
ZINC78669261	-42.0282
ZINC78671681	-38.4226
ZINC78672539	-33.3459
ZINC78672868	-44.2416
ZINC78673535	-38.8789
ZINC78673726	-35.5014
ZINC78680579	-33.2364
ZINC78692524	-41.583
ZINC78693769	-31.6815
ZINC78694328	-44.1227
ZINC78694870	-43.7212
ZINC78694891	-36.474
ZINC78697496	-40.1961
ZINC78701247	-44.5945
ZINC78701978	-39.5658
ZINC78702718	-47.4907
ZINC78702718	-49.0741
ZINC78705652	-26.8868
ZINC78709333	-30.103
ZINC78709414	-42.029
ZINC78709514	-39.5445
ZINC78709514	-39.8798
ZINC78710523	-42.0613
ZINC78712440	-39.4017
ZINC78712626	-44.3501
ZINC78713191	-43.5876
ZINC78715863	-40.7179
ZINC78716153	-40.15
ZINC78717114	-41.3872
ZINC78720097	-40.9262

ZINC78721290	-47.2943
ZINC78723245	-42.6931
ZINC78725941	-36.764
ZINC78729386	-38.5075
ZINC78730551	-29.8425
ZINC78730551	-34.5742
ZINC78732273	-31.1835
ZINC78732531	-34.8498
ZINC78733071	-38.6669
ZINC78736656	-38.2006
ZINC78736656	-44.1944
ZINC78741572	-41.8368
