# Supplementary material for: Cheminformatics Models for Inhibitors of Schistosoma mansoni Thioredoxin Glutathione Reductase
Source: ScientificWorldJournal. 2014 Nov 25;2014:957107. doi: 10.1155/2014/957107 (PMC4275605; doi:10.1155/2014/957107)
Supplement: Supplementary file 1 — Supplementary Table 1 contains the detailed information of all descriptors included in the three major descriptors (Pharmacophore fingerprints, Weighted burden and Properties) used in our study. Supplementary Table 2 shows 177 scaffolds which have frequency greater than 0.1 percent in actives. These scaffolds were selected for further analysis. Additional File contains the docking scores of molecules from AID485634 and ZINC which were predicted active by Random Forest model and contains one of the enriched scaffolds. It also shows the docking scores of ZINC molecules predicted inactive by the model and the ones which were predicted active but do not contain any enriched substructure. [file 957107.f1.zip › Supplementary Table 1.pdf]

Supplementary Table 1. Descriptors generated by PowerMV used for model generation

Pharmacophore Fingerprint	Weighted Burden Number	Property
NEG_01_NEG, NEG_02_NEG, NEG_03_NEG, NEG_04_NEG, NEG_05_NEG, NEG_06_NEG, NEG_07_NEG, NEG_03_POS, NEG_04_POS, NEG_05_POS, NEG_06_POS, NEG_07_POS, NEG_01_HBD, NEG_02_HBD, NEG_03_HBD, NEG_04_HBD, NEG_05_HBD, NEG_06_HBD, NEG_07_HBD, NEG_03_HBA, NEG_04_HBA, NEG_05_HBA, NEG_06_HBA, NEG_07_HBA, NEG_02_ARC, NEG_03_ARC, NEG_04_ARC, NEG_05_ARC, NEG_06_ARC, NEG_07_ARC, NEG_02_HYP, NEG_03_HYP, NEG_04_HYP, NEG_05_HYP, NEG_06_HYP, NEG_07_HYP, POS_03_POS, POS_04_POS, POS_05_POS, POS_06_POS, POS_07_POS, POS_02_HBD, POS_03_HBD, POS_04_HBD, POS_05_HBD, POS_06_HBD, POS_07_HBD, POS_03_HBA, POS_04_HBA, POS_05_HBA, POS_06_HBA, POS_07_HBA, POS_02_ARC, POS_03_ARC, POS_04_ARC, POS_05_ARC, POS_06_ARC, POS_07_ARC, POS_02_HYP, POS_03_HYP, POS_04_HYP, POS_05_HYP, POS_06_HYP, POS_07_HYP, HBD_03_HBD, HBD_04_HBD, HBD_05_HBD, HBD_06_HBD, HBD_07_HBD, HBD_03_HBA, HBD_04_HBA, HBD_05_HBA, HBD_06_HBA, HBD_07_HBA, HBD_02_ARC, HBD_03_ARC, HBD_04_ARC, HBD_05_ARC, HBD_06_ARC, HBD_07_ARC,	WBN_GC_L_0.25<br>WBN_GC_H_0.25<br>WBN_GC_L_0.50<br>WBN_GC_H_0.50<br>WBN_GC_L_0.75<br>WBN_GC_H_0.75<br>WBN_GC_L_1.00<br>WBN_GC_H_1.00<br>WBN_EN_L_0.25<br>WBN_EN_H_0.25<br>WBN_EN_L_0. 50<br>WBN_EN_H_0. 50<br>WBN_EN_L_0.75<br>WBN_EN_H_0.75<br>WBN_EN_L_1.00<br>WBN_EN_H_1.00<br>WBN_LP_L_0.25<br>WBN_ LP _H_0.25<br>WBN_ LP _L_0. 50	XLogP<br>PSA,<br>NumRot<br>NumHBA<br>NumHBD<br>MW<br>BBB<br>BadGroup

HBD_02_HYP, HBD_03_HYP, HBD_04_HYP, HBD_05_HYP, HBD_06_HYP, HBD_07_HYP, HBA_03_HBA, HBA_04_HBA, HBA_05_HBA, HBA_06_HBA, HBA_07_HBA, HBA_03_ARC, HBA_04_ARC, HBA_05_ARC, HBA_06_ARC, HBA_07_ARC, HBA_02_HYP, HBA_03_HYP, HBA_04_HYP, HBA_05_HYP, HBA_06_HYP, HBA_07_HYP, ARC_01_ARC, ARC_02_ARC, ARC_03_ARC, ARC_04_ARC, ARC_05_ARC, ARC_06_ARC, ARC_07_ARC, ARC_02_HYP, ARC_03_HYP, ARC_04_HYP, ARC_05_HYP, ARC_06_HYP, ARC_07_HYP, HYP_01_HYP, HYP_02_HYP, HYP_03_HYP, HYP_04_HYP, HYP_05_HYP, HYP_06_HYP, HYP_07_HYP	WBN_ LP _H_0. 50<br>WBN_ LP _L_0.75<br>WBN_ LP _H_0.75<br>WBN_ LP _L_1.00<br>WBN_ LP _H_1.00	
