# Supplementary material for: Cheminformatics Models for Inhibitors of Schistosoma mansoni Thioredoxin Glutathione Reductase
Source: ScientificWorldJournal. 2014 Nov 25;2014:957107. doi: 10.1155/2014/957107 (PMC4275605; doi:10.1155/2014/957107)
Supplement: Supplementary file 1 — Supplementary Table 1 contains the detailed information of all descriptors included in the three major descriptors (Pharmacophore fingerprints, Weighted burden and Properties) used in our study. Supplementary Table 2 shows 177 scaffolds which have frequency greater than 0.1 percent in actives. These scaffolds were selected for further analysis. Additional File contains the docking scores of molecules from AID485634 and ZINC which were predicted active by Random Forest model and contains one of the enriched scaffolds. It also shows the docking scores of ZINC molecules predicted inactive by the model and the ones which were predicted active but do not contain any enriched substructure. [file 957107.f1.zip › Supplementary Table 2.pdf]

Supplementary Table 2. Substructures having frequency greater than 0.1 percent in actives

Smiles	Matches in Actives (10735)	Matches in Inactives (331528)	Actives (without motif)	Inactives (without motif)	Chi-square	p-value	Frequency in Actives	Frequency in Inactives	Enrichment Factor
CCNc1ccccc1	2160	84190	8575	247338	153.290	0.00	0.201	0.254	0.79
CNCc1ccccc1	1894	69382	8841	262146	68.044	0.00	0.176	0.209	0.84
O=CNc1ccccc1	1883	87065	8852	244463	411.164	0.00	0.175	0.263	0.67
O=S(=O)c1ccccc1	1281	48198	9454	283330	57.069	0.00	0.119	0.145	0.82
CN(C)c1ccccc1	1268	37992	9467	293536	1.270	0.26	0.118	0.115	1.03
CCCc1ccccc1	1228	29239	9507	302289	88.004	0.00	0.114	0.088	1.30
COc1ccc(C)cc1	955	37682	9780	293846	63.349	0.00	0.089	0.114	0.78
Oc1ccccc1O	861	30358	9874	301170	16.202	0.00	0.080	0.092	0.88
NCCc1ccccc1	810	28339	9925	303189	13.415	0.00	0.075	0.085	0.88
CCOc1ccccc1	783	38282	9952	293246	186.041	0.00	0.073	0.115	0.63
COc1cccc(C)c1	727	27612	10008	303916	33.171	0.00	0.068	0.083	0.81
CC(=O)c1ccccc1	697	10137	10038	321391	400.301	0.00	0.065	0.031	2.12
NCNc1ccccc1	689	15220	10046	316308	78.346	0.00	0.064	0.046	1.40
O=CNCc1ccco1	639	3271	10096	328257	2270.504	0.00	0.060	0.010	6.03
CCc1ccc(O)cc1	621	15017	10114	316511	37.572	0.00	0.058	0.045	1.28
C\C=C\c1ccccc1	585	8786	10150	322742	305.982	0.00	0.054	0.027	2.06
CCN(CC)CCNC	550	14244	10185	317284	17.195	0.00	0.051	0.043	1.19
ON(=O)c1ccccc1	533	14382	10202	317146	9.807	0.00	0.050	0.043	1.14
OC(=O)c1ccccc1	506	15062	10229	316466	0.695	0.40	0.047	0.045	1.04
c:c:c1cccnc1:c	487	11729	10248	319799	30.133	0.00	0.045	0.035	1.28
O=C\C=C\c1ccco1	460	342	10275	331186	7778.789	0.00	0.043	0.001	41.54

S=CNc1ccccc1	434	5614	10301	325914	330.671	0.00	0.040	0.017	2.39
COc1ccc(N)cc1	428	17033	10307	314495	28.443	0.00	0.040	0.051	0.78
COCc1ccccc1	407	12850	10328	318678	0.200	0.65	0.038	0.039	0.98
CCc1ccccc1C	371	6556	10364	324972	114.626	0.00	0.035	0.020	1.75
c:c:c1ccccc1:c	318	6408	10417	325120	57.195	0.00	0.030	0.019	1.53
CCSc1ccccc1	308	5450	10427	326078	94.372	0.00	0.029	0.016	1.75
CCCCCCCNC	293	10635	10442	320893	7.702	0.01	0.027	0.032	0.85
Nc1cccc(O)c1	256	10850	10479	320678	26.117	0.00	0.024	0.033	0.73
c1cc2ccccc2n1	255	10028	10480	321500	15.047	0.00	0.024	0.030	0.79
CCNC(=O)c1ccco1	252	4052	10483	327476	106.032	0.00	0.023	0.012	1.92
CNCc1ccc(C)o1	225	1136	10510	330392	807.055	0.00	0.021	0.003	6.12
c1coc(n1)-c1ccccc1	218	1868	10517	329660	369.568	0.00	0.020	0.006	3.60
C=C1C(=O)NCNC1=O	213	913	10522	330615	925.941	0.00	0.020	0.003	7.20
CCCCCCCCC	195	4645	10540	326883	12.870	0.00	0.018	0.014	1.30
c1nc2ccccc2n1	193	7615	10542	323913	11.618	0.00	0.018	0.023	0.78
Cc1ccc(o1)-c1ccccc1	185	939	10550	330589	658.825	0.00	0.017	0.003	6.08
c1cnn(c1)-c1ccccc1	147	6855	10588	324673	25.306	0.00	0.014	0.021	0.66
CCS(=O)Cc1ncoc1C	146	218	10589	331310	1639.609	0.00	0.014	0.001	20.68
C\C=C\Nc1ccccc1	144	2317	10591	329211	60.134	0.00	0.013	0.007	1.92
CCCN1CCCCC1	139	4218	10596	327310	0.042	0.84	0.013	0.013	1.02
CSc1nccc(C)n1	138	1853	10597	329675	94.921	0.00	0.013	0.006	2.30
c1ccc2nccnc2c1	138	2903	10597	328625	19.837	0.00	0.013	0.009	1.47
c:c:c1occcc1:c	129	6067	10606	325461	23.095	0.00	0.012	0.018	0.66
CCSc1nncn1C	122	3475	10613	328053	0.780	0.38	0.011	0.010	1.08
Cc1cccn1-c1ccccc1	121	973	10614	330555	226.818	0.00	0.011	0.003	3.84
COc1ccc(O)cc1	120	5544	10615	325984	19.639	0.00	0.011	0.017	0.67
CCCN1CCOCC1	115	2510	10620	329018	13.485	0.00	0.011	0.008	1.41
CC(O)c1ccccc1	110	2536	10625	328992	9.145	0.00	0.010	0.008	1.34
O=S(=O)Cc1ccccc1	107	914	10628	330614	181.769	0.00	0.010	0.003	3.62

CNCc1ccncc1	104	2356	10631	329172	9.711	0.00	0.010	0.007	1.36
c1cc2cncnc2s1	103	3747	10632	327781	2.726	0.10	0.010	0.011	0.85
CCc1c(C)csc1C	101	1971	10634	329557	20.727	0.00	0.009	0.006	1.58
CCCCC(=O)OCC	101	2624	10634	328904	2.937	0.09	0.009	0.008	1.19
Cc1cc(C=O)c(N)s1	100	2151	10635	329377	12.721	0.00	0.009	0.006	1.44
Cc1ccsc1NC=O	97	2788	10638	328740	0.488	0.48	0.009	0.008	1.07
COC(=O)C(C)=C(C)N	92	1585	10643	329943	30.621	0.00	0.009	0.005	1.79
CC(=O)Nc1nccs1	88	3415	10647	328113	4.541	0.03	0.008	0.010	0.80
Cc1cc(O)ccc1O	86	1448	10649	330080	30.938	0.00	0.008	0.004	1.83
CNCc1ccccn1	86	1874	10649	329654	10.159	0.00	0.008	0.006	1.42
c:c:c1ncncc1:c	84	7956	10651	323572	118.570	0.00	0.008	0.024	0.33
CCCC(=O)NC(C)C	83	3928	10652	327600	15.214	0.00	0.008	0.012	0.65
NC(=O)c1cccnc1	82	4664	10653	326864	31.436	0.00	0.008	0.014	0.54
Cc1ccc(o1)N(O)=O	81	152	10654	331376	767.677	0.00	0.008	0.000	16.46
CNNc1ccccc1	81	784	10654	330744	110.705	0.00	0.008	0.002	3.19
c1ccc(cc1)-c1ccccc1	81	2557	10654	328971	0.038	0.85	0.008	0.008	0.98
CCCCN(C)S(=O)=O	81	9898	10654	321630	182.850	0.00	0.008	0.030	0.25
N\N=C\c1ccccc1	80	6383	10655	325145	78.164	0.00	0.007	0.019	0.39
Cc1cccc(=O)n1C	77	476	10658	331052	212.164	0.00	0.007	0.001	5.00
Cc1ccc(N)c(C)c1	77	2700	10658	328828	1.219	0.27	0.007	0.008	0.88
O=[S]NCc1ccco1	76	290	10659	331238	374.780	0.00	0.007	0.001	8.09
NNC(=O)c1ccccc1	75	4957	10660	326571	45.545	0.00	0.007	0.015	0.47
CN\N=C\c1ccco1	74	624	10661	330904	128.300	0.00	0.007	0.002	3.66
N=C\C=C\c1ccco1	71	47	10664	331481	1263.811	0.00	0.007	0.000	46.65
c1coc(c1)-c1ncnn1	71	520	10664	331008	153.559	0.00	0.007	0.002	4.22
COC1CCC=C(C)O1	70	433	10665	331095	192.683	0.00	0.007	0.001	4.99
CCCC\C=C\CC=C	68	673	10667	330855	89.182	0.00	0.006	0.002	3.12
C=NNc1ccccc1	68	1661	10667	329867	3.628	0.06	0.006	0.005	1.26
SCNc1ccccc1	67	1600	10668	329928	4.296	0.04	0.006	0.005	1.29

O=C1NCC2CC=CCC12	64	903	10671	330625	38.698	0.00	0.006	0.003	2.19
Cc1cc(:c)c(:c:c)o1	63	2701	10672	328827	6.739	0.01	0.006	0.008	0.72
CC(=O)N1CCCCC1	63	4783	10672	326745	54.566	0.00	0.006	0.014	0.41
NC(=S)NCc1ccco1	62	468	10673	331060	128.074	0.00	0.006	0.001	4.09
c1coc(c1)-c1cccnn1	61	20	10674	331508	1389.073	0.00	0.006	0.000	94.19
CCN(CC)C(=O)CS	61	2922	10674	328606	11.802	0.00	0.006	0.009	0.64
C1CCc2sccc2C1	58	1727	10677	329801	0.075	0.78	0.005	0.005	1.04
CS(=O)(=O)c1cccs1	57	72	10678	331456	715.761	0.00	0.005	0.000	24.45
CCCCCOC(C)=O	57	1009	10678	330519	17.200	0.00	0.005	0.003	1.74
Cn1ccc(=O)n(C)c1	55	4313	10680	327215	51.326	0.00	0.005	0.013	0.39
NC=C1C=CC(=O)C=C1	54	960	10681	330568	16.040	0.00	0.005	0.003	1.74
Nc1nc(:c)c(:c:c)s1	51	2523	10684	329005	11.390	0.00	0.005	0.008	0.62
CCN1CSC(=C)C1=O	50	1106	10685	330422	5.395	0.02	0.005	0.003	1.40
CCn1ncccc1=O	50	1811	10685	329717	1.246	0.26	0.005	0.005	0.85
CC(=O)NCc1cccs1	48	1208	10687	330320	1.948	0.16	0.004	0.004	1.23
NC=C1C=CC=CC1=O	48	1690	10687	329838	0.807	0.37	0.004	0.005	0.88
c1nnc(n1)-c1ccccc1	46	2903	10689	328625	24.338	0.00	0.004	0.009	0.49
CCCc1cccn1C	44	212	10691	331316	166.486	0.00	0.004	0.001	6.41
NC(=O)c1cc(:c)cs1	43	1832	10692	329696	4.412	0.04	0.004	0.006	0.72
Clc1cnnc(=O)c1Cl	41	4	10694	331524	1146.521	0.00	0.004	0.000	316.55
c1nnc(o1)-c1ccccc1	41	1937	10694	329591	7.409	0.01	0.004	0.006	0.65
CNC1C2SCC(C)=C(C)N2C1=O	40	20	10695	331508	797.233	0.00	0.004	0.000	61.77
Cc1cncc(C)c1C	39	391	10696	331137	49.889	0.00	0.004	0.001	3.08
CN(C)c1ncnc(N)n1	39	919	10696	330609	2.761	0.10	0.004	0.003	1.31
CCN(C)C1CCCCC1	39	1522	10696	330006	2.102	0.15	0.004	0.005	0.79
COC(=O)c1ccco1	38	820	10697	330708	4.729	0.03	0.004	0.002	1.43
CSc1ccc(Cl)cc1	37	728	10698	330800	7.294	0.01	0.003	0.002	1.57
O=CCSc1ccccn1	36	1234	10699	330294	0.382	0.54	0.003	0.004	0.90

CCSCc1ccccc1	36	1601	10699	329927	4.757	0.03	0.003	0.005	0.69
CC1=C(N)OC(C)=CC1	35	244	10700	331284	81.354	0.00	0.003	0.001	4.43
FC(F)(F)c1ccnc(S)n1	34	304	10701	331224	53.370	0.00	0.003	0.001	3.45
c1nc2cncnc2n1	34	3016	10701	328512	41.403	0.00	0.003	0.009	0.35
c1coc(c1)-c1ncco1	33	136	10702	331392	149.508	0.00	0.003	0.000	7.49
c:c:c:c1cncn1:c	31	259	10704	331269	54.503	0.00	0.003	0.001	3.70
O=C\C=C\c1cccs1	31	422	10704	331106	20.515	0.00	0.003	0.001	2.27
Cc1ccnc2ccnn12	31	878	10704	330650	0.225	0.64	0.003	0.003	1.09
c1coc(c1)-c1ccnn1	30	92	10705	331436	184.891	0.00	0.003	0.000	10.07
CC1(C)CC(=O)CC(=O)C1	30	215	10705	331313	66.952	0.00	0.003	0.001	4.31
Cc1cc(C)n(C)c1C	30	1034	10705	330494	0.353	0.55	0.003	0.003	0.90
O=c1ccnc2ccccn12	30	1303	10705	330225	3.457	0.06	0.003	0.004	0.71
CCn1c(C)ccc1C	29	561	10706	330967	6.155	0.01	0.003	0.002	1.60
Cc1cc(C)cc(N)c1	29	1627	10706	329901	10.511	0.00	0.003	0.005	0.55
CCSCc1ccco1	28	32	10707	331496	374.288	0.00	0.003	0.000	27.02
COC1=CCC=CC1=O	27	460	10708	331068	9.305	0.00	0.003	0.001	1.81
Cn1nnc2ccccc12	27	582	10708	330946	3.378	0.07	0.003	0.002	1.43
CCc1sc(cc1C):n:c	27	904	10708	330624	0.172	0.68	0.003	0.003	0.92
COC(=O)c1cccs1	27	1241	10708	330287	4.249	0.04	0.003	0.004	0.67
Sc1nnnn1-c1ccccc1	25	729	10710	330799	0.080	0.78	0.002	0.002	1.06
Cc1cc(C)c(C)cn1	25	758	10710	330770	0.008	0.93	0.002	0.002	1.02
c:c:c1cnccc1:c	25	956	10710	330572	1.120	0.29	0.002	0.003	0.81
CCn1nc(C)cc1C	25	1013	10710	330515	1.816	0.18	0.002	0.003	0.76
CC(=C)\C=C\c1ccco1	24	17	10711	331511	414.243	0.00	0.002	0.000	43.60
Cc1ccc(o1)C(O)=O	24	329	10711	331199	15.601	0.00	0.002	0.001	2.25
NCCCn1ccnc1	23	1136	10712	330392	5.080	0.02	0.002	0.003	0.63
COC(=O)N1N(C)CC1=O	22	2	10713	331526	619.187	0.00	0.002	0.000	339.71
COC(=O)c1ccoc1C	22	302	10713	331226	14.250	0.00	0.002	0.001	2.25

CCSc1nnnn1C	22	546	10713	330982	1.017	0.31	0.002	0.002	1.24
OC1CCC=C(O1)C(=O)NCC#C	21	20	10714	331508	312.045	0.00	0.002	0.000	32.43
c1coc(c1)-c1cscn1	21	27	10714	331501	260.640	0.00	0.002	0.000	24.02
CC(=O)Nc1cccnc1	21	820	10714	330708	1.135	0.29	0.002	0.002	0.79
NC(=O)C1CCCNC1	21	2965	10714	328563	58.701	0.00	0.002	0.009	0.22
Cn1cnc(c1S)N(O)=O	20	11	10715	331517	384.457	0.00	0.002	0.000	56.15
Cn1csc2ccccc12	20	478	10715	331050	1.270	0.26	0.002	0.001	1.29
NCCc1ccccn1	20	766	10715	330762	0.909	0.34	0.002	0.002	0.81
CSc1nc(O)c(C(c2ccccc2)c2c(O)nc(SC)nc2=O)c(=O)n1	19	1	10716	331527	555.571	0.00	0.002	0.000	586.78
Cn1coc(:c)c1:c:c	19	984	10716	330544	5.109	0.02	0.002	0.003	0.60
COc1ccccc1NS(=O)=O	19	1122	10716	330406	8.157	0.00	0.002	0.003	0.52
CNS(=O)(=O)c1cccs1	19	1505	10716	330023	17.994	0.00	0.002	0.005	0.39
c1cc2ccccn2c1	18	203	10717	331325	18.258	0.00	0.002	0.001	2.74
Cc1ccccc1CS	18	458	10717	331070	0.653	0.42	0.002	0.001	1.21
Cc1ccnc1C(O)=O	18	712	10717	330816	1.083	0.30	0.002	0.002	0.78
NC(=O)NC1CCCCC1	18	1265	10717	330263	12.738	0.00	0.002	0.004	0.44
CC#Cc1ccccc1	17	140	10718	331388	30.586	0.00	0.002	0.000	3.75
Cc1nc(N)nc(N)n1	17	328	10718	331200	3.646	0.06	0.002	0.001	1.60
CCSc1nccnn1	17	574	10718	330954	0.132	0.72	0.002	0.002	0.91
c1nnc2cccnn12	17	836	10718	330692	3.681	0.06	0.002	0.003	0.63
CNc1nccc(C)n1	17	1086	10718	330442	9.269	0.00	0.002	0.003	0.48
NC(=O)CSc1nccn1	17	1409	10718	330119	17.818	0.00	0.002	0.004	0.37
ON(=O)C(=C)C(Cl)=C(Cl)Cl	16	4	10719	331524	388.950	0.00	0.001	0.000	123.53
CNc1nnc(s1)S(=O)(=O)NC	16	302	10719	331226	3.762	0.05	0.001	0.001	1.64
Sc1nc2ccccc2s1	16	658	10719	330870	1.293	0.26	0.001	0.002	0.75
CS(=O)(=O)Cc1ccco	15	68	10720	331460	60.959	0.00	0.001	0.000	6.81

1									
CNN1C(=O)CSC1=S	15	104	10720	331424	35.129	0.00	0.001	0.000	4.45
Oc1ccc(cc1)N=C	15	135	10720	331393	23.269	0.00	0.001	0.000	3.43
CN(C(C)=O)c1c(N)n(C)c(=O)nc1=O	15	1007	10720	330521	9.396	0.00	0.001	0.003	0.46
Nc1cncn1:c:c:c	14	395	10721	331133	0.111	0.74	0.001	0.001	1.09
c1nnc2ncncc12	14	1318	10721	330210	19.142	0.00	0.001	0.004	0.33
CSc1nnc(-c2ccco2)c(n1)-c1ccco1	13	1	10722	331527	370.961	0.00	0.001	0.000	401.48
Cc1cc(=O)c(O)co1	13	212	10722	331316	5.170	0.02	0.001	0.001	1.89
CC1=C(N)Oc2nncc2C1	13	261	10722	331267	2.334	0.13	0.001	0.001	1.54
CNC(C)CCCCN	13	391	10722	331137	0.009	0.93	0.001	0.001	1.03
Cc1cc2cccnc2s1	13	675	10722	330853	3.528	0.06	0.001	0.002	0.59
Cc1nc(oc1C)-c1cccs1	12	21	10723	331507	119.933	0.00	0.001	0.000	17.65
CC=C1C(C)CCC=C1C	12	30	10723	331498	89.446	0.00	0.001	0.000	12.35
ON(=O)c1ccccn1	12	156	10723	331372	8.880	0.00	0.001	0.000	2.38
c1csc(c1)-c1ccccc1	12	855	10723	330673	8.786	0.00	0.001	0.003	0.43
Clc1ncsc1\C=N\NC=O	11	4	10724	331524	243.301	0.00	0.001	0.000	84.93
O=C1NC(=S)SC1=C\C=C\c1ccco1	11	0	10724	331528	339.723	0.00	0.001	0.000	0.00
